# Comprehensive assessment of immune context and immunotherapy response via noninvasive imaging in gastric cancer

**DOI:** 10.1172/JCI175834

**Published:** 2024-01-25

**Authors:** Zepang Sun, Taojun Zhang, M. Usman Ahmad, Zixia Zhou, Liang Qiu, Kangneng Zhou, Wenjun Xiong, Jingjing Xie, Zhicheng Zhang, Chuanli Chen, Qingyu Yuan, Yan Chen, Wanying Feng, Yikai Xu, Lequan Yu, Wei Wang, Jiang Yu, Guoxin Li, Yuming Jiang

**Affiliations:** 1 Department of General Surgery & Guangdong Provincial Key Laboratory of Precision Medicine for Gastrointestinal Tumor, Nanfang Hospital, Southern Medical University, Guangzhou, Guangdong, China.; 2 Department of Surgery and; 3 Department of Radiation Oncology, Stanford University School of Medicine, Stanford, California, USA.; 4 College of Computer Science, Nankai University, Tianjin, China.; 5 Department of Gastrointestinal Surgery, Guangdong Provincial Hospital of Chinese Medicine, The Second Affiliated Hospital of Guangzhou University of Chinese Medicine, Guangzhou, China.; 6 Graduate Group of Epidemiology, UCD, Davis, California, USA.; 7 JancsiTech and Shenzhen Institute of Advanced Technology, Chinese Academy of Sciences, Shenzhen, China.; 8 Department of Medical Imaging Center, Nanfang Hospital, Southern Medical University, Guangzhou, China.; 9 Shenzhen Hospital of Integrated Traditional Chinese and Western Medicine, Shenzhen, China.; 10 Department of Pathology, School of Basic Medical Sciences, Southern Medical University, Guangzhou, China.; 11 The Department of Statistics and Actuarial Science, The University of Hong Kong, HKSAR, Hong Kong, China.; 12 Department of Gastric Surgery, and State Key Laboratory of Oncology in South China, Collaborative Innovation Center for Cancer Medicine, Sun Yat-sen University Cancer Center, Guangzhou, China.; 13 Beijing Tsinghua Changgung Hospital, School of Clinical Medicine, Tsinghua University, Beijing, China.; 14 Department of Radiation Oncology, Wake Forest University School of Medicine, Winston-Salem, North Carolina, USA.

**Keywords:** Gastroenterology, Immunology, Diagnostic imaging, Gastric cancer, Immunotherapy

## Abstract

**BACKGROUND:**

The tumor immune microenvironment can provide prognostic and therapeutic information. We aimed to develop noninvasive imaging biomarkers from computed tomography (CT) for comprehensive evaluation of immune context and investigate their associations with prognosis and immunotherapy response in gastric cancer (GC).

**METHODS:**

This study involved 2,600 patients with GC from 9 independent cohorts. We developed and validated 2 CT imaging biomarkers (lymphoid radiomics score [LRS] and myeloid radiomics score [MRS]) for evaluating the IHC-derived lymphoid and myeloid immune context respectively, and integrated them into a combined imaging biomarker [LRS/MRS: low(−) or high(+)] with 4 radiomics immune subtypes: 1 (−/−), 2 (+/−), 3 (−/+), and 4 (+/+). We further evaluated the imaging biomarkers’ predictive values on prognosis and immunotherapy response.

**RESULTS:**

The developed imaging biomarkers (LRS and MRS) had a high accuracy in predicting lymphoid (AUC range: 0.765–0.773) and myeloid (AUC range: 0.736–0.750) immune context. Further, similar to the IHC-derived immune context, 2 imaging biomarkers (HR range: 0.240–0.761 for LRS; 1.301–4.012 for MRS) and the combined biomarker were independent predictors for disease-free and overall survival in the training and all validation cohorts (all *P* < 0.05). Additionally, patients with high LRS or low MRS may benefit more from immunotherapy (*P* < 0.001). Further, a highly heterogeneous outcome on objective response ​rate was observed in 4 imaging subtypes: 1 (−/−) with 27.3%, 2 (+/−) with 53.3%, 3 (−/+) with 10.2%, and 4 (+/+) with 30.0% (*P* < 0.0001).

**CONCLUSION:**

The noninvasive imaging biomarkers could accurately evaluate the immune context and provide information regarding prognosis and immunotherapy for GC.

## Abstract 2

Noninvasive CT imaging biomarkers accurately evaluates the IHC-derived lymphoid and myeloid immune context, providing information regarding prognosis and immunotherapy for gastric cancer.

## Graphical abstract

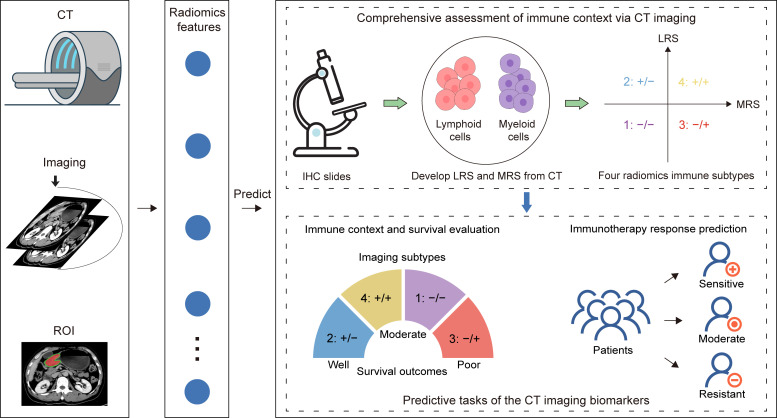

## Introduction

Recently, the tumor immune microenvironment (TIME) has attracted increasing research attention (1). A growing body of literature highlights its crucial role in cancer progression and therapeutic responses (1–3). In particular, changes in abundance of lymphoid and myeloid cells, both as main components of the TIME, are closely correlated with prognosis and immunotherapy response in various cancers (4–9). Gastric cancer (GC) is the fifth most common malignancy and the third leading cause of cancer-related death worldwide (10). In recent years, immunotherapy has emerged as an additional treatment option to improve the survival of patients (11–13). Importantly, an encouraging anti–PD-1 therapeutic outcome in GC has been reported by the Checkmate-649 trial (14). However, high variability in the benefit of immunotherapy has been observed in patients with GC, which indicates a remaining opportunity to improve individual decision making (14–17). Identification of the lymphoid and myeloid TIME at diagnosis can determine the response to immunotherapy (6–8, 18, 19). Previous studies reported a positive effect of the lymphoid immune context and a negative effect of the myeloid immune context on prognosis and immunotherapy response (6–8, 20). Hence, evaluation of the immune context may be helpful to inform individualized decision making for immunotherapy.

Currently, assessment of immune infiltration in the TIME mainly depends on immunohistochemical staining or bulk-tissue sequencing (21–24). Nevertheless, both methods require substantial tissue specimens obtained from invasive procedures, which might not be appropriate for patients receiving neoadjuvant therapy or those with metastatic disease. Moreover, tumor biopsies may be influenced by spatial heterogeneity within a small sample and fail to achieve longitudinal real-time dynamic monitoring of immune signaling. Therefore, this approach may be insufficient for the accurate evaluation of lymphoid and myeloid cells in the TIME (25–28). Thus, it was recently proposed that peripheral blood lymphoid and myeloid cells can be used to describe the TIME (29, 30). However, considering the volatility of immune cells’ abundance in peripheral blood and susceptibility to interference from individual and environmental factors, it cannot represent the real immune context. Thus, development and validation of a noninvasive approach for accurate and dynamic assessment of the immune context are clearly valuable and long overdue.

Radiomics is an emerging field that converts medical images into mineable quantitative data by acquiring multidimensional imaging features (31, 32). It is currently proposed as a digital biopsy for noninvasive tumor evaluation (33, 34). Accumulating evidence has suggested computed tomography (CT) image–based features are closely associated with temporal and spatial heterogeneity of the tumor and the TIME (34, 35). Particularly, the combination of intratumoral and peritumoral imaging features will increase our knowledge regarding tumor biology and the immune microenvironment, followed by contribution to prognosis prediction and immunotherapy decision making (31, 33, 36), as they can better demonstrate diverse spectrum or activation status of tumor-infiltrating immune cells. On this basis, several studies have confirmed a strong relationship between imaging features and tumor-infiltrating immune cells (36–38). However, to our knowledge, the noninvasive radiomics approach to evaluate lymphoid and myeloid immune context has not been reported, and studies on disclosing the association between genomics characterization and imaging appearance are lacking.

This study aims to develop and validate noninvasive imaging biomarkers for evaluation of the lymphoid and myeloid immune context based on intratumoral and peritumoral CT radiomics features in patients with GC. Using the Shapley value and transcriptome sequencing data, we provided explanations to the imaging biomarkers and determined relationships between genomics characterization and imaging appearance. We further evaluated their predictive values on prognosis and anti–PD-1 immunotherapy response.

## Results

### Clinicopathological characteristics.

**Figure 1:**
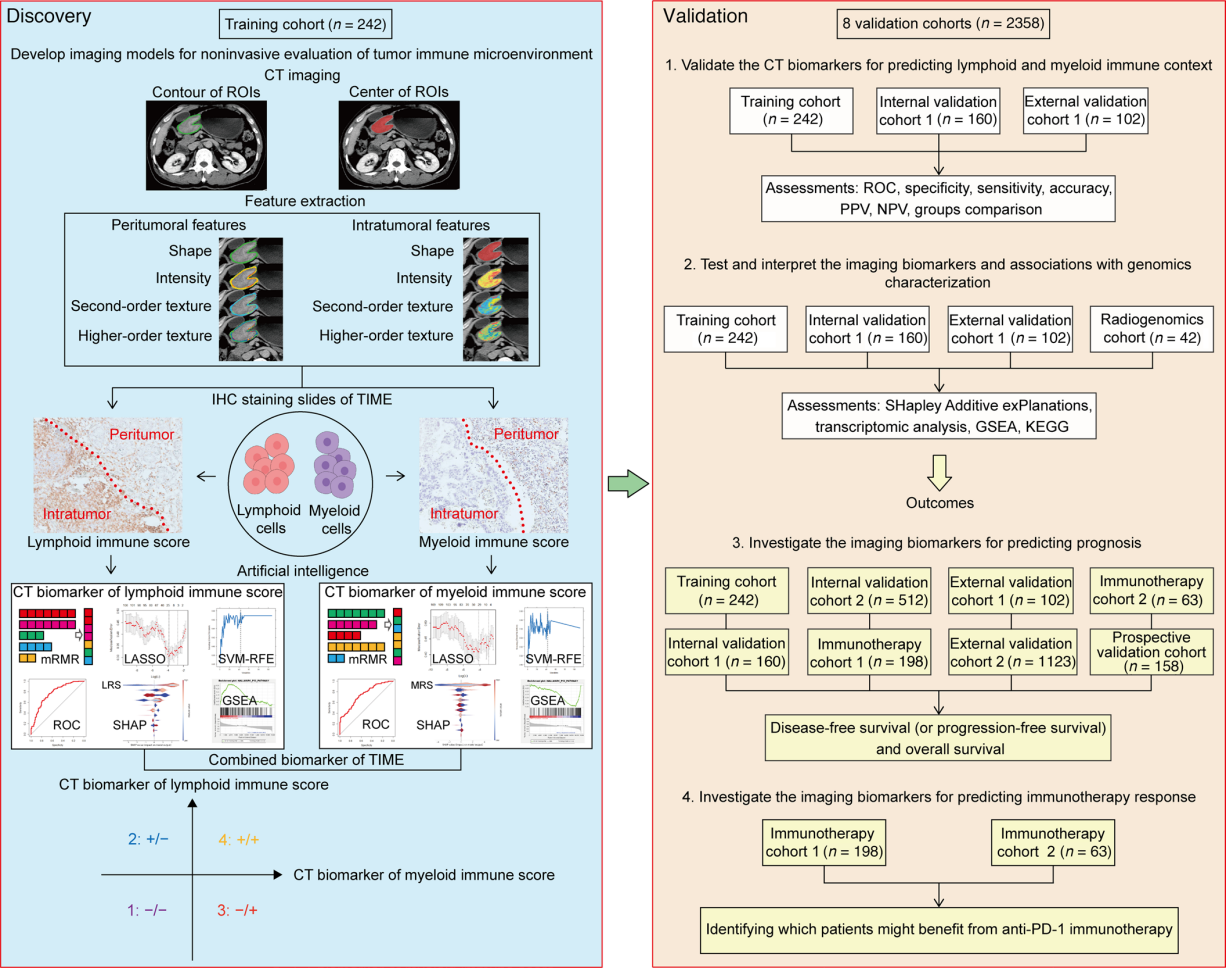
Study design for the discovery and validation of the radiomics imaging biomarkers for the lymphoid and myeloid immune context in gastric cancer. The training cohort, 2 internal validation cohorts (1 and 2), the prospective validation cohort, and the immunotherapy cohort 1 were recruited from Nanfang Hospital of Southern Medical University. Two external validation cohorts (1 and 2) were obtained from Sun Yat-sen University Cancer Center. The radiogenomics cohort was from the Cancer Immunome Atlas and the Cancer Genome Atlas database of the US. The immunotherapy cohort 2 was retrospectively registered from Guangdong Provincial Hospital of Chinese Medicine.

The present study included 2,600 patients with GC from 9 independent cohorts at 4 different centers. The overall study design is shown in Figure 1. Patients with available information on CT images and IHC or available follow-up data (*n* = 2,297) were selected to evaluate the lymphoid and myeloid immune context, or predict survival. Moreover, patients with available radiogenomics information (*n* = 42) were used to determine relationships between genomics characterization and imaging appearance. Furthermore, patients treated with anti–PD-1 immunotherapy (*n* = 261) were used to investigate associations between imaging biomarkers and therapeutic outcomes. The clinicopathological characteristics of patients in the training cohort (*n* = 242), internal validation cohort 1 (*n* = 160), internal validation cohort 2 (*n* = 512), external validation cohort 1 (*n* = 102), external validation cohort 2 (*n* = 1,123), and prospective validation cohort (*n* = 158) are listed in Table 1. Of these patients, 1,574 (68.5%) were men, and 723 patients (31.5%) were women. The median age (interquartile range) for men was 58.0 (50.0–65.0) years, and the median age (interquartile range) for women was 55.0 (43.0–63.0) years. Most patients (*n* = 1,651, 71.9%) had stage II or III disease.

Supplemental Table 1 (supplemental material available online with this article; https://doi.org/10.1172/JCI175834DS1) lists the detailed clinicopathological features of the immunotherapy cohort 1 (*n* = 198), immunotherapy cohort 2 (*n* = 63), and radiogenomics cohort (*n* = 42). Among the 2 immunotherapy cohorts, 149 patients (57.1%) were men, and 112 patients (42.9%) were women. The median age (interquartile range) for men was 60.0 (52.0–67.0) years, and the median age (interquartile range) for women was 51.0 (41.0–63.0) years. Almost all patients had stage III or IV disease, except for 17 patients with stage II GC. Immunotherapy as first-, second-, and third-line treatment was administered in 147, 72, and 42 patients, respectively. The objective response (OR) rates in the immunotherapy cohort 1 and 2 were 32.3% and 19.1%, respectively. In the radiogenomics cohort, 36 patients (85.7%) were men, and 6 patients (14.3%) were women. The median age (interquartile range) for men was 68.0 (59.0–72.0) years, and the median age (interquartile range) for women was 66.0 (52.0–68.0) years. Most patients (*n* = 39; 90.5%) had stage II or III disease.

### Lymphoid and myeloid immune context were significantly associated with prognosis.

**Figure 2:**
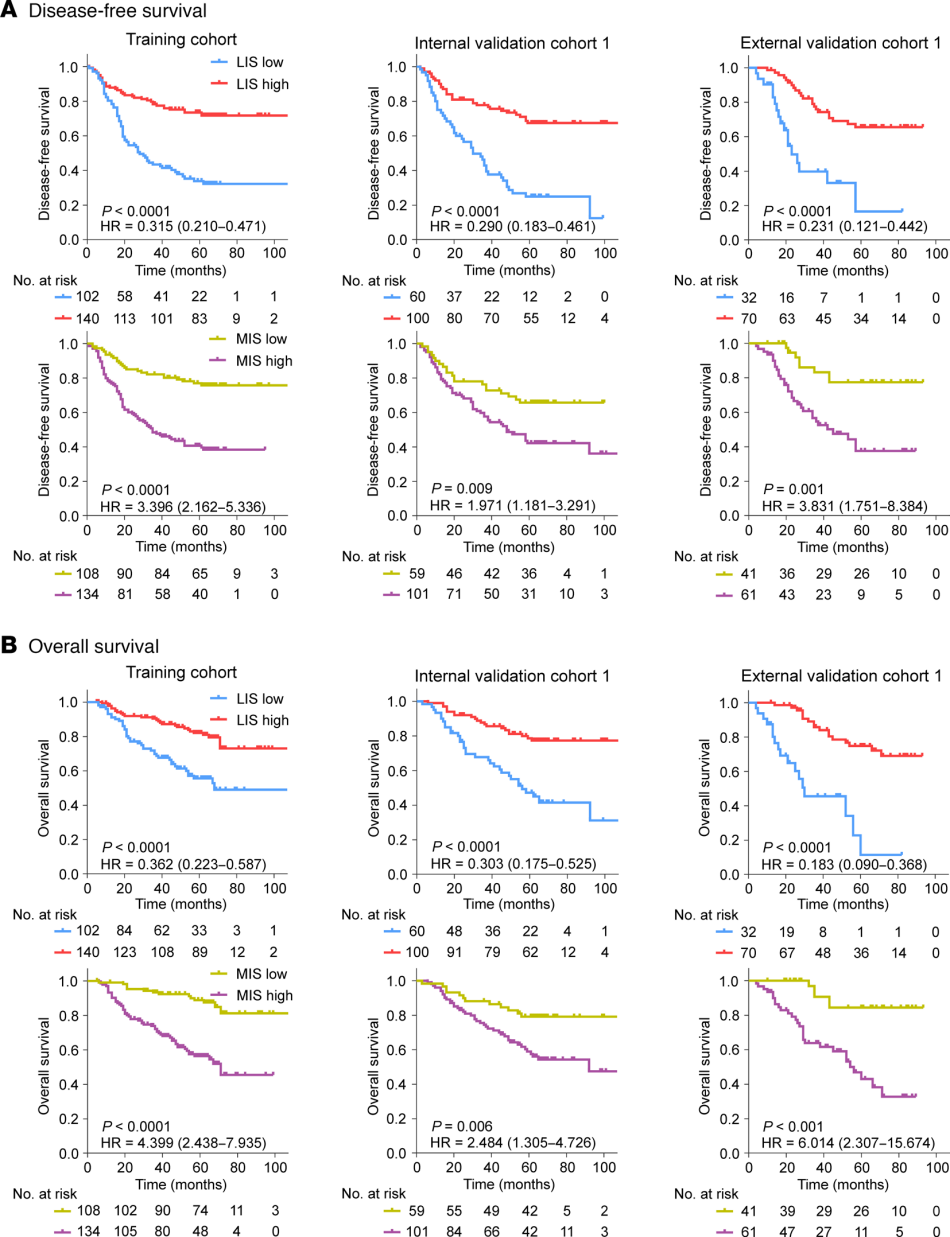
Lymphoid and myeloid immune context were significantly associated with prognosis in the training and validation cohorts. (**A**) Disease-free survival; (**B**) Overall survival. Comparisons of the above survival curves were performed with a 2-sided log-rank test. LIS, lymphoid immune score; MIS, myeloid immune score.

This study initially evaluated the predictive power of the lymphoid and myeloid immune context, determined through IHC, for survival outcomes. The Kaplan-Meier plots of disease-free survival (DFS) and overall survival (OS) are shown in Figure 2. Patients with a high lymphoid immune score (LIS) or low myeloid immune score (MIS) had a better prognosis (DFS and OS) in the training cohort and 2 validation cohorts (*P* < 0.01 for all with statistical significance). The relationships between the LIS or MIS status and clinicopathological characteristics in each cohort are presented in Supplemental Tables 2 and 3. Multivariate Cox regression analyses also confirmed that, after adjusting for other clinicopathological variables, the LIS (HR: 0.183–0.362) and MIS (HR: 1.971–6.014) remained independent predictive factors for clinical outcomes (DFS and OS) (Supplemental Tables 4–7). Further, the comparison of specific scores in each LIS or MIS status (i.e., 0 versus 1 in the LIS-low group; 2 versus 3 versus 4 in the LIS-high group; and 1 versus 2 in the MIS-high group), did not reveal statistically significant differences in survival (all *P* > 0.1) (Supplemental Figures 1 and 2).

### Development and validation of the radiomics imaging biomarkers.

**Figure 3:**
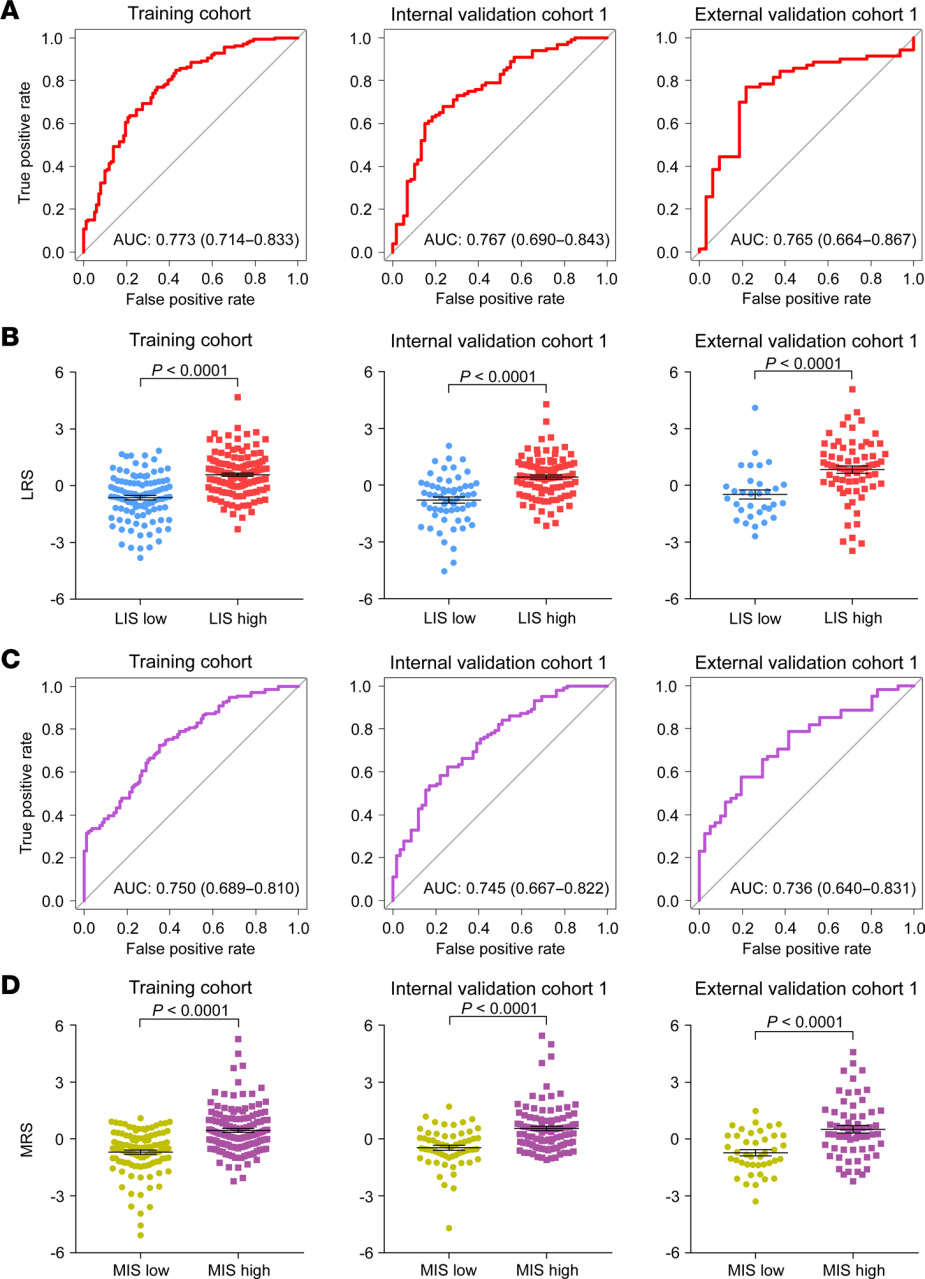
Predicted performance of radiomics imaging biomarkers for lymphoid and myeloid immune context in the training cohort, internal validation cohort 1, and external validation cohort 1. (**A**) Receiver operating characteristic curves of the LRS for predicting the lymphoid immune context; (**B**) LRS of the high and low lymphoid immune context; (**C**) Receiver operating characteristic curves of the MRS for predicting the myeloid immune context; (**D**) MRS of the high and low myeloid immune context. The data are presented as the mean values with SEM. For statistical comparisons among different groups in the training cohort (*n* = 242), internal validation cohort 1 (*n* = 160), and external validation cohort 1 (*n* = 102), a 2 tailed *t* test (unpaired) was used. LRS, lymphoid radiomics score; MRS, myeloid radiomics score.

In the training cohort, using several artificial intelligence algorithms, including max-relevance and min-redundancy (mRMR) algorithm, collinearity reduction algorithm, the least absolute shrinkage and selector operation (LASSO) logistic regression algorithm, the support vector machine-recursive feature elimination (SVM-RFE) algorithm, and multivariate logistic regression method (MLR), we constructed 2 imaging biomarkers, called lymphoid radiomics score (LRS) and myeloid radiomics score (MRS), to evaluate the lymphoid and myeloid immune context, respectively. The detailed workflow and calculation formulas are shown in Supplemental Figure 3. The LRS radiomics biomarker included 7 intratumoral and 4 peritumoral features, while the MRS radiomics biomarker included 10 intratumoral and 4 peritumoral features. As shown in Figure 3, the AUC (95% CI) for the LRS in distinguishing the lymphoid immune status was 0.773 (0.714–0.833) in the training cohort, 0.767 (0.690–0.843) in the internal validation cohort 1, and 0.765 (0.664–0.867) in the external validation cohort 1. Moreover, a significantly higher LRS was observed in the LIS-high group than that in the LIS-low group within each cohort. In addition, the AUC (95% CI) for the MRS in distinguishing the myeloid immune status was 0.750 (0.689–0.810), 0.745 (0.667–0.822), and 0.736 (0.640–0.831) in the training cohort, internal validation cohort 1, and external validation cohort 1, respectively, followed by a significantly higher MRS observed in the MIS-high group than that in the MIS-low group. We also confirmed that the AUC values of these 2 imaging biomarkers were higher than that of any single radiomics feature in the training and validation cohorts (Supplemental Figure 4). The optimal cut-off value for LRS and MRS identified by Youden’s index in the training cohort was –0.1293 and –0.2604, respectively (Supplemental Table 8). Accordingly, patients were classified into different radiomics status: a LRS-low group (LRS < –0.1293) or a LRS-high group (LRS ≥ –0.1293), and a MRS-low group (MRS < –0.2604) or a MRS-high group (MRS ≥ –0.2604). The relationships between the LRS or MRS status and clinicopathological characteristics in each cohort are listed in Supplemental Tables 9–12. Furthermore, the LRS and MRS statuses were incorporated into a combined imaging biomarker (LRS/MRS: low or high) including 4 radiomics immune subtypes: 1 (−/−), 2 (+/−), 3 (−/+), and 4 (+/+). And the clinicopathological characteristics stratified by this combined biomarker in all cohorts are reported in Supplemental Tables 13–15. Although the determination of a cutoff for the CT biomarkers was not the aim of this study, comparative data of these cut-off values based on different methods (Youden’s index, median, upper quartile, or lower quartile) are presented in the Supplemental Materials and Supplemental Table 16.

### Explanations via Shapley additive explanations and RNA-Seq.

**Figure 4:**
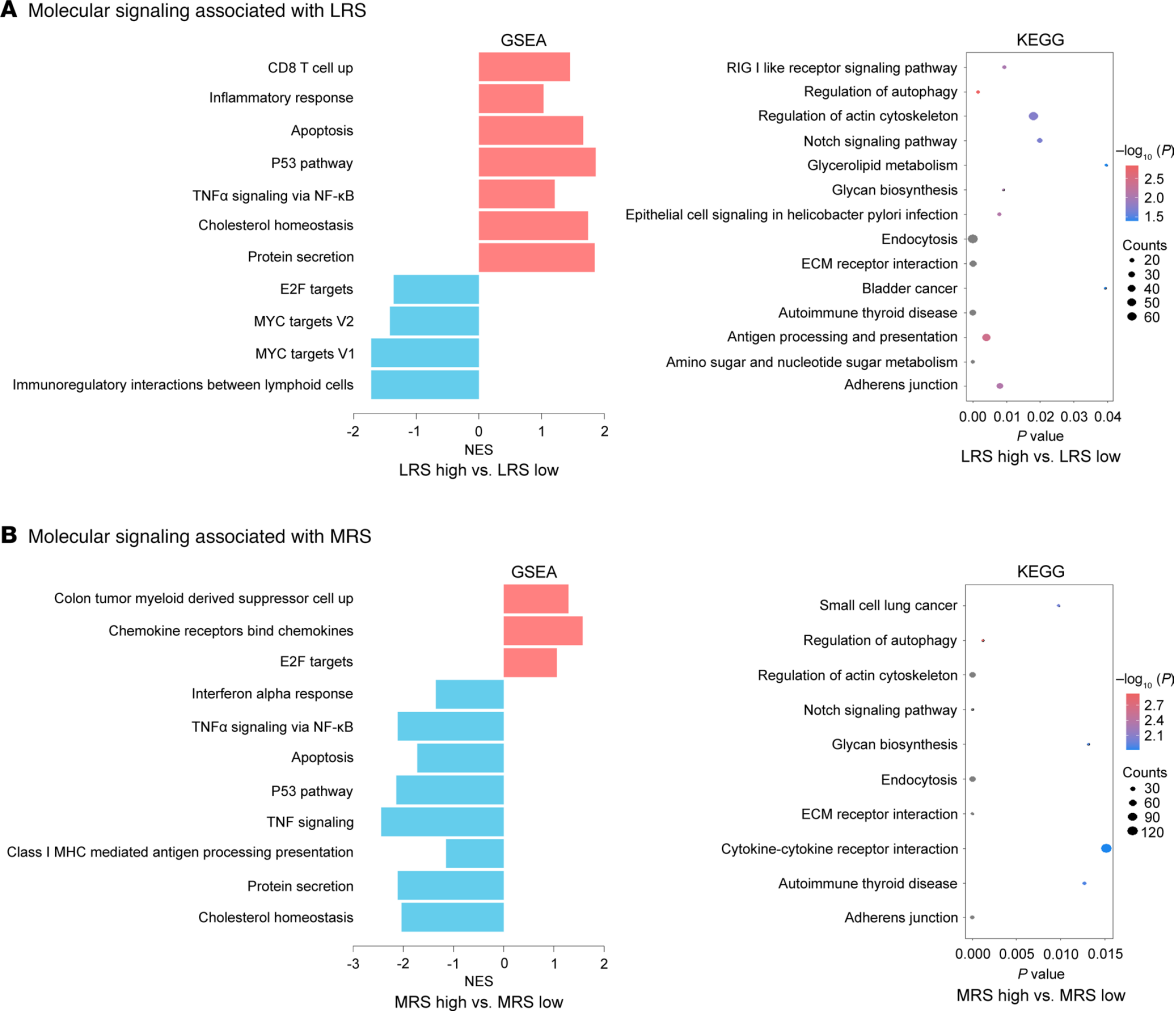
Radiogenomics interpretations on the relationship between genomics characterization and imaging appearance. (**A**) Molecular signaling pathways associated with LRS in the radiogenomics cohort (*n* = 42); (**B**) Molecular signaling pathways associated with MRS in the radiogenomics cohort (*n* = 42). The *P* value was calculated using permutation test, adjusted for multiple hypothesis testing. LRS, lymphoid radiomics score; MRS, myeloid radiomics score.

Risk estimates can be extracted from the prediction by Shapley values (positively correlated with importance) then to allow explanation on the global level. Compared with other clinicopathological variables (mean Shapley value: 0.01–0.88), the LRS (mean Shapley value: 1.80–2.08) and MRS (mean Shapley value: 1.60–1.95) were the most important features for the prediction of the lymphoid and myeloid immune context (Supplemental Figure 5). We also found that the 4 radiomics imaging subtypes (1 [−/−], 2 [+/−], 3 [−/+], and 4 [+/+]) were the most important features for the prediction of the lymphoid and myeloid immune context, compared with other clinicopathological variables (Supplemental Figure 6A). And the imaging subtype 1 (−/−) was characterized by low infiltration of lymphoid cells and myeloid cells; the imaging subtype 2 (+/−) was characterized by high infiltration of lymphoid cells and low infiltration of myeloid cells; the imaging subtype 3 (−/+) was characterized by low infiltration of lymphoid cells and high infiltration of myeloid cells; the imaging subtype 4 (+/+) was characterized by high infiltration of lymphoid cells and myeloid cells (Supplemental Figure 6B). Next, we performed GSEA and KEGG analyses using GSEA software to investigate the molecular signaling pathways associated with imaging biomarkers in a radiogenomics cohort. The results showed that the LRS-high and MRS-low groups were positively correlated with multiple tumor suppression–related pathways and immune activation–related pathways, such as the P53 pathway, apoptosis pathway, inflammatory response pathway, antigen processing and presentation pathway, and TNF signaling, while the LRS-low and MRS-high groups were positively correlated with multiple tumor promoting–related pathways and metabolism-related pathways, including MYC target signaling, E2F target signaling, and glucolipid metabolism (Figure 4 and Supplemental Figure 7). These findings suggested that the developed radiomics imaging biomarkers had potential predictive value for prognosis and therapeutic response.

### Prognostic value of the radiomics imaging biomarkers.

**Figure 5:**
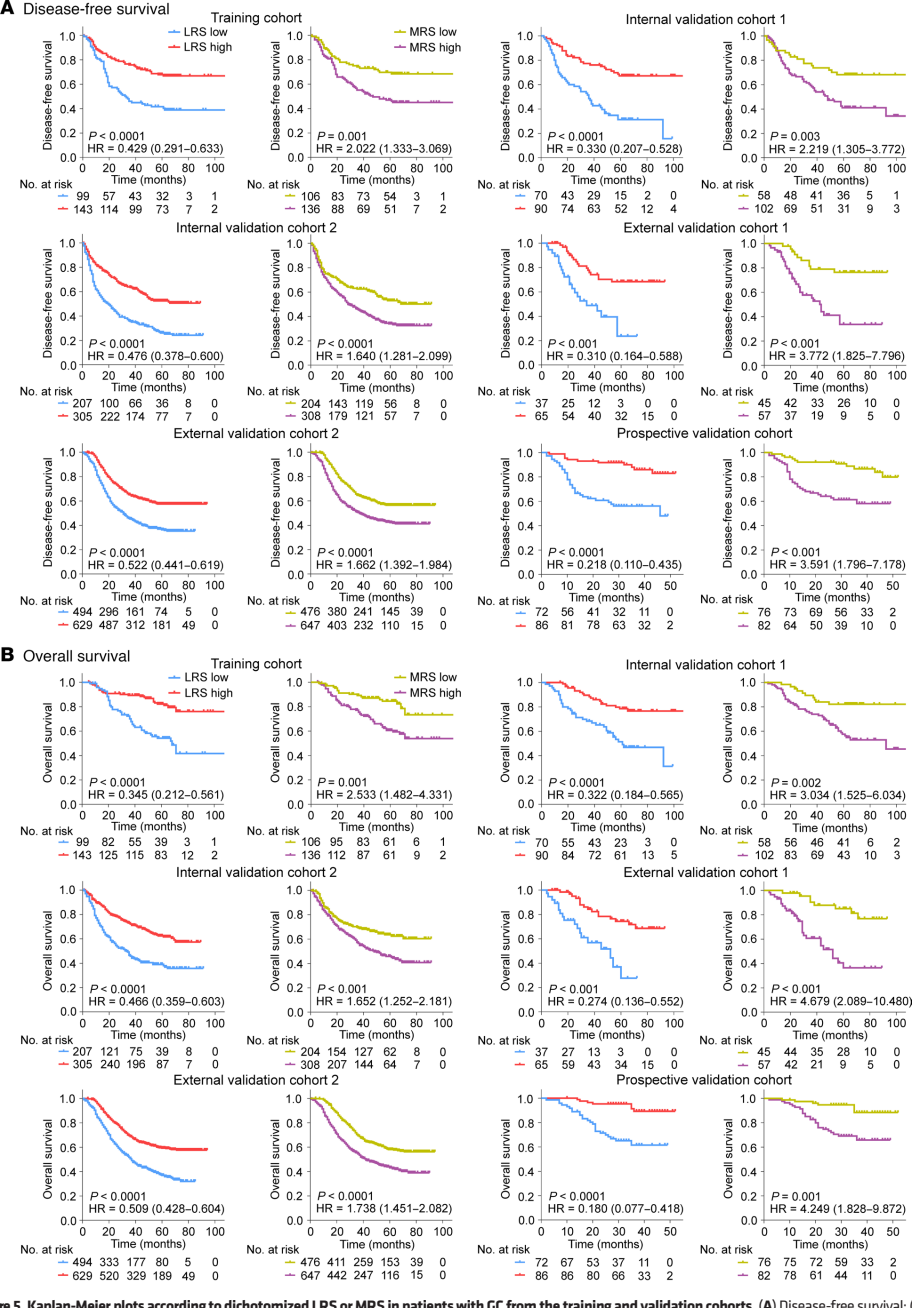
Kaplan-Meier plots according to dichotomized LRS or MRS in patients with GC from the training and validation cohorts. (**A**) Disease-free survival; (**B**) Overall survival. Comparisons of the above survival curves were performed with a 2-sided log-rank test. LRS, lymphoid radiomics score; MRS, myeloid radiomics score.

**Figure 6:**
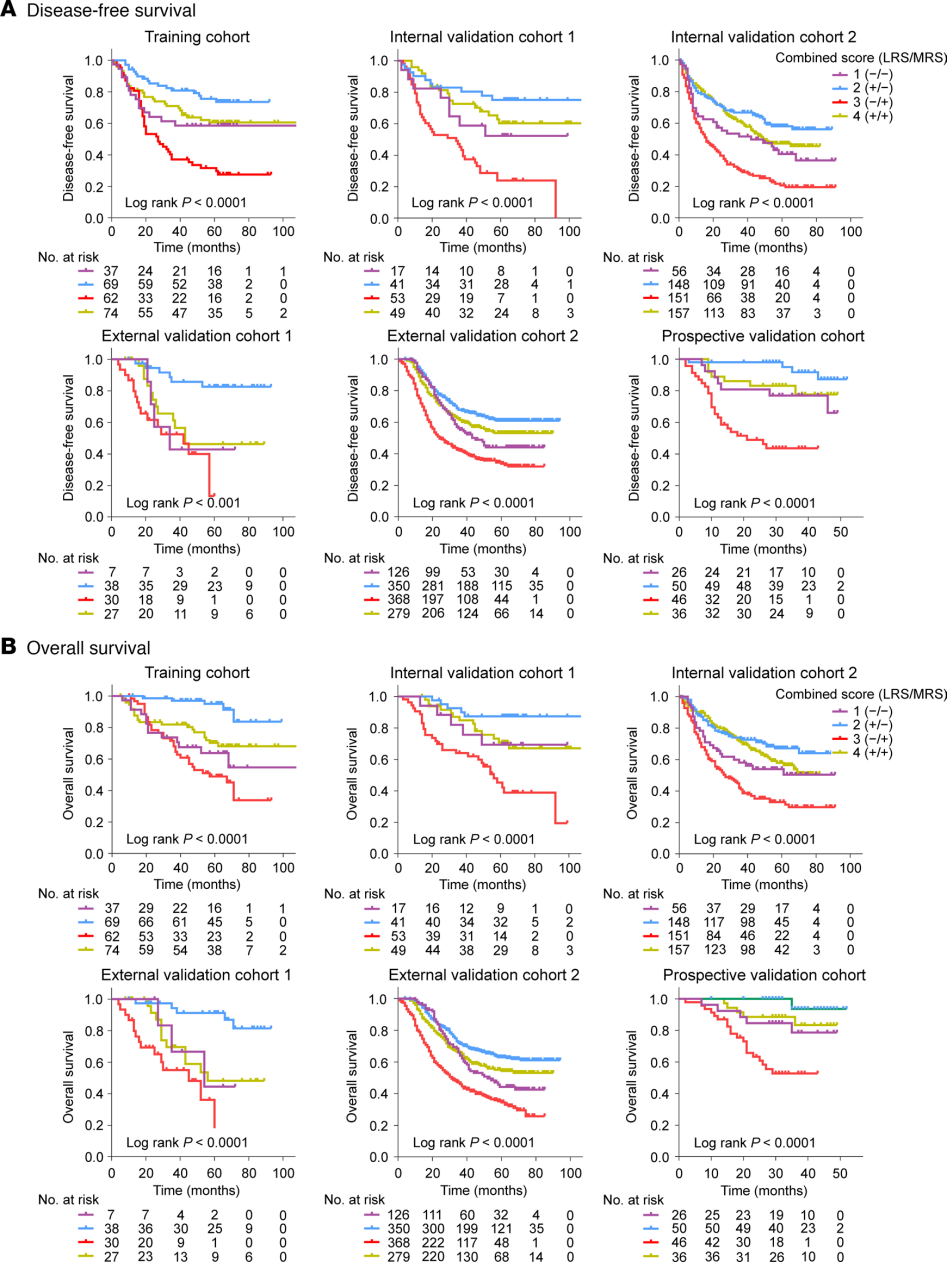
Kaplan-Meier analyses of prognosis according to the combined imaging biomarker (LRS/MRS: low or high) with 4 radiomics immune subtypes: 1 (−/−), 2 (+/−), 3 (−/+), and 4 (+/+) in patients with GC. (**A**) DFS in the training cohort, 2 internal validation cohorts; 2 external validation cohorts, and prospective validation cohort; (**B**) OS in the training cohort, 2 internal validation cohorts; 2 external validation cohorts, and prospective validation cohort. Comparisons of the above survival curves were performed with a 2-sided log-rank test. LRS, lymphoid radiomics score; MRS, myeloid radiomics score; DFS, disease-free survival; OS, overall survival.

The radiomics imaging biomarkers of LRS (HR: 0.180–0.522, *P* < 0.001) and MRS (HR: 1.640–4.679, *P* < 0.004) were significantly associated with survival outcomes (DFS and OS) in the training cohort, 2 internal validation cohorts, 2 external validation cohorts, and the prospective validation cohort (Figure 5). The covariates in the multivariate Cox regression analyses included age, sex, tumor location, tumor differentiation, tumor size, Lauren type, CEA, CA19-9, chemotherapy, and TNM staging. The multivariate analyses confirmed that LRS and MRS remained independent predictive factors for clinical outcomes (DFS and OS) after adjusting for these covariates. (Table 2 and Supplemental Tables 17–22). We next observed that the proportional hazards (PH) assumption tests for the Cox regression models were valid for OS and DFS (Supplemental Figure 8, *P* > 0.05). Moreover, survival curves showed that the combined biomarker (LRS/MRS) was the independent classifier for DFS (*P* < 0.0001) and OS (*P* < 0.0001) in each cohort (Figure 6). Of these patients, the highest 5-year DFS and OS rates were observed in the imaging subtype 2 (+/−) (52.7% and 60.5%, respectively), followed by the subtype 4 (+/+) (39.0% and 45.1%, respectively) and subtype 1 (−/−) (35.9% and 40.6%, respectively), while the 5-year DFS and OS rates in the imaging subtype 3 (−/+) (15.9% and 21.1%, respectively) was the worst (*P* < 0.0001 for all). We observed a slight survival difference of imaging subtypes 1 and 4 in external validation cohort 1 compared with other cohorts, which may be due to a bias of small sample size in this cohort. Additionally, when stratified by other factors including TNM stage, age, sex, tumor size, location, histology, differentiation, CEA, and CA19-9, 2 radiomics imaging biomarkers and the combined biomarker maintained their statistically significant predictive value for prognosis in these subgroups (Supplemental Figures 9–14). The aforementioned survival outcomes (Supplemental Figure 15) were also confirmed in a cohort from the USA, and the relationships between these 3 imaging biomarkers and clinicopathological variables are reported in Supplemental Table 23. Finally, the nomograms integrating the radiomics biomarkers and TNM stage for predicting prognosis of DFS and OS were developed (Supplemental Figure 16). As shown in Supplemental Table 24, we found that the nomogram consistently improved the accuracy of prognosis prediction, with a C-index ranging from 0.715–0.841, which was notably superior to the radiomics biomarkers (LRS and MRS) and TNM stage across all cohorts (*P* < 0.001).

### Predictive value of the radiomics imaging biomarkers for immunotherapy response.

**Figure 7:**
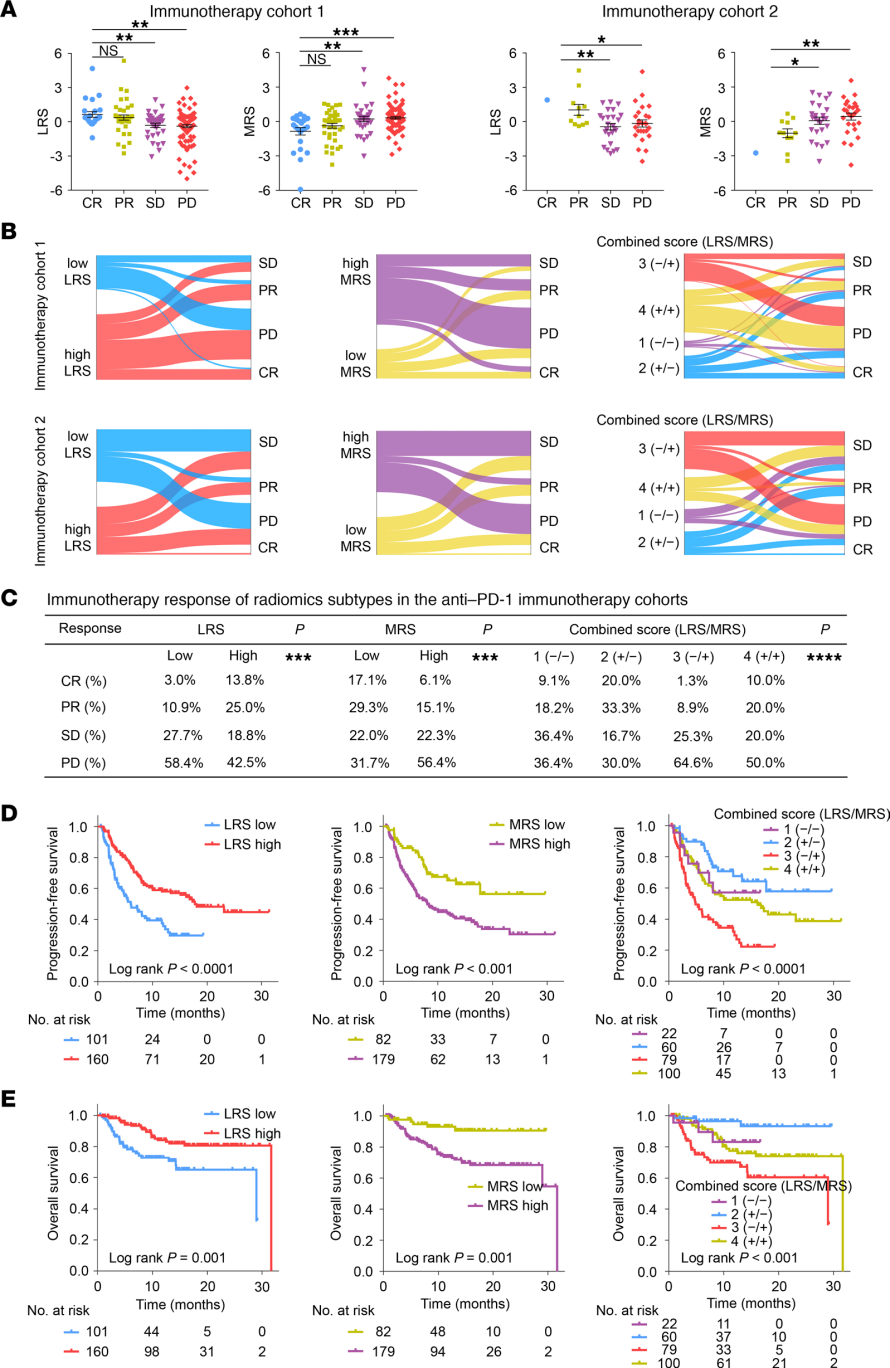
Predictive value of radiomics imaging biomarkers for therapeutic response and clinical outcomes in patients treated with anti–PD-1 immunotherapy. (**A**) LRS and MRS of different responses to anti–PD-1 immunotherapy in immunotherapy cohort 1 (*n* = 198) and 2 (*n* = 63). The data are presented as the mean values with SEM. For statistical comparisons among different groups, a 2 tailed *t* test (unpaired) was used.; (**B**) The ratio of different immunotherapy responses among subgroups of imaging biomarkers in the immunotherapy cohort 1 (*n* = 198) and 2 (*n* = 63); (**C**) The ratio of different immunotherapy responses from 2 radiomics imaging biomarkers (LRS and MRS) and their combined biomarker (LRS/MRS) with 4 subtypes in the entire cohort (*n* = 261). Data was compared by the κ^2^ test; (**D**) Prognostic value of the radiomics imaging biomarkers for progression-free survival in patients treated with anti–PD-1 immunotherapy; (**E**) Prognostic value of the radiomics imaging biomarkers for OS in patients treated with anti–PD-1 immunotherapy. Comparisons of the above survival curves were performed with a 2-sided log-rank test. ns, *P* > 0.05; *P < 0.05; **P < 0.01; ***P < 0.001; ****P < 0.0001. LRS, lymphoid radiomics score; MRS, myeloid radiomics score.

This study subsequently assessed the relationships between the radiomics imaging biomarkers and immunotherapy response in 2 independent cohorts from different centers. Interestingly, as shown in Figure 7A, we found that the LRS in the PR group (mean [95%CI] in immunotherapy cohort 1 and 2 was 0.377 [−0.078, 0.833], and 1.023 [−0.025, 2.082]) and the CR group (0.641 [0.145, 1.138]) was significantly higher than that in the PD group (−0.373 [−0.647, −0.100], and −0.154 [−0.807, 0.499]) and SD group (−0.303 [−0.695, 0.089], and −0.436 [−1.011, 0.136]), while the MRS in the PR group (−0.377 [−0.796, −0.042], and −1.028 [−1.884, −0.173]) and CR group (−0.851 [−1.514, −0.188]) was significantly lower than that in the PD group (0.312 [0.118, 0.506], and 0.418 [−0.211, 1.048]) and SD group (0.223 [−0.251, 0.697], and 0.056 [−0.584, 0.695]) in 2 immunotherapy cohorts (all *P* < 0.03).

More interestingly, we found that patients in the LRS-high group (40.8% and 30.0% in immunotherapy cohorts 1 and 2, respectively) or MRS-low group (51.7% and 33.4%) had a higher OR rate than those in the LRS-low group (16.2% and 9.1%) or MRS-high group (24.4% and 10.3%), which was also confirmed in the entire cohort (*P* < 0.001) (Figure 7B and Supplemental Tables 25 and 26). Moreover, a highly heterogeneous outcome on OR rate was also observed among the 4 imaging subtypes: 1 (−/−) with 27.3%, 2 (+/−) with 53.3%, 3 (−/+) with 10.2%, and 4 (+/+) with 30.0% (*P* < 0.0001), which was also confirmed in each immunotherapy cohort (Figure 7C and Supplemental Tables 25 and 26). Furthermore, following stratification according to treatment lines and treatment types, similar results were obtained regarding the predictive ability of the 2 imaging biomarkers or the combined biomarker for immunotherapy response (Supplemental Figure 17 and Supplemental Table 27).

We next compared the performance of CT imaging biomarkers and PD-L1 for predicting the immunotherapy response. We found that the combined positive score (CPS) of PD-L1 expression, a clinically approved biomarker of immunotherapy response, showed a quite modest ability in predicting immunotherapy response, with an AUC of 0.648 (95% CI, 0.567–0.729). However, the combination of LRS and MRS presented with a higher AUC of 0.727 (95% CI, 0.657–0.798) compared with CPS. Importantly, when CPS, LRS and MRS were combined into an integrative model, a significant improvement in the accuracy of immunotherapy response prediction was observed (AUC: 0.780 [0.715–0.845], P < 0.001) compared with CPS (Supplemental Figure 18).

Finally, as shown in Figure 7, D and E, Kaplan-Meier plots of progression-free survival (PFS) and OS confirmed the prognostic value of 2 radiomics imaging biomarkers (LRS and MRS) and the combined biomarker (*P* < 0.001 for all). In addition, in subgroup analyses of TNM staging and treatment lines, these biomarkers remained the statistically significant classifiers for survival prediction (Supplemental Figure 19).

## Discussion

The TIME is increasingly recognized as the key regulator of tumor progression and major determinant of all types of anticancer therapy (1–3). Tumor-infiltrating lymphoid and myeloid cells play crucial roles in the immune context (4–9). Moreover, considering the increasing application of immunotherapy in cancer, a thorough understanding of the immune status of patients is useful to evaluate the response to treatment (6, 8, 18, 19). This study initially identified that the IHC-based lymphoid and myeloid immune context was significantly associated with tumor prognosis. However, estimating the lymphoid and myeloid immune context through tissue biopsy is characterized by several disadvantages (25, 26, 30). Therefore, we constructed and validated 2 CT imaging biomarkers (LRS and MRS) for the noninvasive evaluation of the lymphoid and myeloid immune context, respectively. Radiogenomics analysis revealed that the imaging biomarkers were closely correlated with tumor-related, immune-related, and metabolism-related signaling pathways. Importantly, as a potential supplement for the IHC-based lymphoid and myeloid immune context, the imaging biomarkers showed predictive value for prognosis (DFS, PFS, and OS) and immunotherapy response. Further, based on these 2 imaging biomarkers of LRS and MRS, we developed a combined imaging classifier (LRS/MRS: low or high) with 4 radiomics immune subtypes: 1 (−/−), 2 (+/−), 3 (−/+), and 4 (+/+), which was also significantly associated with clinical outcomes and therapeutic response.

Lymphocytes and myelocytes are both major components of the immune microenvironment. These cells markedly differ in morphology, distribution, and function; hence, they are linked to varied outcomes in terms of tumor progression and diverse responses to anticancer therapies (39, 40). Previous studies have shown an association between radiomics and CD8^+^ T cells (36, 41). Nevertheless, the use of radiomics for the evaluation of the lymphoid and myeloid immune context has not been reported thus far. The present study confirmed that 2 biomarkers integrated by radiomics features from intratumoral and peritumoral areas could characterize the lymphoid and myeloid immune context. Interestingly, all imaging features (including shape, intensity, and texture) differed completely between the 2 biomarkers. These finding demonstrated the specificity and landmark of the features for recognizing the lymphoid and myeloid status. In turn, these large distinctions may be result of differences in the morphology, function, and distribution of lymphocytes and myelocytes. Moreover, based on radiogenomics data, we examined the potential relationships between the imaging biomarkers and tumor-, immune-, and metabolism-related signaling to better unscramble the imaging biomarkers.

The present study found that IHC-based lymphoid immune context was positively associated with prognosis, while IHC-based myeloid immune context was negatively associated with prognosis in GC, which was consistent with previous findings in gastrointestinal cancers and other cancers by Galon Jerome and Pitett Mikae (42, 43). Besides, an important advantage of the present study was the noninvasive qualitative characterization of lymphoid and myeloid immune context from CT images. Previous works relied on manual assessment of lymphoid and myeloid components by the pathologist from tissue samples, which was found to be invasive, time consuming, and high cost (25, 26, 28). Moreover, the histological approaches may be affected by spatial heterogeneity within a small biopsy sample. Although a recent study used radiomics analysis to estimate CD8 expression and predict immunotherapy response, the research only evaluated the association between radiomics and lymphoid component, and the radiomic signature of CD8 was only tested in a small cohort, while we validated our signature by assessing the clinical outcomes of 2,600 patients (36). To our knowledge, this is the largest study to develop a noninvasive qualitative model for lymphoid and myeloid immune context based on routine CT images, and validate its clinical relevance in the prospective and immunotherapy cohorts in GC.

The genomic and molecular differences were usually observed within different sex, age, and TNM stage of GC. Whole genome sequencing demonstrated that men had more somatic structural variants than women (44). Patients with young age and late stage presented with more aggressive neoplasms, and MSI-H was more associated with older patients with GC (44, 45). Given the difference in demographic information and clinical features among the radiogenomics cohort, immunotherapy cohort, and other cohorts in the present study, further works to explore their genomic profiles are required, and revealing genomic differences correlates with imaging appearance are important. Furthermore, due to high rates of *Helicobacter pylori* infection, high-salt diet, and other reasons, the incidence of GC was higher in East Asia, where approximately half of the total cases worldwide occurred (10). However, tumors located in the proximal third of the stomach, poor histological differentiation, and advanced disease were more common in Western regions, which could explain the survival difference between the 2 populations (10, 46, 47). Additionally, the evidence did not indicate any systematic differences in distribution of molecular subtypes, including the TCGA and ACRG subtypes, between patients from East Asian or more Western regions (48, 49). Of note, the radiogenomics cohort was based on the US population. Thus, potential population differences related to etiologies, subtypes, and molecular information of GC and their impact on radiomics performance requires further investigation. Further, the imaging appearance associated with genomic characteristics was concluded in a small radiogenomics cohort. Additional studies are warranted to fully assess these observations. In the future, we intend to validate the robustness of these findings.

Following extensive research on the TIME, several important immune subtypes or TIME subtypes based on the genome and transcriptome sequencing were identified (50, 51). To our knowledge, the radiomics immune subtype has not been reported yet. Consistent with the previously identified immune subtypes, the present study proposed a combined imaging classifier (LRS/MRS) including 4 radiomics immune subtypes: 1 (−/−), 2 (+/−), 3 (−/+), and 4 (+/+), characterized by diverse molecular features, immune infiltration, clinical outcomes, and therapies responses. The imaging subtype 2 (+/−) — characterized by an immune activation status: high infiltration of lymphoid cells and low infiltration of myeloid cells — was associated with the best prognosis and immunotherapy response, followed by the imaging subtype 4 (+/+) — characterized by an immune stabilization status: high infiltration of lymphoid cells and myeloid cell — and imaging subtype 1 (−/−) — characterized by an immune desert status: low infiltration of lymphoid cells and myeloid cells — while the prognosis and therapeutic response in the imaging subtype 3 (−/+) — characterized by an immune suppression status: low infiltration of lymphoid cells and high infiltration of myeloid cells — was the worst.

Recently, immunotherapy has led to brilliant results in various cancers, such as melanoma and non-small cell lung cancer (52, 53). However, for GC, the therapeutic outcomes are unsatisfactory, with an OR rate of only 10% to 26% among advanced disease (15, 16, 54). Although several markers, including PD-1, EBV, MSI, and TMB, have been proposed for predicting response to immunotherapy, their sensitivity and specificity are limited and warrant further investigation (16, 55–57). These facts suggest an opportunity for the identification of patients who could benefit from immunotherapy. Knowledge of the immune microenvironment is helpful in assessing the response to anticancer treatment. The imaging biomarkers proposed in this study can evaluate the immune microenvironment and predict response to immunotherapy, which may provide assistance for clinical decision making.

The current histological approaches for evaluation of immune context and therapeutic outcome require access to tissue, which might not be sufficient in patients who receive neoadjuvant therapy or have metastatic disease. Additionally, when done in a small biopsy, this approach is subject to sampling bias due to intratumor spatial heterogeneity. On the one hand, our CT biomarkers can provide additional assistances for histological diagnosis without increasing costs and damage, thus effectively enhancing the clinicians’ decision-making. On the other hand, in situations where tissue access is limited or inaccessible, the developed CT biomarkers have advantages as a potential alternative for histological examination to facilitate clinical prognostic judgments and individualized immunotherapy decision making.

However, although the predictive value of CT biomarkers for immune context and immunotherapy response is acceptable, it still cannot fully classify the immune context and treatment outcomes. In addition to the association between CT signatures and tumor immune context found in this study, it is widely accepted that H&E images and RNA-Seq also contain information of tumor microenvironment and therapeutic responses (18, 19, 58). Our team primarily focused on the advancement of machine learning and deep learning methodologies for the analysis of multimodal data, such as radiology, H&E images, and RNA-Seq. Our future objective is to seamlessly integrate this imaging signature with H&E and genomics information to effectively predict the immune context and treatment outcomes.

The primary limitation of this study is its retrospective nature. Although a prospectively collected cohort was included in this analysis, a randomized controlled trial is warranted, particularly focusing on immunotherapy. The second limitation is this study included a small number of patients who were not of Asian descent, necessitating validation by other diverse populations and ethnic groups in a large cohort. The third point is that CT images were achieved from various scanners in different institutions, which may limit the reliability of the data. A rigorously designed randomized controlled trial is urgently need.

In conclusion, this study suggests 2 radiomics imaging biomarkers (LRS and MRS) and their combined classifier with 4 imaging subtypes could accurately evaluate the IHC-derived lymphoid and myeloid immune context, which are closely correlated with clinical outcomes and immunotherapy response in GC. It may allow the optimization of individual decision making and need future prospective evidence.

## Methods

### Sex as a biological variable.

Sex as a biological variable was reported in the present study. We investigated the independent role of imaging biomarkers in male and female subgroups.

### Study design and patients.

The overall study design is shown in Figure 1. This study included 9 independent cohorts from 4 centers of 2,600 patients with GC. The training cohort (242 patients), 2 internal validation cohorts (validation cohort 1 with 160 patients and validation cohort 2 with 512 patients), and a prospective validation cohort (158 patients) were recruited from Nanfang Hospital of Southern Medical University, Guangzhou, China between 2005 and 2019. Two external validation cohorts (validation cohort 1 with 102 patients and validation cohort 2 with 1,123 patients) were recruited from Sun Yat-sen University Cancer Center, Guangzhou, China between 2008 and 2012. The radiogenomics cohort (42 patients) was acquired from The Cancer Immunome Atlas (TCIA) and The Cancer Genome Atlas database (TCGA), both constructed in the US (2022). Two anti–PD-1 immunotherapy cohorts (immunotherapy cohort 1 from Nanfang Hospital of Southern Medical University with 198 patients, and immunotherapy cohort 2 from Guangdong Provincial Hospital of Chinese Medicine with 63 patients) were retrospectively registered from June 2019 to February 2022. The inclusion and exclusion criteria are presented in the Supplemental Methods.

Clinicopathologic data was acquired from the medical record system, including age, sex, tumor location, tumor differentiation, tumor size, Lauren type, carcinoembryonic antigen (CEA), cancer antigen 19-9 (CA19-9), and tumor-nodemetastasis (TNM) staging. The TNM staging was reclassified according to the 8th edition of the AJCC Cancer Staging Manual of the American Joint Committee on Cancer (AJCC)/International Union Against Cancer. The immune checkpoint inhibitor (ICI) drugs include Nivolumab, Pembrolizumab, Sintilimab, or Toripalimab. Details on treatment regimens and patterns were listed in the Supplemental Methods. For patients without immunotherapy, DFS was defined as the time from surgery to disease progression or death due to any cause, and OS was defined as the time from surgery to death due to any cause or the last date of follow-up. For patients with immunotherapy, PFS was defined as the time from the initiation of anti–PD-1 immunotherapy to disease progression or death due to any cause, and OS was defined as the time from anti–PD-1 immunotherapy to death due to any cause or the last date of follow-up. The study adhered to NIH guidelines.

### IHC staining and quantification of the lymphoid and myeloid immune context.

IHC staining for formalin-fixed paraffin-embedded human samples was performed, as previously described (3, 22). Tumor and adjacent tissues were incubated with CD3 and CD8 antibodies to calculate the number of lymphocytes, as well as CD66b antibody to determine the number of myelocytes. Further details are provided in Supplemental Methods. The median count was selected for the qualitative analysis of these immune cells in intratumoral and peritumoral areas in the training cohort, with a score of 0 recorded when below the median or a score of 1 recorded when greater than or equal to the median. The median count was also applied to the validation cohorts. Next, for each patient, the lymphoid immune context (also termed lymphoid immune score, range: 0–4 score) and myeloid immune context (also termed myeloid immune score, range: 0–2 score) were determined by adding the qualitative scores of the corresponding immune cells in the intratumoral and peritumoral areas. Thereafter, the LIS status was divided into 2 groups: LIS low (a total score of 0–1 in the intratumor and peritumor) and LIS high (a total score of 2–4 in the intratumor and peritumor). In addition, the MIS status was divided into 2 groups: MIS low (a total score of 0 in the intratumor and peritumor) and MIS high (a total score of 1–2 in the intratumor and peritumor). The LIS and MIS from all patients were independently scored by 2 pathologists who were blinded to the clinical data. A third pathologist was consulted to reach a consensus when different opinions arose between the 2 primary pathologists. Further details are provided in the Supplemental Methods.

### CT acquisition, image processing, and features extraction.

Portal venous-phase CT images were achieved from the picture archiving and communication system (Carestream, Canada). Details on the acquisition parameters and image preprocessing are provided in the Supplemental Methods. Two radiologists with 13 and 12 years of clinical experience in the interpretation of abdominal CT images, respectively, manually delineated the primary tumor on the CT images using the ITK-SNAP software (version 3.6). Besides the center, we also created a peripheral ring surrounding the primary tumor with a thickness of 3 mm (automated dilation of the tumor boundaries by 2 mm on the outside and shrinkage of the tumor boundaries by 1 mm on the inside). This outline was used to capture information from tumor’s invasive margins. Air cavities, large vessels, and adjacent organs were excluded.

This study extracted 584 radiomics features (292 at the intratumor and 292 at the peritumor) from the region of interest on each patient’s CT imaging. The imaging features included 8 shape features, 14 first-order intensity features, and 270 second- or higher-order textural features. To evaluate the stability of radiomics features, CT images were reviewed by the aforementioned radiologists. The inter- and intra- observer reproducibility of ROI-based features were evaluated according to intra- and interclass correlation coefficients (ICCs). More detailed descriptions are given in the Supplemental Methods. The study design followed the Image Biomarker Standardization Initiative guidelines (59).

### Construction of the radiomics imaging biomarkers.

Followed the mRMR method to remove redundant features, the LASSO logistic regression and the SVM-RFE algorithms with 5-fold cross-validation were performed to select overlapping features. Next, the MLR was used to construct the radiomics imaging biomarkers. This study developed 2 CT imaging biomarkers (LRS and MRS) for evaluating the lymphoid and myeloid immune context, respectively. The optimal cut-off value for LRS and MRS was determined by the Youden’s index in the training cohort, which maximized the sum of sensitivity and specificity. Further, the LRS and MRS were integrated into a combined imaging biomarker [LRS/MRS: low (−) or high (+)] including 4 radiomics immune subtypes: 1 (−/−), 2 (+/−), 3 (−/+), and 4 (+/+). Receiver operating characteristic (ROC) curves were used to assess the ability of imaging biomarkers to distinguish lymphoid and myeloid immune context respectively, and compared using the AUC.

### SHAP interpretation and transcriptome analysis.

The Shapley additive explanations (SHAP) was performed to interpret the importance of imaging biomarkers and other clinicopathologic characteristics in prediction of the lymphoid and myeloid immune context. Moreover, gene set enrichment analysis (GSEA) was conducted to determine relationships between genomics characterization and imaging appearance.

### Association with prognosis and immunotherapy response.

We evaluated the prognostic value of 2 imaging biomarkers and their combined biomarker in all cohorts, as well as in subgroups defined by clinicopathological characteristics. Kaplan–Meier curves with the log-rank test were used to assess the DFS and OS. Univariate and multivariate Cox analyses were performed to evaluate the prognostic value of the imaging biomarkers independently from other clinicopathological characteristics. The PH assumption was checked for the Cox regression models by constructing test statistics based on Schoenfeld residual in the model construction. If the global *P* < 0.05, the PH assumption was violated; otherwise, the assumption was valid. Furthermore, the imaging biomarkers and statistically significant variables identified in the multivariate analysis were integrated into a nomogram to improve the predictive power for DFS and OS, and Harrell’s concordance index (C-index) was utilized to evaluate the discriminatory ability.

Next, we investigated the value of 2 imaging biomarkers (LRS and MRS) and their combined biomarker (4 imaging subtypes) for the prediction of anti–PD-1 immunotherapy response. The immunotherapy responses included complete response (CR), partial response (PR), stable disease (SD), or progressive disease (PD) (evaluated every 6 weeks) according to the response evaluation criteria in solid tumors (RECIST) version 1.1 (60). PD-L1 levels were represented as the combined positive score (CPS), which was defined as 100 × the number of PD-L1–stained cells (tumor, lymphocytes, and macrophages) divided by the number of tumor cells. Moreover, PFS and OS were further evaluated using the imaging biomarkers.

### Statistics.

Continuous variables were compared using 2-tailed *t* test or Mann-Whitney test. Categorical variables were compared by the κ^2^ or Fisher’s exact test. Statistical analyses were performed using the R software (version 3.5.3), SPSS statistical software (version 26.0), and Python software (version 3.6.7). 2-sided *P* values < 0.05 denoted statistically significant differences.

### Study approval.

The institutional review boards at Nanfang Hospital of Southern Medical University, Sun Yat-sen University Cancer Center, and Guangdong Provincial Hospital of Chinese Medicine, approved the analysis of anonymous data and waived the need to obtain patient informed consent. There is no requirement for informed consent for publicly available data obtained from TCIA and TCGA databases. All procedures involving human participants were conducted in accordance with the Declaration of Helsinki.

### Data availability.

All data generated in this study are included in the article or supplemental material, and are available from the corresponding authors upon further reasonable request. Values for all data points in graphs are reported in the Supporting Data Values file.

## Author contributions

ZS and YJ conceived and designed the study. YC, Z Zhou, MUA, WX, and JX conducted literature investigation. ZS and TZ acquired and analyzed the data. YX, CC, QY, and WF provided technical support. LY, KZ, Z Zhang and LQ developed the methodology. WW, JY, GL, and YJ were responsible for project administration and supervision. ZS wrote the original draft. ZS, TZ, and YJ reviewed and revised the manuscript. All authors contributed to manuscript preparation. The order of co–first authors was determined by their contributions to the study and drafting of the manuscript, and a consensus agreement was reached to ensure mutual understanding and commitment.

## Supplementary Material

Supplemental data

ICMJE disclosure forms

Supporting data values

## Figures and Tables

**Table 2:**
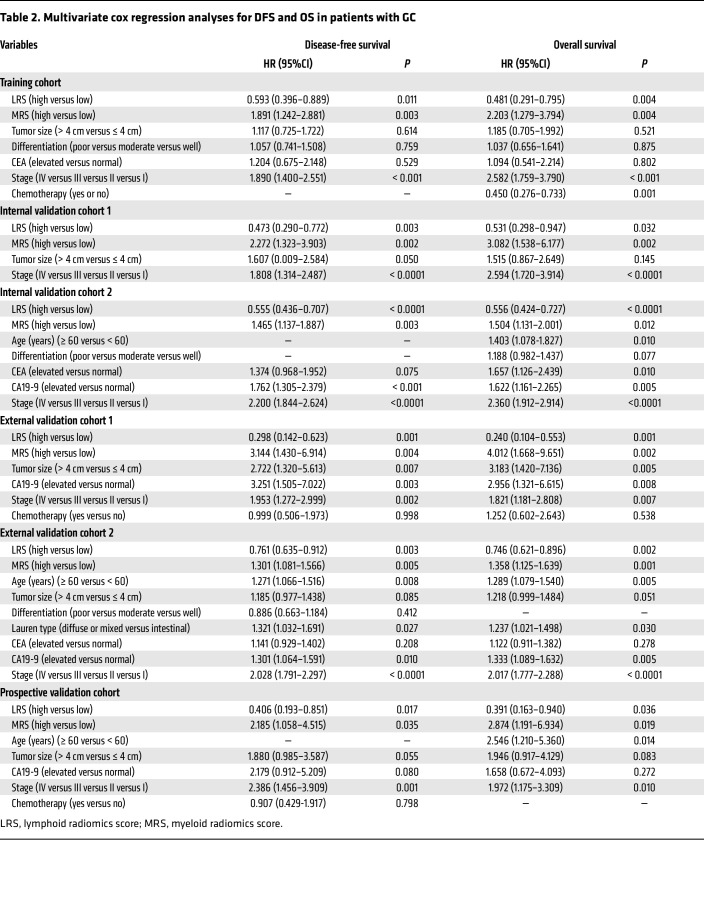
Multivariate cox regression analyses for DFS and OS in patients with GC

**Table 1:**
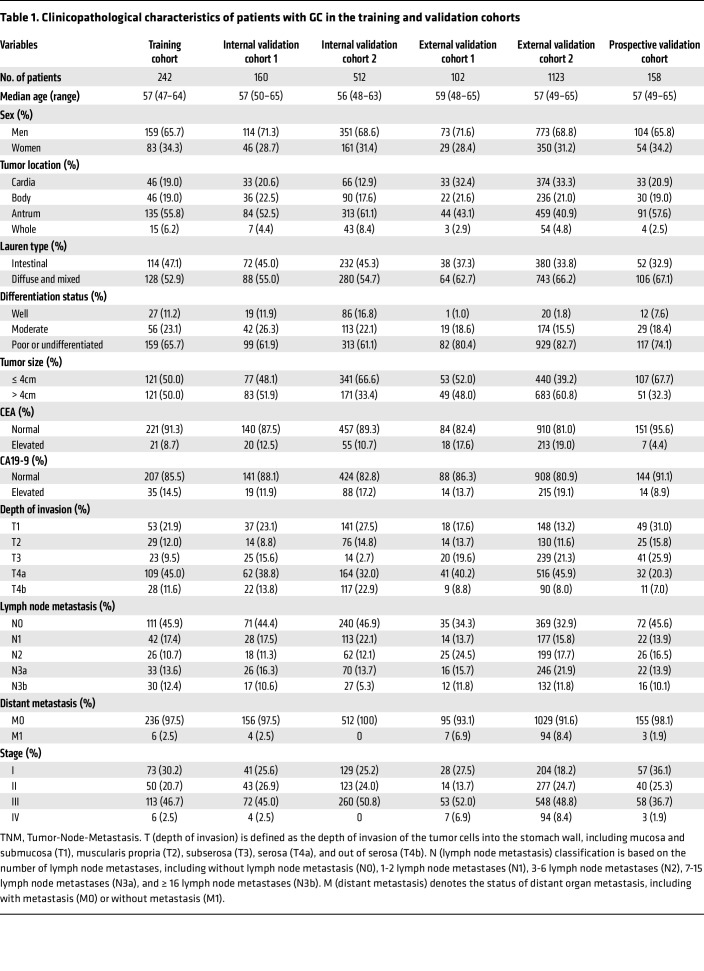
Clinicopathological characteristics of patients with GC in the training and validation cohorts

## Version 1 01/25/2024

In-Press Preview

## Version 2 03/15/2024

Electronic publication
