# Supplementary material for: Comprehensive assessment of immune context and immunotherapy response via noninvasive imaging in gastric cancer
Source: J Clin Invest. 2024 Jan 25;134(6):e175834. doi: 10.1172/JCI175834 (PMC10940098; doi:10.1172/JCI175834)
Supplement: Supplemental data [file jci-134-175834-s220.pdf]

Supplementary Materials for

Comprehensive Assessment of Immune Context and Immunotherapy Response via Noninvasive Imaging in Gastric Cancer

Zepang Sun, Taojun Zhang, M. Usman Ahmad, Zixia Zhou, Liang Qiu, Kangneng Zhou, Wenjun Xiong, Jingjing Xie, Zhicheng Zhang, Chuanli Chen, Qingyu Yuan, Yan Chen, Wanying Feng, Yikai Xu, Lequan Yu, Wei Wang, Jiang Yu, Guoxin Li, and Yuming Jiang

- **Supplementary Methods**
- **Supplementary Figures**
- **Supplementary Tables**
- **Supplementary References**

Supplementary Methods

1. Patients

The overall study design is shown in Figure 1. We retrospectively reviewed data for 2600 patients with gastric cancer in four medical centers. The patient inclusion criteria for patients without immunotherapy were: histologically confirmed gastric adenocarcinoma; standard unenhanced and contrast-enhanced abdominal CT imaging performed <30 days before surgical resection; lymphadenectomy performed and >15 lymph nodes harvested; no preoperative chemotherapy; complete information about clinicopathological characteristics; available follow-up data. We excluded those patients who had other synchronous malignant neoplasms or had received previous anticancer treatment; or if the tumor lesions could not be identified on CT images. For patients received immunotherapy, we included those with histologically confirmed gastric adenocarcinoma, standard unenhanced and contrast-enhanced abdominal CT imaging performed before immunotherapy, complete information about clinicopathological characteristics; available follow-up data. We excluded those patients who had other synchronous malignant neoplasms, or if the tumor lesions could not be identified on CT images.

2. Immunohistochemistry (IHC) staining

In the present study, lymphocytes and myelocytes at the center and invasive margin of tumor were stained and calculated. Formalin-fixed paraffin-embedded (FFPE) human samples were processed for IHC staining as previously described [1-3]. Following incubation with an antibody against human CD3 (pan T lymphocytes;

NeoMarker, clone SP7), CD8 (cytotoxic T lymphocytes; NeoMarker, clone SP16), and CD66b (myelocytes; BD Pharmingen), the paraffin sections were stained in an EnVision System (Dako). The antibody dilutions and antigen retrieval are shown in Supplementary Table 28. Every staining run contained a slide treated with phosphate buffer saline (PBS) buffer in place of the primary antibody as a negative control. Every staining run contained a slide of positive control. Prior to staining, sections were blocked with endogenous peroxidase (prepared in 1% H_2O_2/methanol solution) for 10 minutes and then microwaved for 30 minutes in 10 mM citrate buffer, pH 6.0. The sections were blocked using 10% normal rabbit serum for 30 minutes. Furthermore, all slides were stained with the same concentrations of primary antibody for each antibody and incubated with monoclonal primary antibody overnight at 4 °C, followed by incubation with an amplification system with a labeled polymer/HRP (EnVision™, DakoCytomation, Denmark) at 37°C for 30 minutes. The sections were developed with 0.05% 3, 3′-diaminobenzidine tetrahydrochloride (DAB) and counterstained with modified Harris hematoxylin. And all slides were stained with DAB dyeing for the same time for each antibody (Supplementary Table 28). Two pathologists who were blinded to clinical outcomes independently scored all samples. A third pathologist was consulted when a difference of opinion arose between the 2 primary pathologists. At low power (100), the tissue sections were screened using an inverted research microscope (model DM IRB; Leica, Germeny), and the 5 most representative fields were selected. Thereafter, to evaluate the density of stained immune cells, the 2 respective areas of invasive margin and center of tumor were measured at 200

magnification. The nucleated stained cells in each area were quantified and expressed as the number of cells per field.

We then calculated the lymphocytes (pan T lymphocytes and cytotoxic T lymphocytes) and myelocytes within the tumor and adjacent tissues. The median count was chosen for qualitative analysis of these immune cells at intratumor and peritumor in the training cohort (record 0 score when < the median or 1 score when ≥ the median), followed by application into the validation cohorts. Then, for each patient, the lymphoid immune context (also called lymphoid immune score, range: 0-4 score) and myeloid immune context (also called myeloid immune score, range: 0-2 score) were determined by adding the qualitative scores of the corresponding immune cells in the intratumor and peritumor. Next the lymphoid immune score (LIS) status was divided into two groups-LIS low (a total of 0-1 score in the intratumor and peritumor) and LIS high (a total of 2-4 score in the intratumor and peritumor). Also, the myeloid immune score (MIS) status was divided into two groups-MIS low (a total of 0 score in the intratumor and peritumor) and MIS high (a total of 1-2 score in the intratumor and peritumor).

Because IHC data was not available for patients in internal validation cohort 2, external validation cohort 2, and the prospective validation cohort, these datasets were used to validate the radiomics image biomarkers for the prognostic value, and were not used to evaluate the radiomics image biomarkers for prediction of lymphoid and myeloid immune context.

3. CT Acquisition and Image Processing

3.1 CT acquisition

All patients underwent contrast-enhanced abdominal CT scans prior to surgery or immunotherapy. All patients underwent contrast-enhanced abdominal CT using the multidetector row CT (MDCT) systems (GE Lightspeed 16, GE Healthcare Milwaukee, WI; 64-section LightSpeed VCT, GE Medical Systems, Milwaukee, WI; USA). Following intravenous contrast administration, arterial and portal venous-phase contrast-enhanced CT scans were performed after delays of 28 s and 60 s, respectively. Iodinated contrast material in the amount of 90 - 100 ml (Ultravist 370, Bayer Schering Pharma, Berlin, Germany) was injected at a rate of 3.0 or 3.5 ml/s with a pump injector (Ulrich CT Plus 150, Ulrich Medical, Ulm, Germany). The CT acquisition protocols were as follows: 120 kV; 150-190 mAs; 0.5- or 0.4-second rotation time. Contrast-enhanced CT was reconstructed with a field of view, 350×350 mm; data matrix, 512×512; in-plane spatial resolution 0.607-0.751 mm; axial slice thickness 5.0 mm for 98% patients with a range of 1.25-7.5 mm. For prospective data collection, a same CT machine and procedure (256-MDCT scanner Brilliance iCT, Philips Healthcare, Cleveland, OH, USA, Procedure sequence number: 16GSI Abdome-YAN) were selected to ensure the consistency of CT imaging, as well as in the charge of the same radiologist.

3.2 Image processing

We analyzed the portal venous-phase CT images because of well differentiation between the tumor tissue and adjacent normal bowel wall. The relatively coarse and heterogeneous resolution in z-axis compared with in-plane resolution would not allow a meaningful and reliable 3D analysis of the image. Therefore, we focused on the most

representative 2D slice, i.e., largest tumor section in the axial plane. Two radiologists C.C. and Q.Y. (with 13 and 12 years of clinical experience in abdominal CT interpretation, respectively) manually delineated the primary tumor on the CT images by using the ITK-SNAP (http://www.itksnap.org) [4, 5]. Both radiologists were present in the same room and reached visual consensus regarding tumor delineation. Both radiologists were blinded to the clinical and histopathological data but were aware that the patients had gastric cancer. All tumor contours were delineated by the two radiologists in consensus, and any discrepancy was resolved by a third radiologist (Y.X. with 33 years of experience in abdominal CT interpretation).

We extracted 584 quantitative features (292 in the intratumoral area and 292 in the peritumoral area) from the region of interest on each patient's CT imaging. The image features included 8 shape features, 14 first-order intensity features, and 270 second- and higher-order textural features. In this work, we investigated four types of texture features on the basis of gray-level co-occurrence matrices (GLCM), gray-level run length matrix (GLRLM), gray-level size zone matrix (GLSZM), and neighborhood gray-tone difference matrix wavelet decompositions (NGTDM). A Laplacian of Gaussian spatial band-pass filter (∇^2G) was used to derive image features at different spatial scales by turning the filter parameter between 1.0 and 2.5 (1.0, 1.5, 2.0, 2.5). All the image features were implemented and computed using an open-source radiomics analysis package in the MATLAB platform (https://github.com/mvallieres/radiomics). The study design followed the Image Biomarker Standardization Initiative (IBSI) guidelines, and the software used was IBSI-compliant (Supplementary Table 29).

3.3 Inter-observer and intra-observer agreements of CT image feature extraction

The inter-observer and intra-observer reproducibility were initially analyzed with 100 randomly chosen images for ROI-based texture feature extraction by two experienced radiologists (readers 1 and 2, with 13 and 12 years of clinical experience in abdominal CT study interpretation, respectively). To assess the intra-observer reproducibility, reader 1 repeated the generation of texture features twice in a 4-week period following the same procedure. The workflow for the remaining images was completed by the first radiologist.

An independent samples t-test or Kruskal-Wallis H test, where appropriate, was used to assess the differences between the features generated by reader 1 (first time) and those by reader 2 as well as between the twice-generated features by reader 1. Inter- and intra-class correlation coefficients (ICCs) were used to evaluate the intra- and inter-observer agreement of features extraction. An ICC greater than 0.75 presents good agreement.

Satisfactory inter- and intra-observer reproducibility of the texture feature extraction was achieved. There was no statistically significant difference between the features of the two readers "i.e." between reader 1's first-extracted features and those of the reader 2, with *P* values ranging from 0.53 to 0.91. The inter-observer ICCs of all metrics calculated on the basis of the "two" reader's measurements were good, ranging from 0.79 to 0.94. The intra-observer ICCs calculated based on reader 1's twice feature extraction ranged from 0.81 to 0.89. Therefore, given the relatively heavy workload,

only the first radiologist drew all the patients' CT images, and all outcomes were based on the measurement of the first reader.

4. Treatment regimens and patterns for immunotherapy cohorts

The ICIs drugs, including Nivolumab, Pembrolizumab, Sintilimab, or Toripalimab, were used in combination with chemotherapy for patients in the immunotherapy cohorts. Chemotherapy regimens involved XELOX (capecitabine plus oxaliplatin), SOX (S-1 plus oxaliplatin), FOLFOX (leucovorin, fluorouracil, and oxaliplatin), FLOT (fluorouracil, leucovorin, oxaliplatin, and docetaxel), DCF (docetaxel plus fluorouracil), and other combinations. The treatment patterns included neoadjuvant therapy, adjuvant therapy or for advanced disease. The therapeutic regimens and strategies were based on the patient's condition and preference.

5. Testing cut-off value for LRS and MRS

We compared the performance of the optimal cut-off value from Youden's index, the median, upper quartile, and lower quartile in our study. Although the determination of a cutoff for the CT biomarkers was not the aim of this study, comparative data of these cut-off values based on different methods (Youden's index, median, upper quartile, or lower quartile) are presented in the Supplementary Table 16. Firstly, the optimal cut-off value for LRS and MRS was determined by the Youden's index. This strategy maximized the sum of sensitivity and specificity, which minimized identification errors. Secondly, we found that the binary variable derived from Youden's index had a slight improvement in prognosis prediction, compared with the median, upper quartile, and lower quartile in the aspects of risk evaluation and C-index.

Thirdly, we found that the optimal cut-off value for LRS and MRS developed from Youden's index had more effective and reasonable guiding value for immunotherapy prediction. The difference in immunotherapy response was more obvious between the high and low groups derived from Youden's index, compared with that in the median, upper quartile, and lower quartile. Moreover, the highest AUC value in predicting immunotherapy response was observed in the binary variable of LRS and MRS derived from Youden's index. Finally, when combining LRS and MRS to predict the immunotherapy response, we also found that the four radiomics subtypes from the Youden's index are more valuable. Therefore, the optimal cut-off value for LRS and MRS was determined by the Youden's index, which was used for subsequent analysis.

6. Statistical analysis

Redundant features elimination was performed using the Max-Relevance and Min-Redundancy (mRMR) algorithm in "mRMRe" package; Predictive features were selected using the Least Absolute Shrinkage and Selector Operation (LASSO) logistic regression algorithm by "glmnet" package and the Support Vector Machine-Recursive Feature Elimination (SVM-RFE) algorithm by the "e1071" package with 5-fold cross-validation; ROC curves were plotted by using the "pROC" packages; Nomogram were constructed by using the "rms" package.

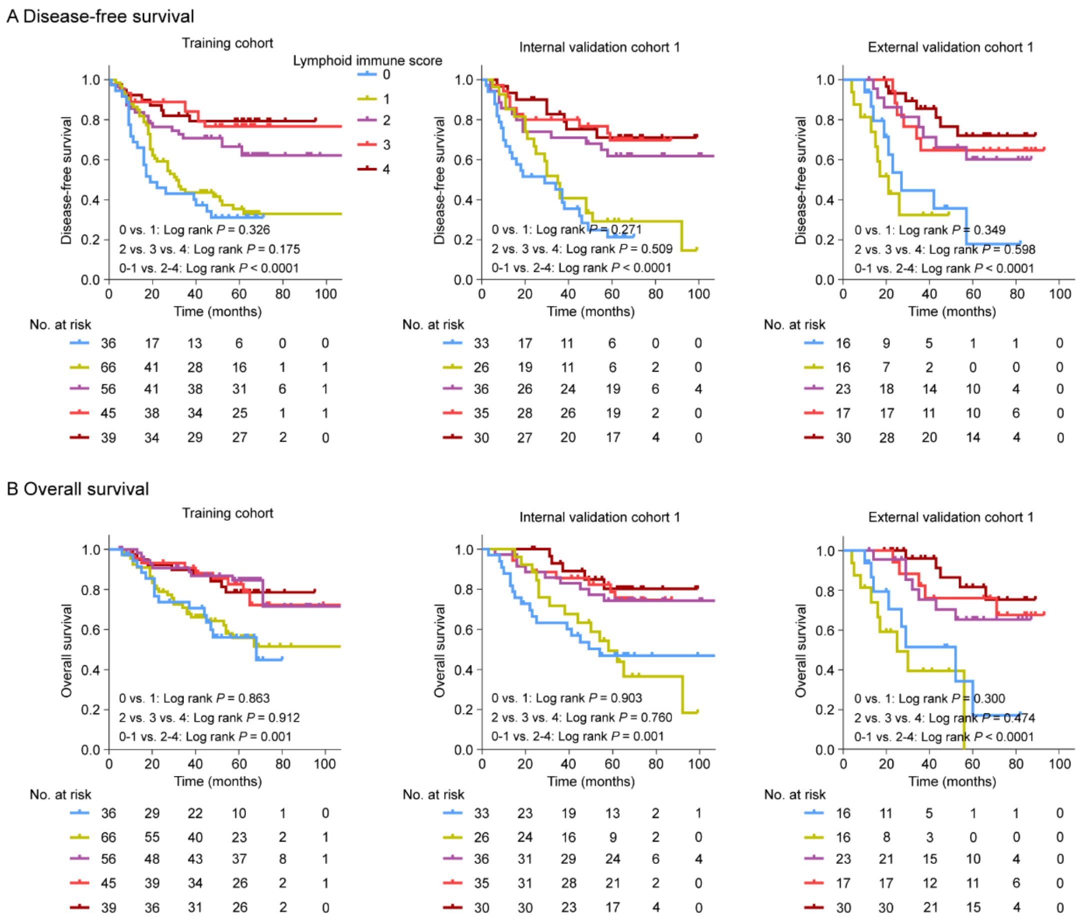

Supplementary Figure 1. Kaplan-Meier analyses of disease-free survival (DFS) and overall survival (OS) according to each score of the LIS in patients with gastric cancer. (A) Disease-free survival in the training cohort, internal validation cohort one, and external validation cohort one; (B) Overall survival in the training cohort, internal validation cohort one, and external validation cohort one. Comparisons of the above survival curves were performed with a two-sided log-rank test. LIS, lymphoid immune score.

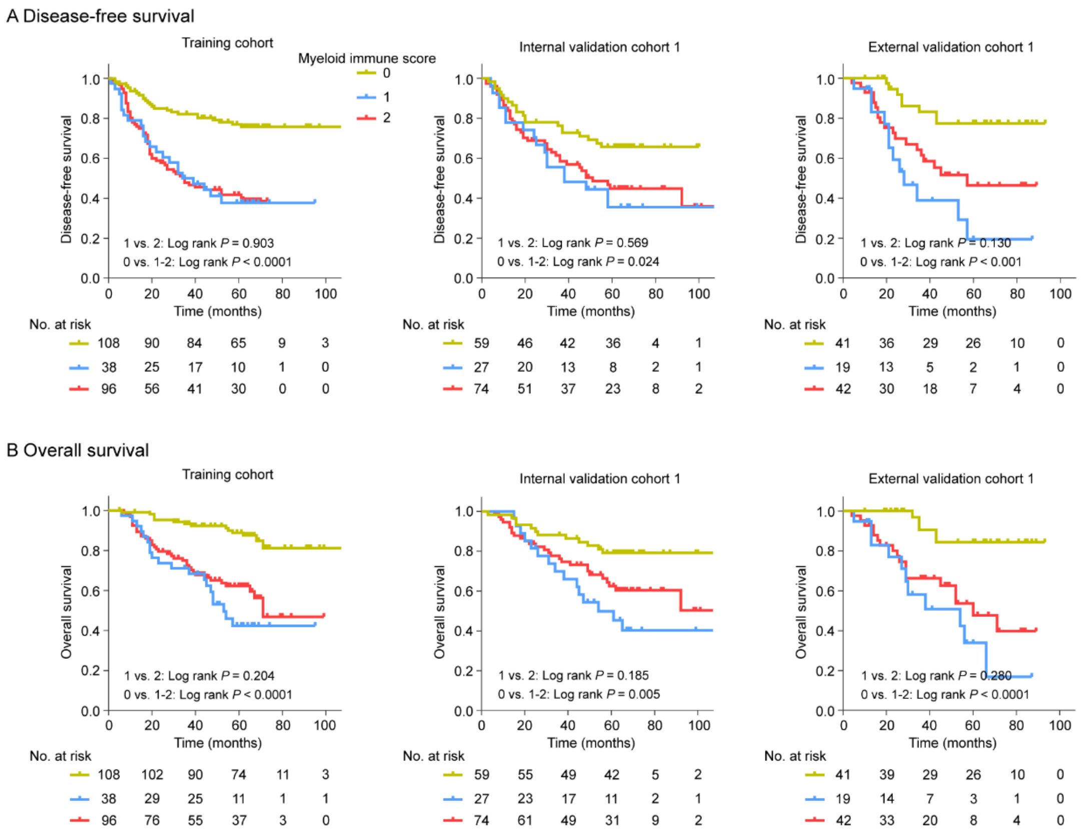

Supplementary Figure 2. Kaplan-Meier analyses of disease-free survival (DFS) and overall survival (OS) according to each score of the MIS in patients with gastric cancer. (A) Disease-free survival in the training cohort, internal validation cohort one, and external validation cohort one; (B). Overall survival in the training cohort, internal validation cohort one, and external validation cohort one. Comparisons of the above survival curves were performed with a two-sided log-rank test. MIS, myeloid immune score.

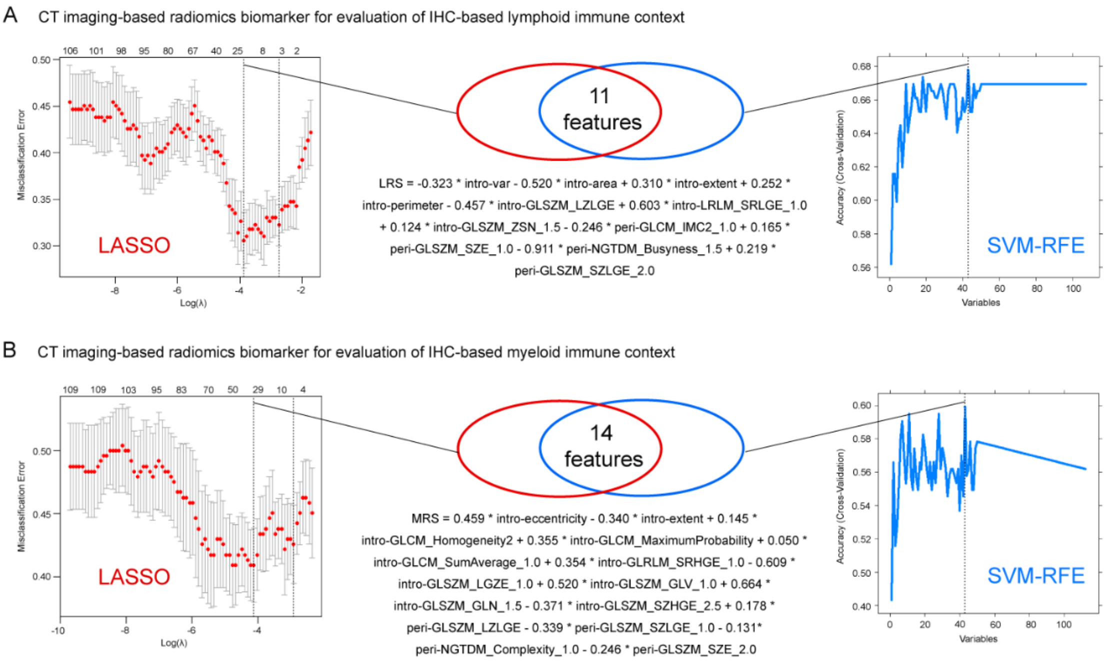

Supplementary Figure 3. Development of two radiomics imaging biomarkers (LRS and MRS) for respective evaluation of IHC-based lymphoid and myeloid immune context. (A) The overlapping features chosen by the Least Absolute Shrinkage and Selector Operation (LASSO) logistic regression and the Support Vector Machine-Recursive Feature Elimination (SVM-RFE) algorithms, and then assigned a coefficient for each radiomics features with the multivariate logistic regression (MLR) method to construct the LRS radiomics imaging biomarkers (n=242); (B) The overlapping features chosen by the LASSO logistic regression and SVM-RFE algorithms, and then assigned a coefficient for each radiomics features with the MLR method to construct the MRS radiomics imaging biomarkers (n=242). LRS, lymphoid radiomics score; MRS, myeloid radiomics score.

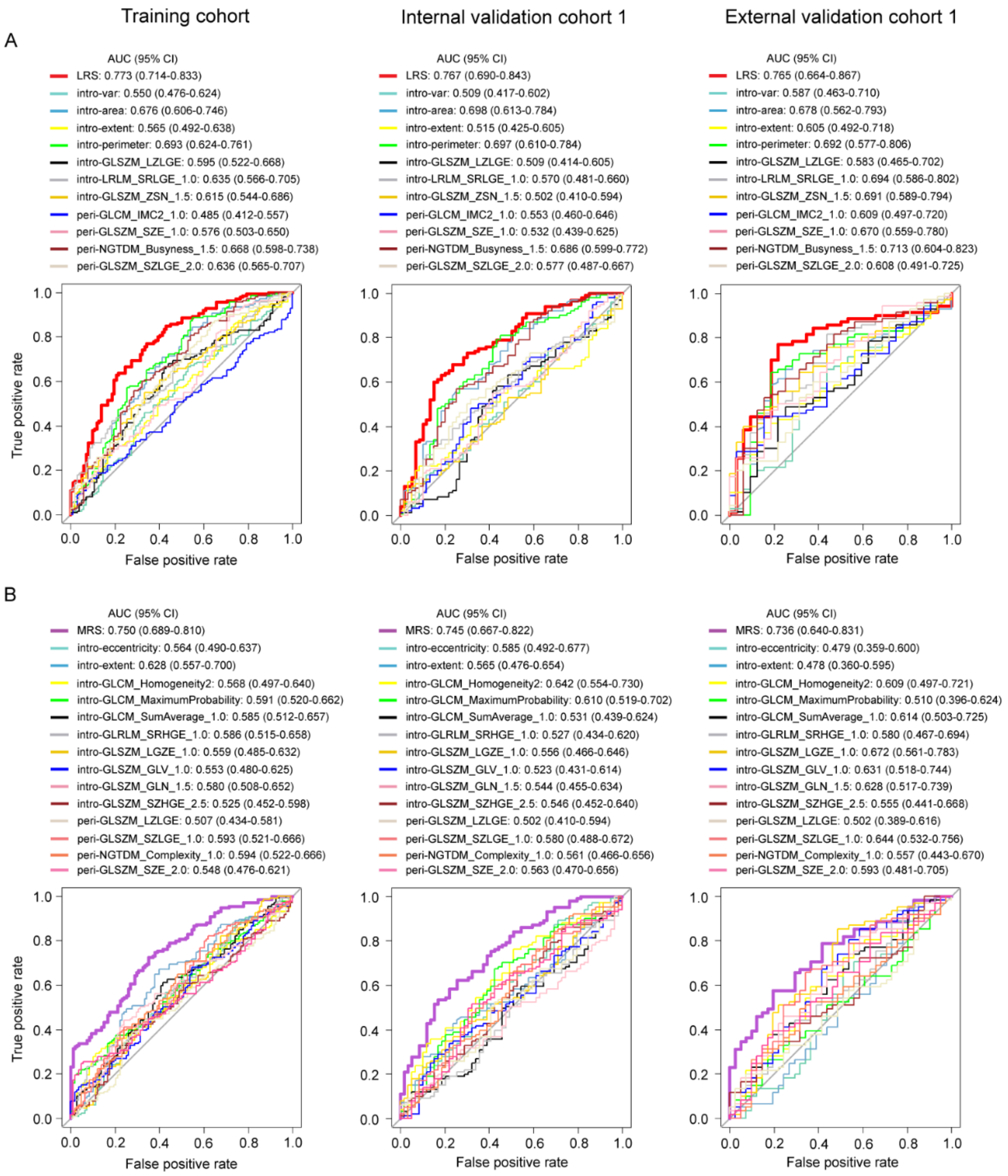

Supplementary Figure 4. ROC curves of the radiomics imaging biomarkers (LRS and MRS) and radiomics features (11 of LRS and 14 of MRS) for the lymphoid and myeloid immune context in the training cohort (n=242), internal validation cohort one (n=160), and external validation cohort one (n=102). (A) ROC curves of LRS and its 11 features for predicting the lymphoid immune context; (B) ROC curves of MRS and its 14 features for predicting the myeloid immune context. LRS, lymphoid radiomics score; MRS, myeloid radiomics score.

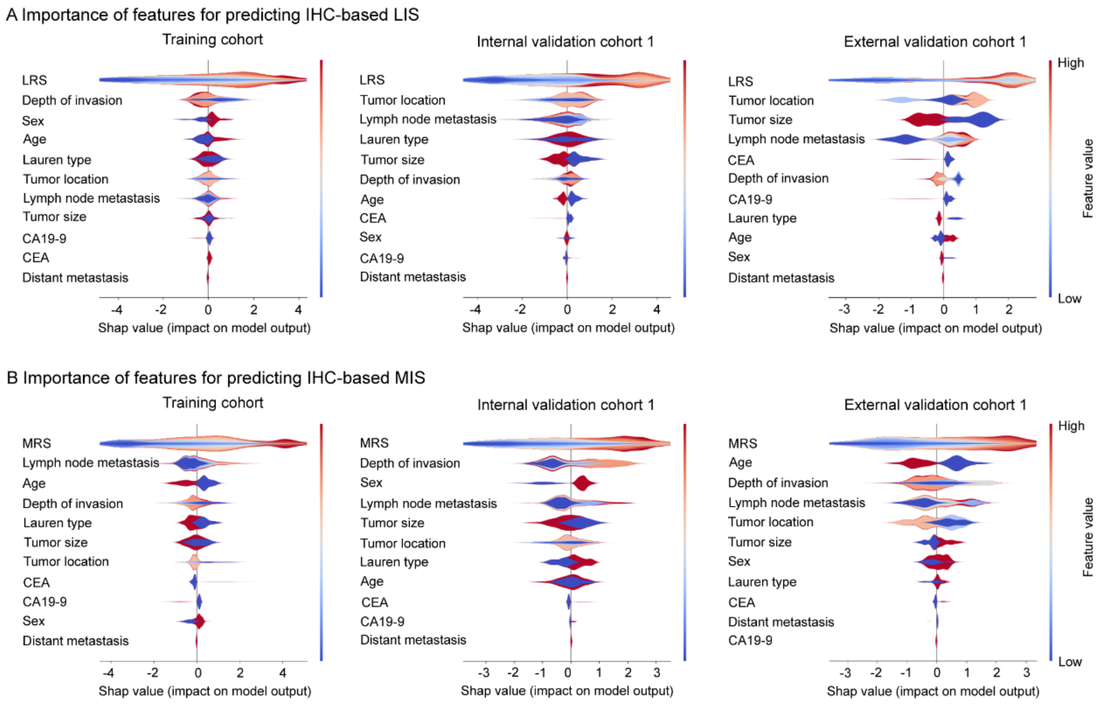

Supplementary Figure 5. The radiomics imaging biomarkers (LRS and MRS) and clinicopathologic features to predict the lymphoid and myeloid immune context in the training cohort (n=242), internal validation cohort one (n=160), and external validation cohort one (n=102) by SHAP interpretations. On the X-axis, the contribution of each feature is shown. The Shapley values is positively correlated with the importance. Moreover, a feature with a positive Shapley value will favorably impact the prediction (increase the possibility of LRS high or MRS high). The influence of the value of the feature itself is shown on the Y-axis, for example, for radiomics signature, a high value (in red) is associated with a positive Shapley value that will increase the possible of LRS high or MRS high, while a low value (in blue) will decrease the Shapley value and the possible of LRS high or MRS high. (A) SHAP plots of LRS and clinicopathologic features for predicting the lymphoid immune context; (B) SHAP plots of MRS and clinicopathologic features for predicting the myeloid immune context. LRS, lymphoid radiomics score; MRS, myeloid radiomics score.

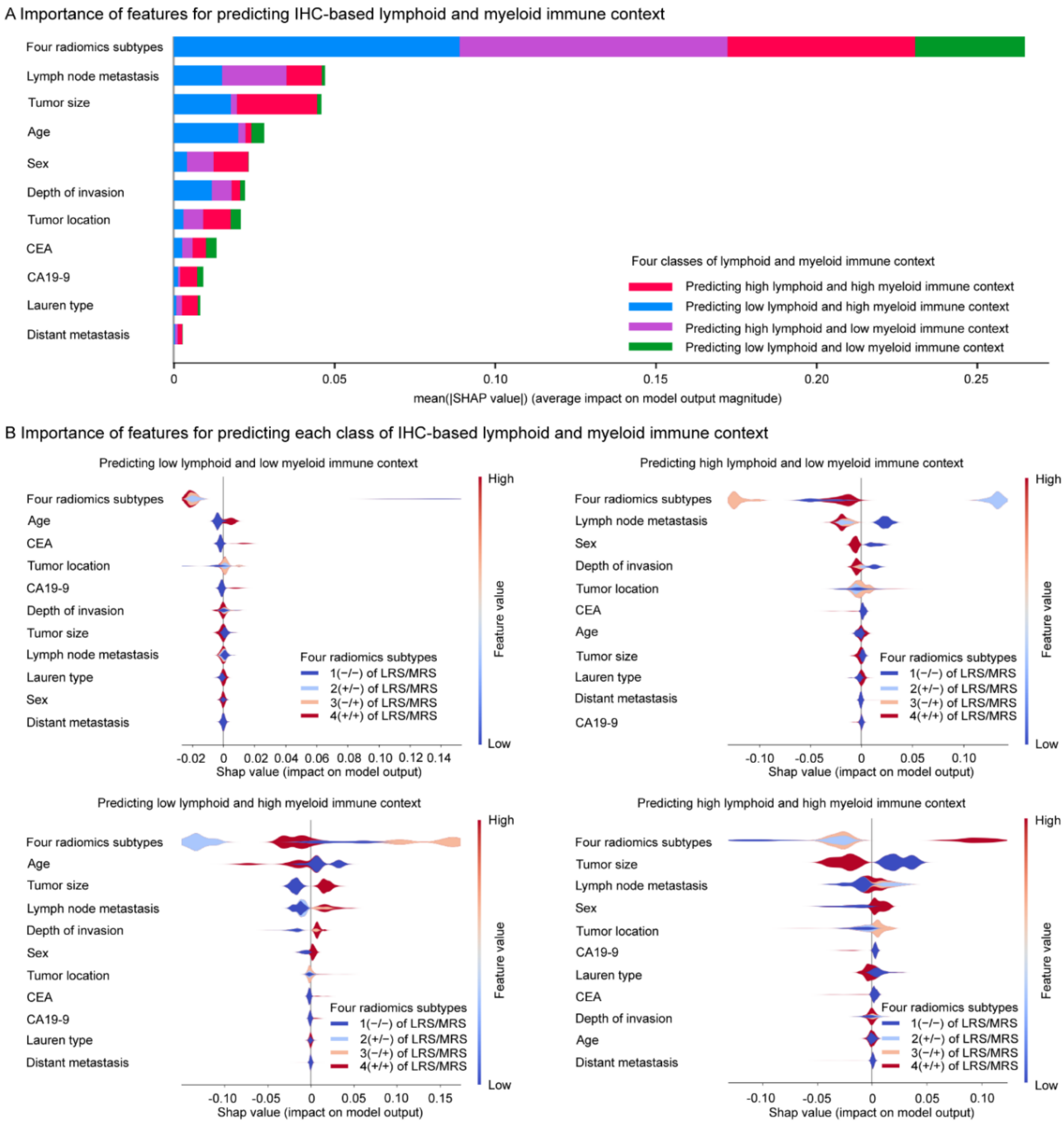

Supplementary Figure 6. The radiomics imaging subtypes [1(−/−), 2(+/−), 3(−/+), and 4(+/+)] and clinicopathologic features to predict the lymphoid and myeloid immune context by SHAP interpretations (n=504). (A) The radiomics imaging subtypes were the most important features for the prediction of the lymphoid and myeloid immune context, compared with other clinicopathological variables. (B) The imaging subtype 1(−/−) was characterized by low infiltration of lymphoid cells and myeloid cells; the imaging subtype 2(+/−) was characterized by high infiltration of lymphoid cells and low infiltration of myeloid cells; the imaging subtype 3(−/+) was characterized by low infiltration of lymphoid cells and high infiltration of myeloid cells; the imaging subtype 4(+/+) was characterized by high infiltration of lymphoid cells and myeloid cells.

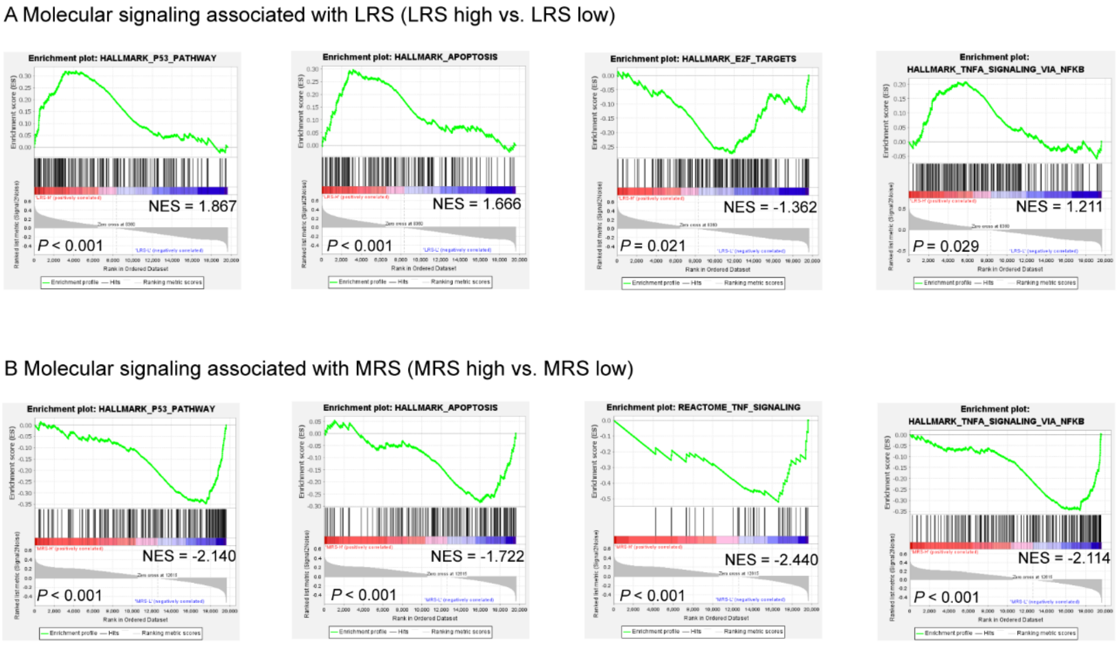

Supplementary Figure 7. GSEA and KEGG examples on the molecular signaling pathways associated with the imaging biomarkers. (A) Molecular signaling pathways associated with LRS, such as P53 pathway, apoptosis pathway, E2F targets signaling, and TNF signaling (n=42). The P value was calculated using permutation test, adjusted for multiple hypothesis testing; (B) Molecular signaling pathways associated with MRS, such as P53 pathway, apoptosis pathway, TNF signaling, and NFKB signaling (n=42). The P value was calculated using permutation test, adjusted for multiple hypothesis testing. LRS, lymphoid radiomics score; MRS, myeloid radiomics score.

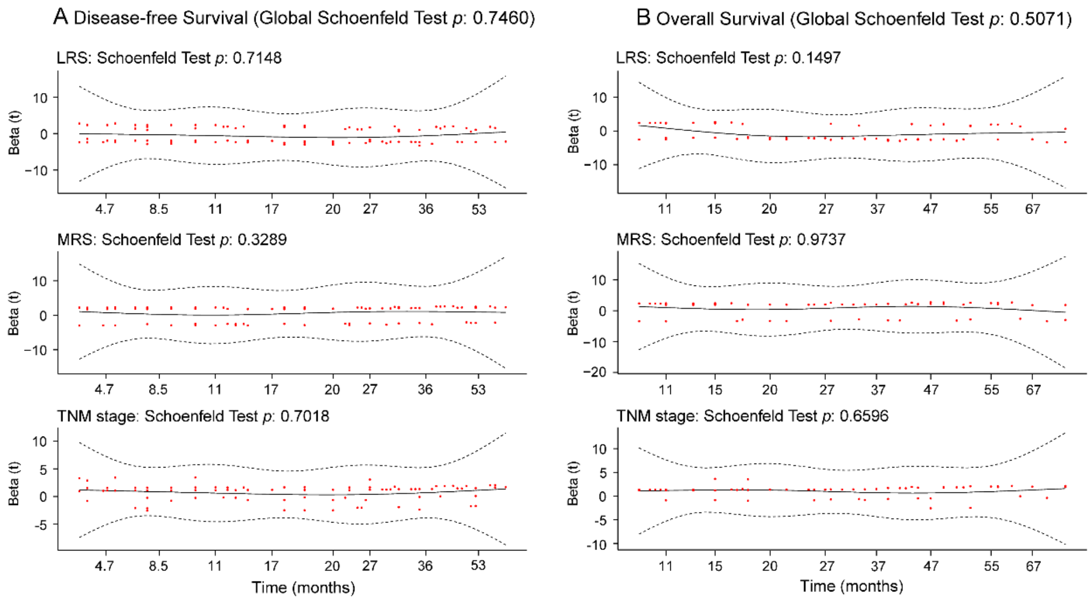

Supplementary Figure 8. The PH assumption test of the multivariate Cox regression model (n=242). (A) The PH assumption test for DFS; (B) The PH assumption test for OS. The PH assumption was checked by constructing test statistics based on Schoenfeld residual. PH, proportional hazards; LRS, lymphoid radiomics score; MRS, myeloid radiomics score.

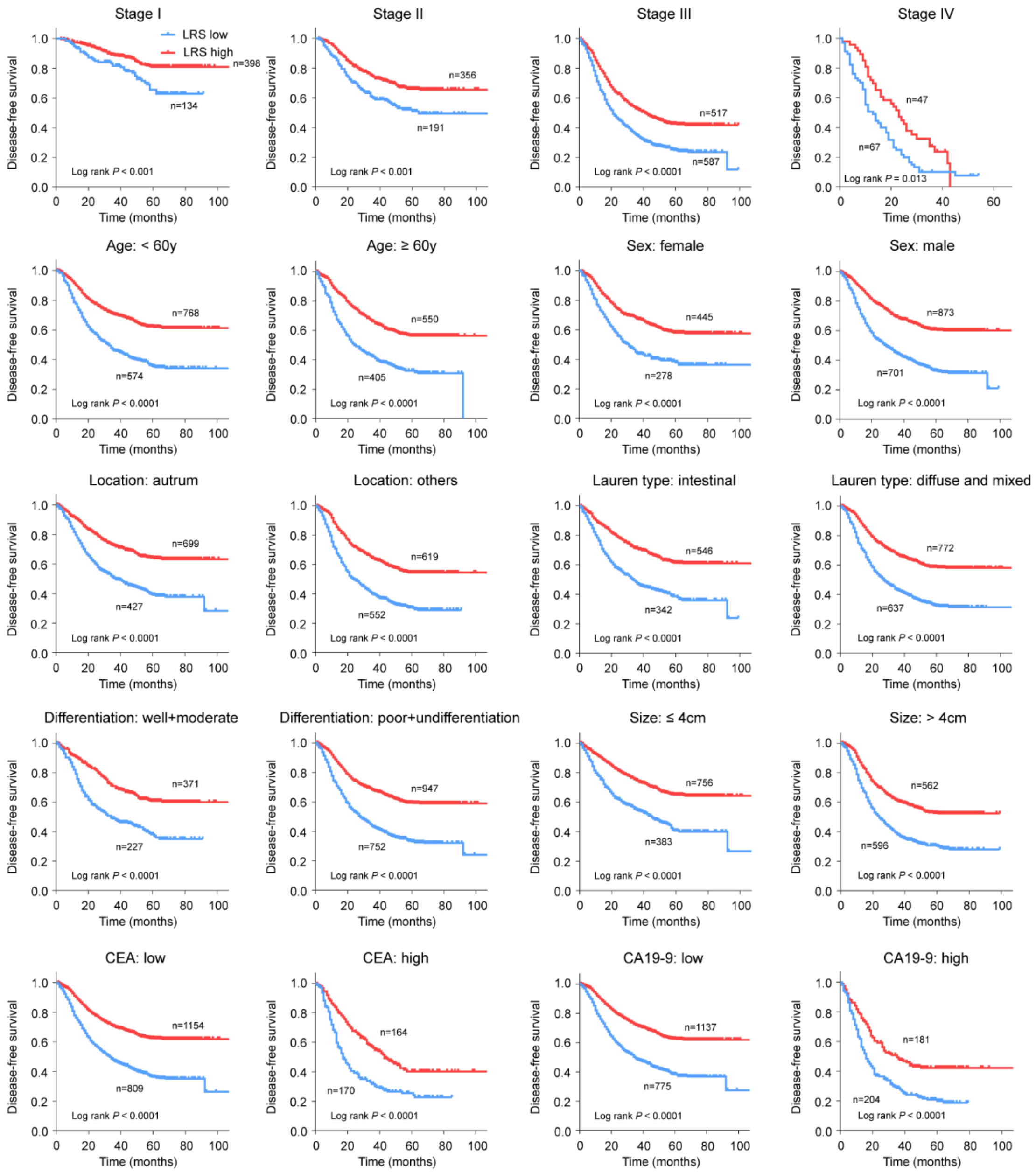

Supplementary Figure 9. Kaplan-Meier analyses of disease-free survival (DFS) according to dichotomized LRS stratified by clinicopathological risk factors in 2297 patients with gastric cancer from the training cohort, two internal validation cohort; two external validation cohort, and the prospective validation cohort. LRS, lymphoid radiomics score; *P*-values were calculated by log-rank test.

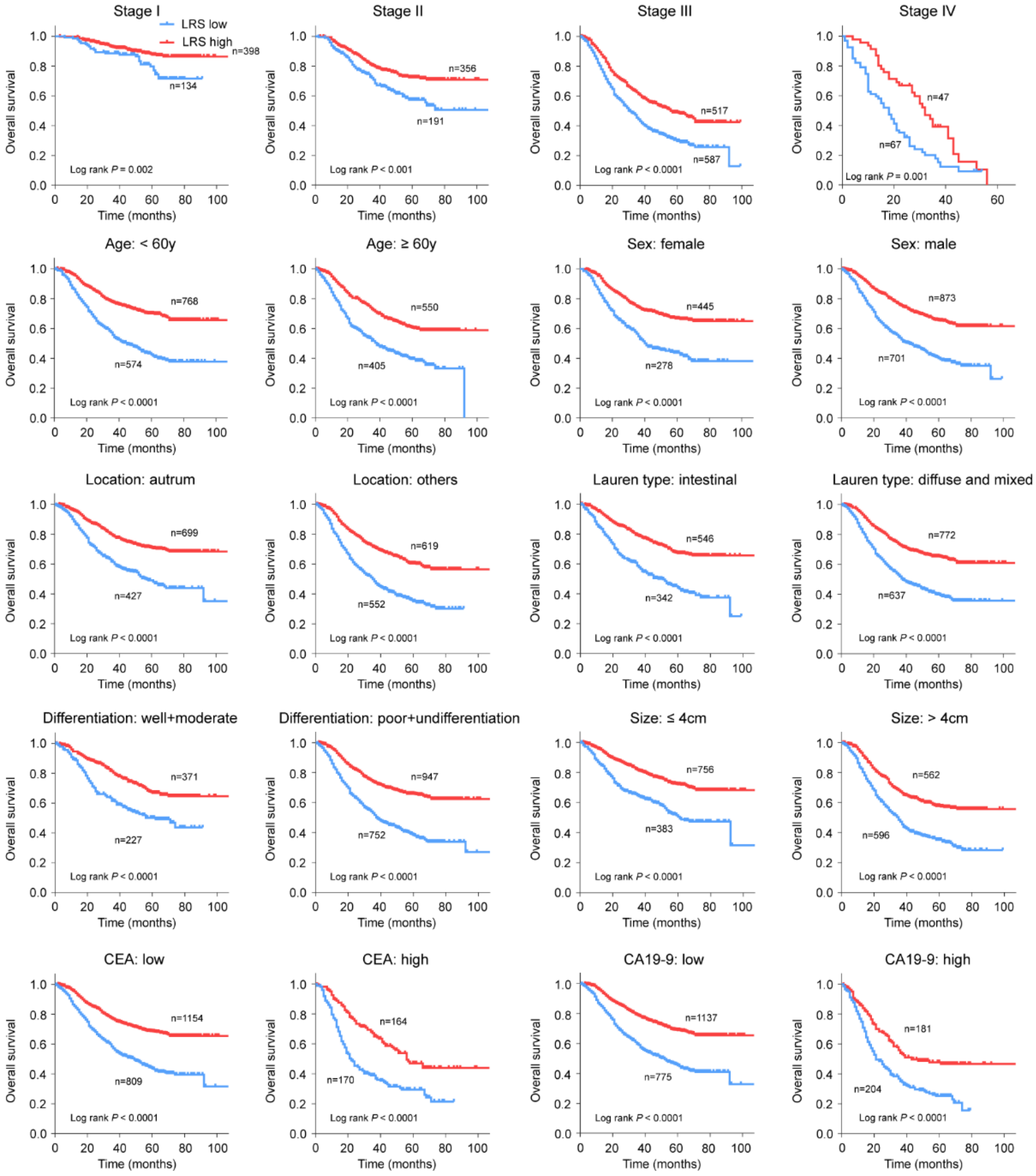

Supplementary Figure 10. Kaplan-Meier analyses of overall survival (OS) according to dichotomized LRS stratified by clinicopathological risk factors in 2297 patients with gastric cancer from the training cohort, two internal validation cohort; two external validation cohort, and the prospective validation cohort. LRS, lymphoid radiomics score; *P*-values were calculated by log-rank test.

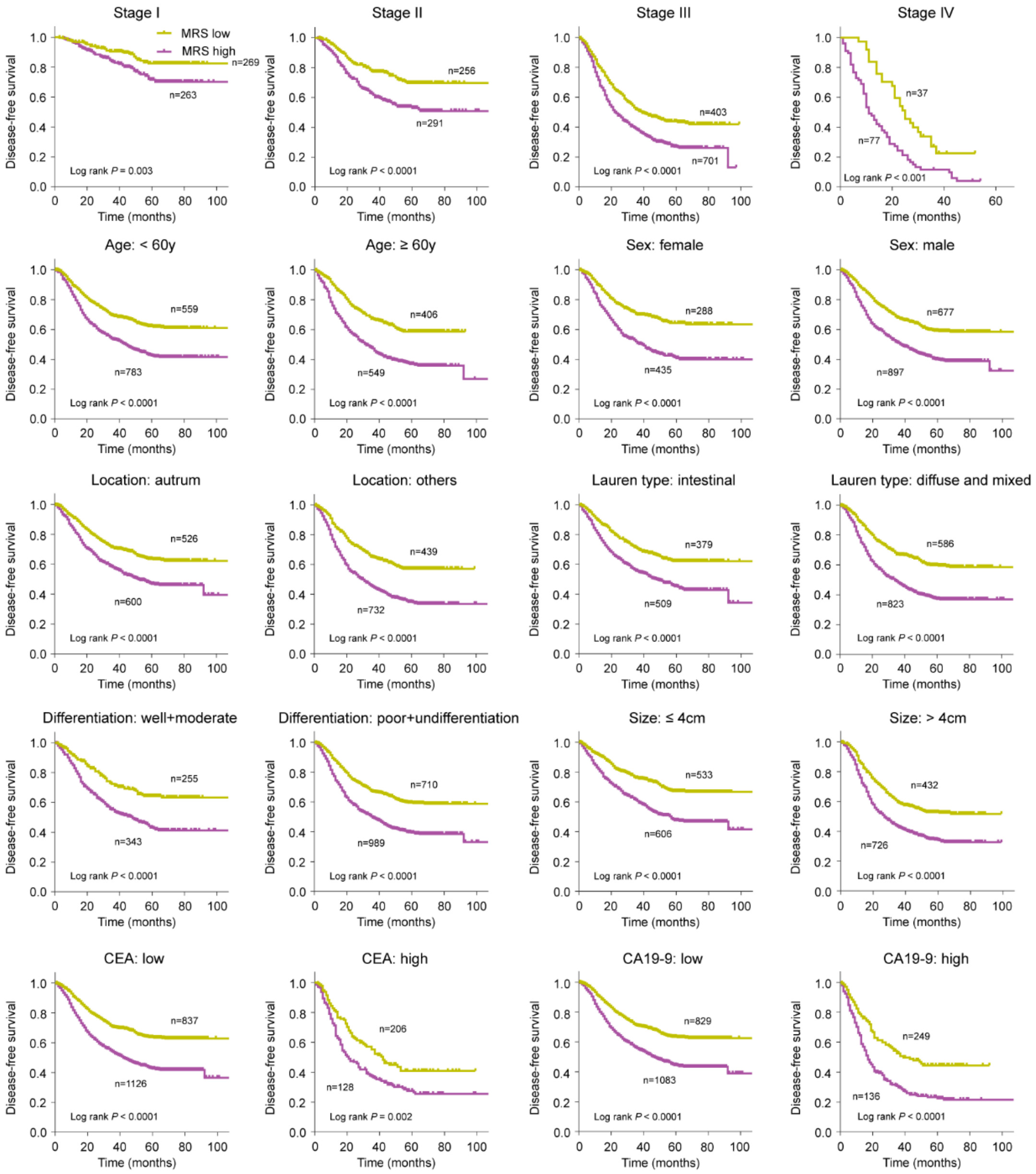

Supplementary Figure 11. Kaplan-Meier analyses of disease-free survival (DFS) according to dichotomized MRS stratified by clinicopathological risk factors in 2297 patients with gastric cancer from the training cohort, two internal validation cohort; two external validation cohort, and the prospective validation cohort. MRS, myeloid radiomics score; *P*-values were calculated by log-rank test.

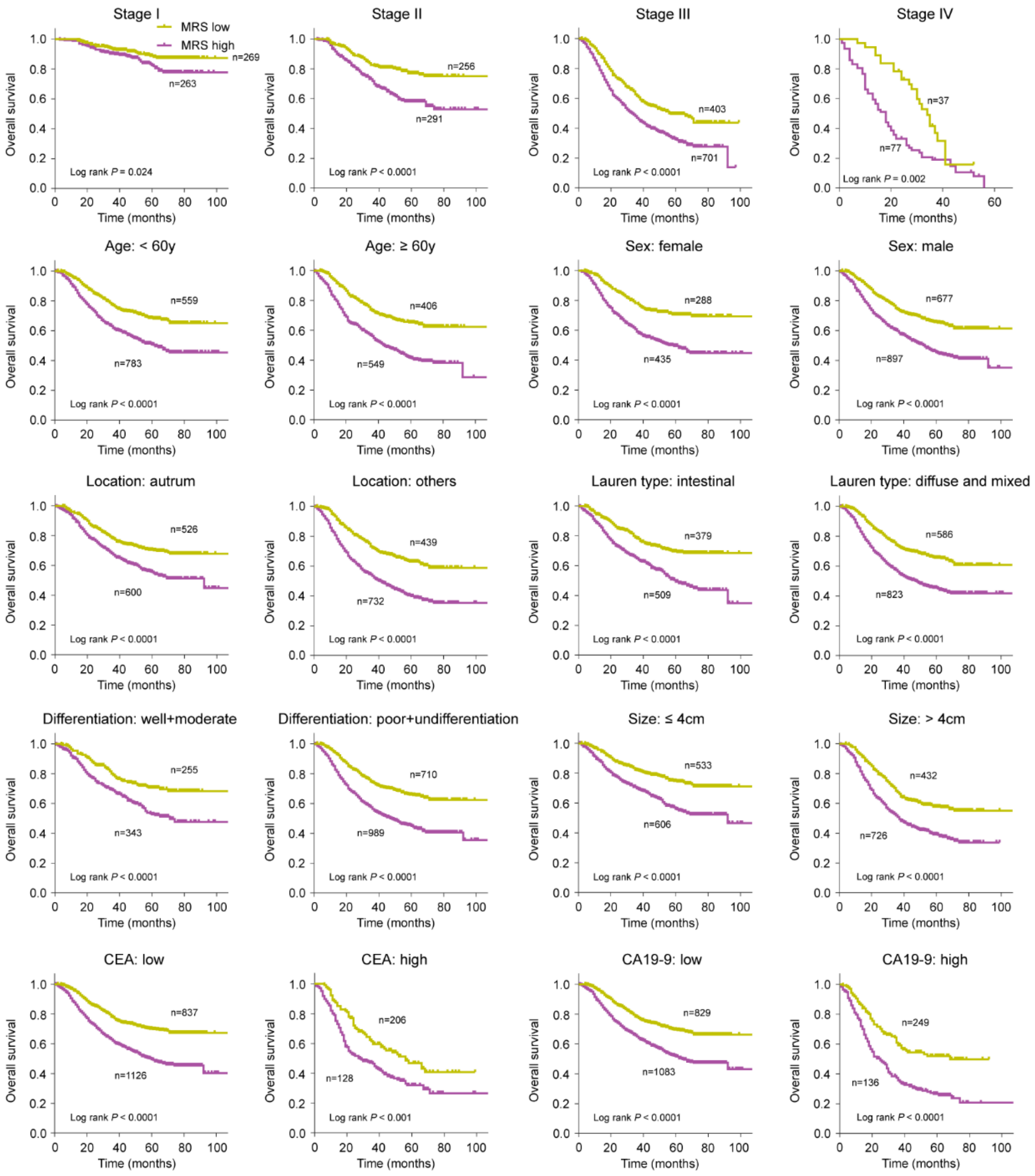

Supplementary Figure 12. Kaplan-Meier analyses of overall survival (OS) according to dichotomized MRS stratified by clinicopathological risk factors in 2297 patients with gastric cancer from the training cohort, two internal validation cohort; two external validation cohort, and the prospective validation cohort. MRS, myeloid radiomics score; *P*-values were calculated by log-rank test.

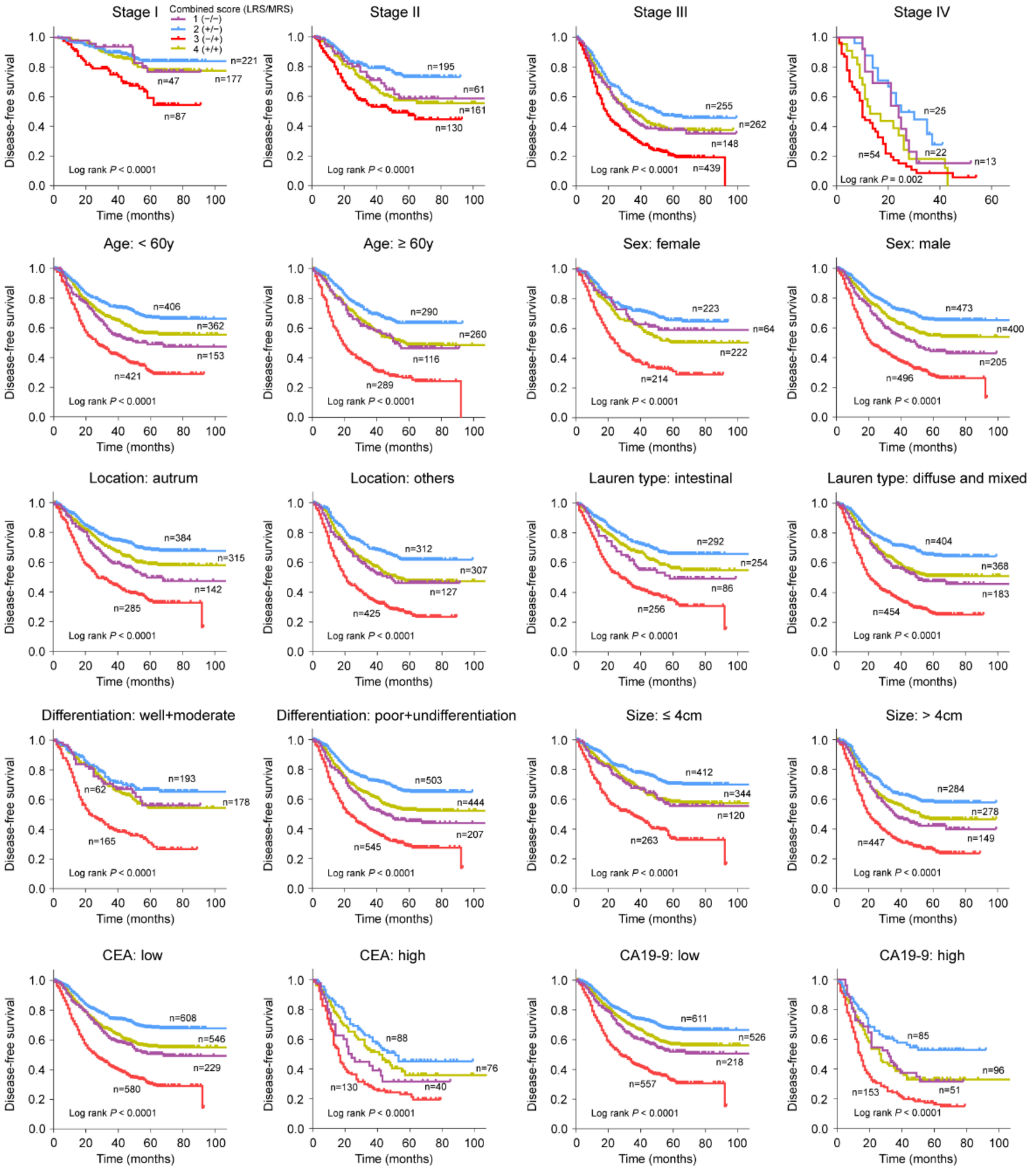

Supplementary Figure 13. Kaplan-Meier analyses of disease-free survival (DFS) according to the combined imaging biomarker (LRS/MRS) with four subtypes stratified by clinicopathological risk factors in 2297 patients with gastric cancer from the training cohort, two internal validation cohort; two external validation cohort, and the prospective validation cohort. The combined imaging biomarker (LRS/MRS): 1(-/-), 2(+/-), 3(-/+), and 4(+/+); *P*-values were calculated by log-rank test.

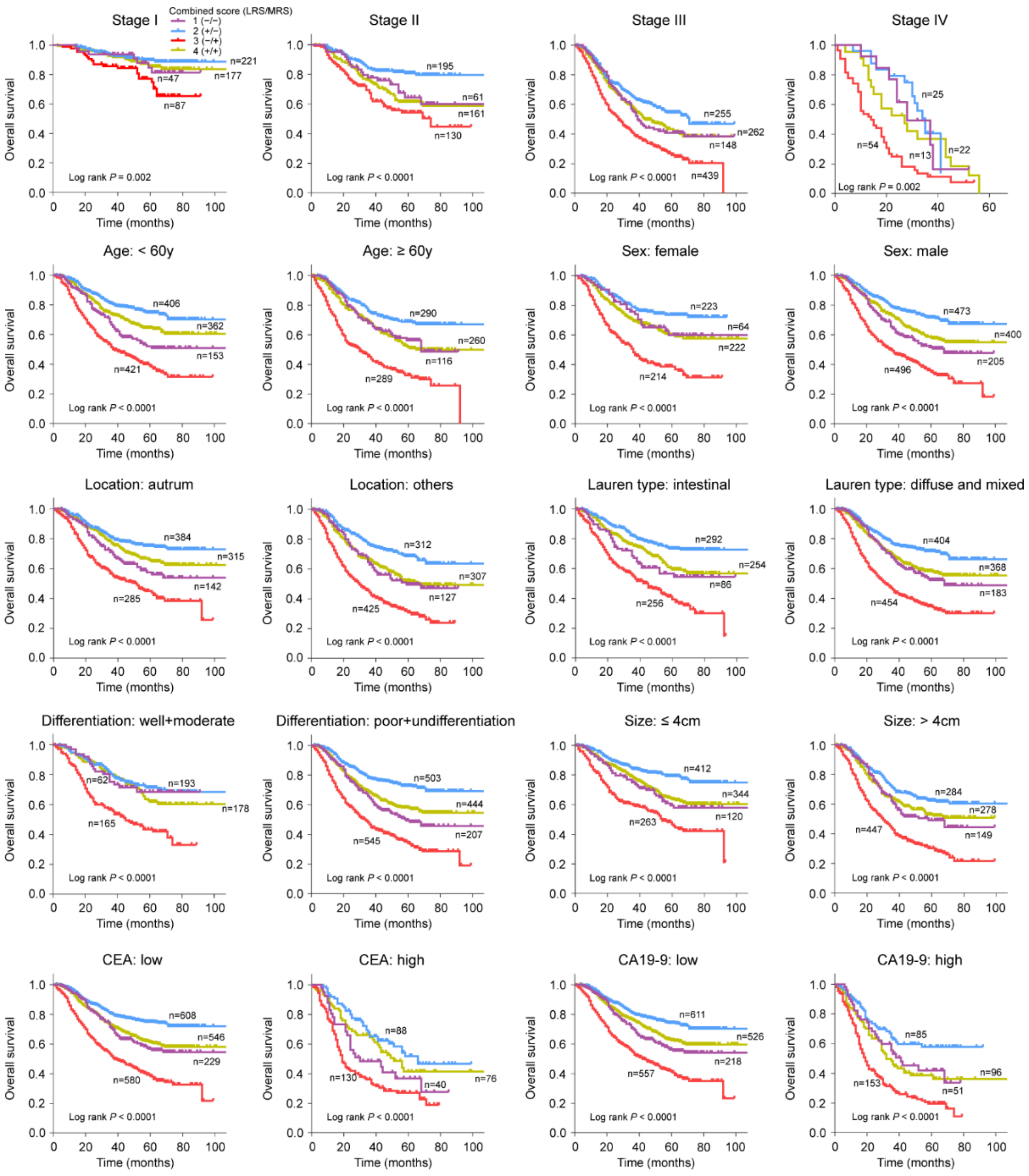

Supplementary Figure 14. Kaplan-Meier analyses of overall survival (OS) according to the combined imaging biomarker (LRS/MRS) with four subtypes stratified by clinicopathological risk factors in 2297 patients with gastric cancer from the training cohort, two internal validation cohort; two external validation cohort, and the prospective validation cohort. The combined imaging biomarker (LRS/MRS): 1(-/-), 2(+/-), 3(-/+), and 4(+/+); *P*-values were calculated by log-rank test.

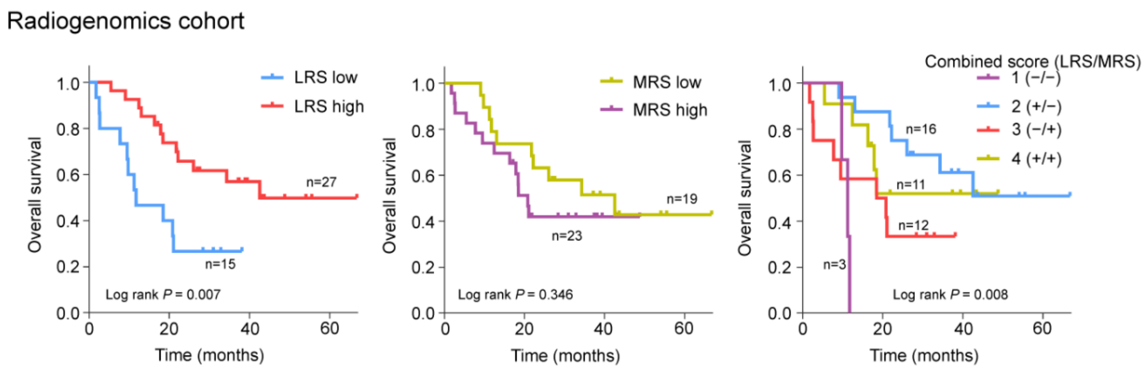

Supplementary Figure 15. Prognostic value of the radiomics imaging biomarkers for disease-free survival (DFS) and overall survival (OS) in the radiogenomics cohort. According to LRS, MRS, and the combined imaging biomarker (LRS/MRS). *P*-values were calculated by log-rank test.

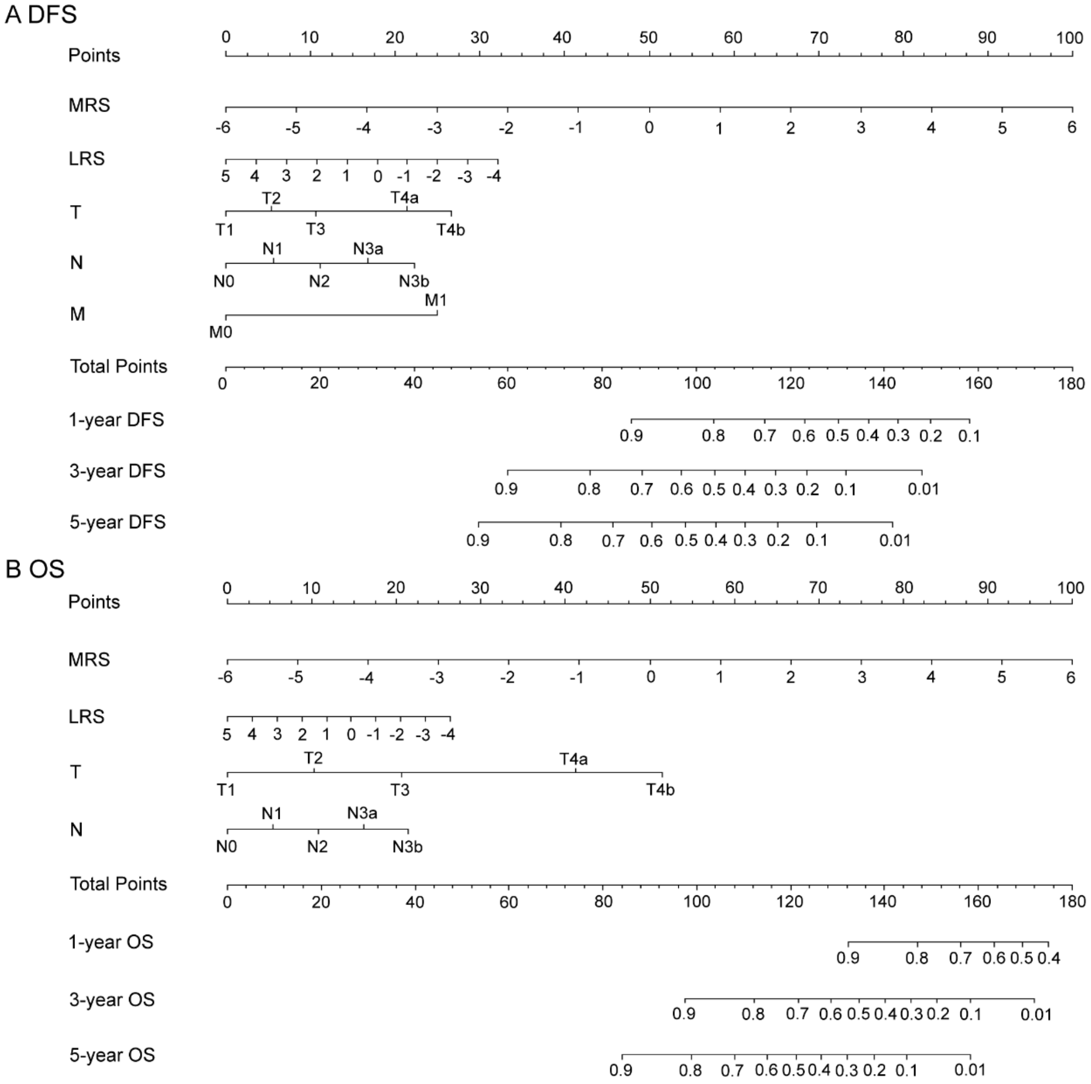

Supplementary Figure 16. Integrated nomograms to predict 1-, 3-, 5- year disease free survival (DFS) and overall survival (OS) for patients with gastric cancer (n=242). To determine how many points toward the probability of DFS and OS the patient receives for his or her LRS or MRS, locate the patient's LRS or MRS on their axis, draw a line straight upward to the point axis, repeat this process for each variable, sum the points achieved for each of the risk factors, locate the final sum on the Total Point axis, and draw a line straight down to find the patient's probability of DFS and OS. LRS, lymphoid radiomics score; MRS, myeloid radiomics score.

A First line treatment

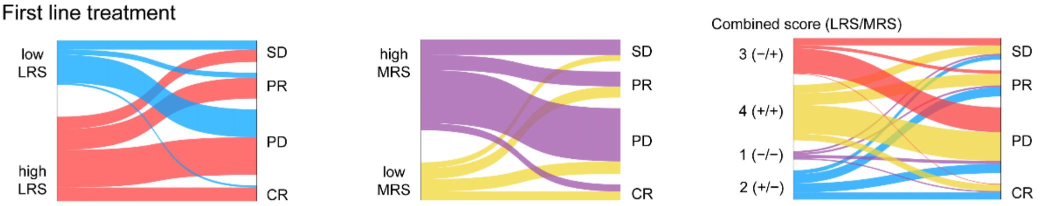

Immunotherapy response of radiomics subtypes in the first line anti-PD-1 treatment.

Response	LRS		*P*	MRS		*P*	Combined score (LRS/MRS)				*P*
	Low	High	<0.001	Low	High	0.024	1 (−/−)	2 (+/−)	3 (−/+)	4 (+/+)	0.003
CR (%)	4.7%	20.5%		20.4%	10.2%		13.3%	23.5%	2.0%	18.4%	
PR (%)	15.6%	33.7%		34.7%	21.4%		26.7%	38.2%	12.2%	30.6%	
SD (%)	29.7%	16.9%		22.4%	22.4%		33.3%	17.6%	28.6%	16.3%	
PD (%)	50.0%	28.9%		22.4%	45.9%		26.7%	20.6%	57.1%	34.7%	

B Second and third line treatment

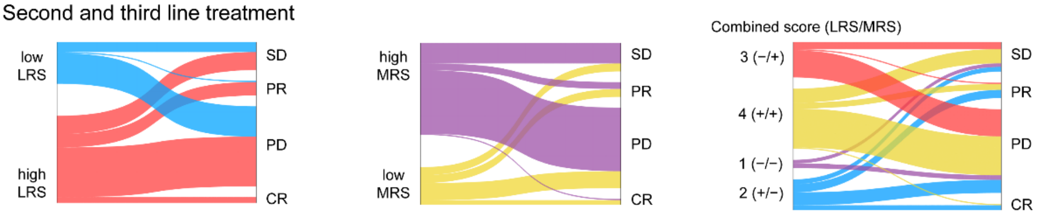

Immunotherapy response of radiomics subtypes in the second and third line anti-PD-1 treatment.

Response	LRS		*P*	MRS		*P*	Combined score (LRS/MRS)				*P*
	Low	High	0.066	Low	High	0.006	1 (−/−)	2 (+/−)	3 (−/+)	4 (+/+)	0.008
CR (%)	0.0%	6.5%		12.1%	1.2%		0.0%	15.4%	0.0%	2.0%	
PR (%)	2.7%	15.6%		21.2%	7.4%		0.0%	26.9%	3.3%	9.8%	
SD (%)	24.3%	20.8%		21.2%	22.2%		42.9%	15.4%	20.0%	23.5%	
PD (%)	73.0%	57.1%		45.5%	69.1%		57.1%	42.3%	76.7%	64.7%	

Supplementary Figure 17. Predictive value of the radiomics imaging biomarkers for anti-PD-1 immunotherapy response according to the treatment line. (A) The ratio of different immunotherapy responses from two radiomics imaging biomarkers (LRS and MRS) and their combined biomarker (LRS/MRS) in the first line treatment (n=147). Data was compared by the chi-squared test; (B) The ratio of different immunotherapy responses from two radiomics imaging biomarkers (LRS and MRS) and their combined biomarker (LRS/MRS) in the second-third line treatment (n=114). Data was compared by the chi-squared test. LRS, lymphoid radiomics score; MRS, myeloid radiomics score.

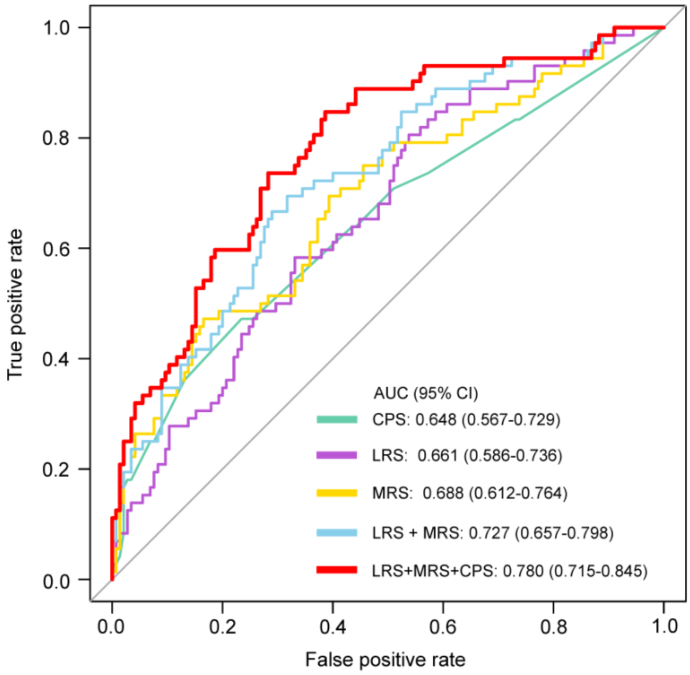

Supplementary Figure 18. Receiver operator characteristic (ROC) curves of the CT imaging biomarkers (LRS and MRS), CPS and the integrated model combining CT imaging biomarkers and CPS for predicting immunotherapy response. A significant improvement in the accuracy of immunotherapy response prediction was observed in the integrated model (available for n=217, $P < 0.001$). P value was calculated by Delong test. CPS, combined positive score; LRS, lymphoid radiomics score; MRS, myeloid radiomics score.

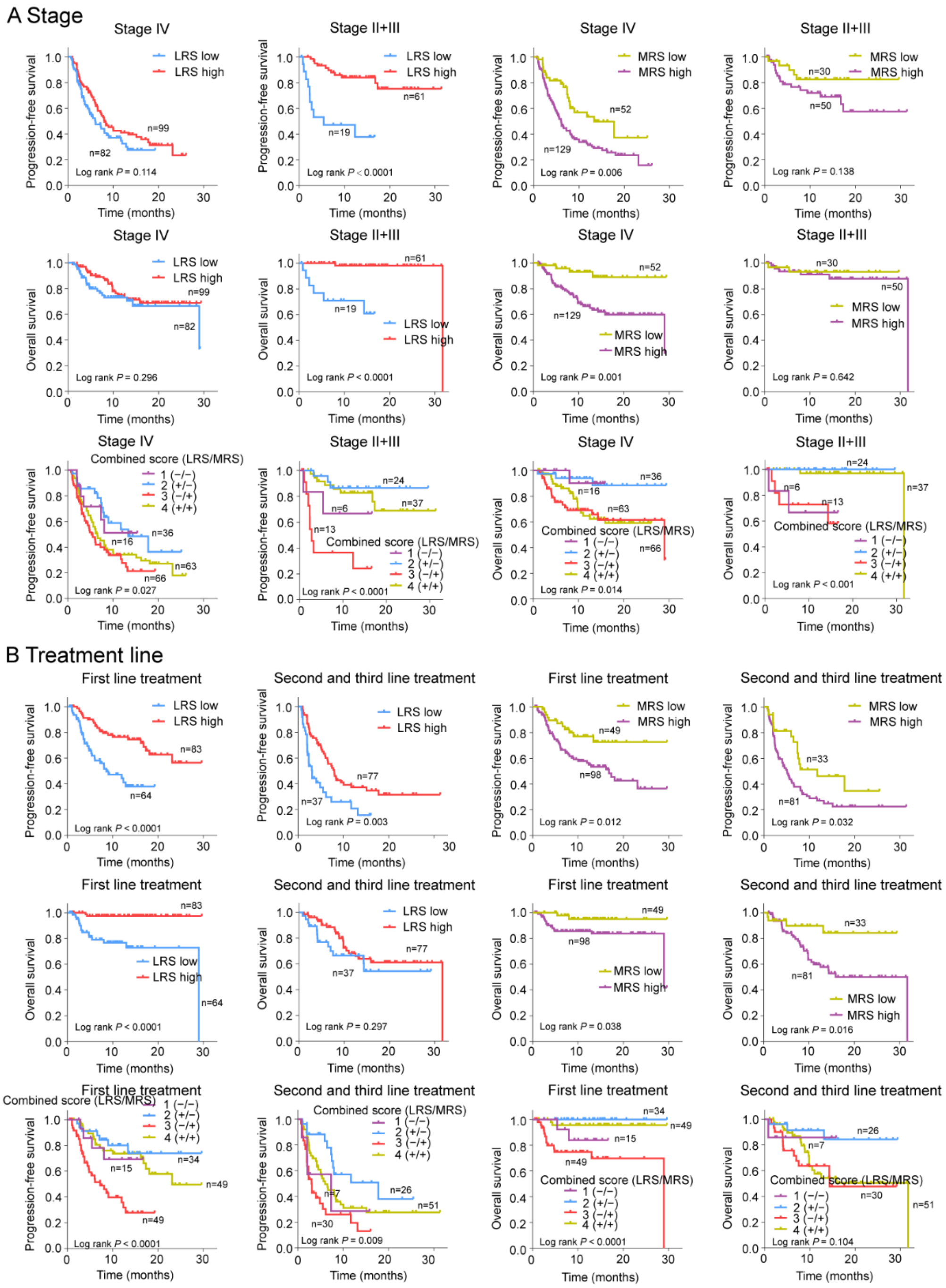

Supplementary Figure 19. Kaplan-Meier analyses of progression-free survival (PFS) and overall survival (OS) according to two radiomics imaging biomarkers (LRS and MRS) and the combined imaging biomarker (LRS/MRS) with four subtypes stratified by clinicopathological risk factors in GC patients treated with anti-PD-1 immunotherapy. (A) Stratified by stage; (B) Stratified by treatment line. Comparisons of the above survival curves were performed with a two-sided log-rank test. LRS, lymphoid radiomics score; MRS, myeloid radiomics score.

Supplementary Table 1. Characteristics of patients with gastric cancer in two anti-PD-1 immunotherapy cohorts and the radiogenomics cohort.

Variables	Immunotherapy cohort 1, SMU cohort, n=198		Immunotherapy cohort 2, GPHCM cohort, n=63		Radiogenomics cohort, n=42	
	**n**	**%**	**n**	**%**	**n**	**%**
**Median age (range)**	55.0 (47.0-65.0)		60 (48-66)		67.0 (59.0-70.0)	
**Sex**						
Female	83	41.9	29	46.0	6	14.3
Male	115	58.1	34	54.0	36	85.7
**Stage**						
I	0	0	0	0	1	2.4
II	17	8.6	0	0	7	16.7
III	47	23.7	16	25.4	31	73.8
IV	134	67.7	47	74.6	3	7.1
**Immunotherapy response**						
CR	24	12.1	1	1.6	-	-
PR	40	20.2	11	17.5	-	-
SD	33	16.7	25	39.7	-	-
PD	101	51.0	26	41.3	-	-
**Treatment line**					-	-
First line	114	57.6	33	52.4	-	-
Second line	48	24.2	24	38.1	-	-
Third line	36	18.2	6	9.5	-	-
**Treatment type**						
Neoadjuvant therapy	37	18.7	9	14.3	-	-
Adjuvant therapy	27	13.6	7	11.1	-	-
For advanced disease	134	67.7	47	74.6	-	-

CR: complete response, PR: partial response, SD: stable disease, PD: progressive disease.

Supplementary Table 2. Clinical characteristics of patients according to the LIS in the training, internal, and external validation cohorts.

Variables	Training cohort, n = 242			Internal validation cohort 1, n = 160			External validation cohort 1, n = 102		
	low LIS	high LIS	*P*	low LIS	high LIS	*P*	low LIS	high LIS	*P*
**Median age (range)**	58 (46-63)	56 (48-64)	0.643	59 (50-65)	57 (49-65)	0.824	55 (42-63)	59 (50-66)	0.177
**Male (%)**	67 (65.7%)	92 (65.7%)	0.552	45 (75.0%)	69 (69.0%)	0.417	26 (81.3%)	47 (67.1%)	0.143
**Tumor size (%)**			0.037			0.025			0.005
≤4cm	43 (42.2%)	78 (55.7%)		22 (36.7%)	55 (55.0 %)		10 (31.3%)	43 (61.4%)	
>4cm	59 (57.8%)	62 (44.3%)		38 (63.3%)	45 (45.0%)		22 (68.8%)	27 (38.6%)	
**Tumor location (%)**			0.765			0.584			0.140
Cardia	18 (17.6%)	28 (20.0%)		14 (23.3%)	19 (19.0%)		12 (37.5%)	21 (30.0%)	
Body	18 (17.6%)	28 (20.0%)		16 (26.7%)	20 (20.0%)		9 (28.1%)	13 (18.6%)	
Antrum	58 (56.9%)	81 (55.0%)		28 (46.7%)	56 (56.0%)		9 (28.1%)	35 (50.0%)	
Whole	8 (7.8%)	6 (5.0%)		2 (3.3%)	5 (5.0%)		2 (6.3%)	1 (1.4%)	
**Differentiation status (%)**			0.277			0.218			0.687
Well	8 (7.8%)	19 (13.6%)		5 (8.3%)	14 (14.0%)		0 (0.0%)	1 (1.4%)	
Moderate	22 (21.6%)	34 (24.3%)		20 (33.3%)	22 (22.0%)		7 (21.9%)	12 (17.1%)	
Poor and undifferentiated	72 (70.6%)	87 (62.1%)		35 (58.3%)	64 (64.0%)		25 (78.1%)	57 (81.4%)	
**Lauren type (%)**			0.291			0.101			0.396
Intestinal type	44 (43.1%)	70 (50.0%)		22 (36.7%)	50 (50.0%)		10 (31.3%)	28 (40.0%)	
Diffuse or mixed type	58 (56.9%)	70 (50.0%)		38 (63.3%)	50 (50.0%)		22 (68.8%)	42 (60.0%)	
**CEA (%)**			0.320			0.459			0.188
Normal	91 (89.2%)	130 (92.9%)		51 (85.0%)	89 (89.0%)		24 (75.0%)	60 (85.7%)	
Elevated	11 (10.8%)	10 (7.1%)		9 (15.0%)	11 (11.0%)		8 (25.0%)	10 (14.3%)	
**CA19-9 (%)**			0.229			0.147			0.106
Normal	84 (82.4%)	123 (87.9%)		50 (83.3%)	91 (91.0%)		25 (78.1%)	63 (90.0%)	
Elevated	18 (17.6%)	17 (12.1%)		10 (16.7%)	9 (9.0%)		7 (21.9%)	7 (10.0%)	
**Depth of invasion (%)**			0.071			0.101			0.452
pT1	16 (15.7%)	37 (26.4%)		7 (11.7%)	30 (30.0%)		3 (9.4%)	15 (21.4%)	
pT2	9 (8.8%)	20 (14.3%)		5 (8.3%)	9 (9.0%)		3 (9.4%)	11 (15.7%)	
pT3	9 (8.8%)	14 (10.0%)		12 (20.0%)	13 (13.0%)		8 (25.0%)	12 (17.1%)	
pT4a	52 (51.0%)	57 (40.7%)		26 (43.3%)	36 (36.0%)		15 (46.9%)	26 (37.1%)	
pT4b	16 (15.7%)	12 (8.6%)		10 (16.7%)	12 (12.0%)		3 (9.4%)	6 (8.6%)	
**Lymph node metastasis (%)**			0.053			0.164			0.834
pN0	37 (36.3%)	74 (52.9%)		21 (35.0%)	50 (50.0%)		9 (28.1%)	26 (37.1%)	
pN1	18 (17.6%)	24 (17.1%)		11 (18.3%)	17 (17.0%)		4 (12.5%)	10 (14.3%)	
pN2	11 (10.8%)	15 (10.7%)		7 (11.7%)	11 (11.0%)		10 (31.3%)	15 (21.4%)	
pN3a	19 (18.6%)	14 (10.0%)		15 (25.0%)	11 (11.0%)		5 (15.6%)	11 (15.7%)	
pN3b	17 (16.7%)	13 (9.3%)		6 (10.0%)	11 (11.0%)		4 (12.5%)	8 (11.4%)	
**Distant metastasis (%)**			0.693			0.601			0.128
pM0	99 (97.1%)	137 (97.9%)		59 (98.3%)	97 (97.0%)		28 (87.5%)	67 (95.7%)	
pM1	3 (2.9%)	3 (2.1%)		1 (1.7%)	3 (3.0%)		4 (12.5%)	3 (4.3%)	
**Stage (%)**			0.034			0.057			0.205
I	21 (20.6%)	52 (37.1%)		10 (16.7%)	31 (31.0%)		6 (18.8%)	22 (31.4%)	
II	21 (20.6%)	29 (20.7%)		14 (23.3%)	29 (29.0%)		3 (9.4%)	11 (15.7%)	
III	57 (55.9%)	56 (40.0%)		35 (58.3%)	37 (37.0%)		19 (59.4%)	34 (48.6%)	
IV	3 (2.9%)	3 (2.1%)		1 (1.7%)	3 (3.0%)		4 (12.5%)	3 (4.3%)	
**Chemotherapy (%)**	52 (51.0%)	72 (51.4%)	0.945	32 (53.3%)	45 (45.0%)	0.307	19 (59.4%)	27 (38.6%)	0.050

LIS, lymphoid immune score.

Supplementary Table 3. Clinical characteristics of patients according to the MIS in the training, internal, and external validation cohorts.

Variables	Training cohort, n = 242			Internal validation cohort 1, n = 160			External validation cohort 1, n = 102		
	low MIS	high MIS	*P*	low MIS	high MIS	*P*	low MIS	high MIS	*P*
**Median age (range)**	58 (48-65)	56 (46-62)	0.400	57 (50-66)	57 (49-65)	0.531	61 (55-68)	54 (43-63)	0.008
**Male (%)**	70 (64.8%)	89 (66.4%)	0.794	37 (62.7%)	77 (76.2%)	0.068	27 (65.9%)	46 (75.4%)	0.294
**Tumor size (%)**			0.796			0.842			0.058
≤4cm	55 (50.9%)	66 (49.3%)		29 (49.2%)	48 (47.5%)		26 (63.4%)	27 (44.3%)	
>4cm	53 (49.1%)	68 (50.7%)		30 (50.8%)	53 (52.5%)		15 (36.6%)	34 (55.7%)	
**Tumor location (%)**			0.749			0.052			0.019
Cardia	18 (16.7%)	28 (20.9%)		14 (23.7%)	19 (18.8%)		10 (24.4%)	23 (37.7%)	
Body	19 (17.6%)	27 (20.1%)		19 (32.2%)	17 (16.8%)		6 (14.6%)	16 (26.2%)	
Antrum	64 (59.3%)	71(53.0%)		25 (42.4%)	59 (58.4%)		25 (61.0%)	19 (31.1%)	
Whole	7 (6.5%)	8 (6.0%)		1 (1.7%)	6 (5.9%)		0 (0.0%)	3 (4.9%)	
**Differentiation status (%)**			0.254			0.600			0.665
Well	16 (14.8%)	11 (8.2%)		9 (15.3%)	10 (9.9%)		0 (0.0%)	1 (1.6%)	
Moderate	25 (23.1%)	31 (23.1%)		15 (25.4%)	27 (26.7%)		7 (17.1%)	12 (19.7%)	
Poor and undifferentiated	67 (62.0%)	92 (68.7%)		35 (59.3%)	64 (63.4%)		34 (82.9%)	48 (78.7%)	
**Lauren type (%)**			0.041			0.143			0.762
Intestinal type	43 (39.8%)	71 (53.0%)		31 (52.5%)	41 (40.6%)		16 (39.0%)	22 (36.1%)	
Diffuse or mixed type	65 (60.2%)	63 (47.0%)		28 (47.5%)	60 (59.4%)		25 (61.0%)	39 (63.9%)	
**CEA (%)**			0.121			0.757			0.513
Normal	102 (94.4%)	119 (88.8%)		51 (86.4%)	89 (88.1%)		35 (85.4%)	49 (80.3%)	
Elevated	6 (5.6%)	15 (11.2%)		8 (13.6%)	12 (11.9%)		6 (14.6%)	12 (19.7%)	
**CA19-9 (%)**			0.214			0.610			0.713
Normal	89 (82.4%)	118 (88.1%)		53 (89.8%)	88 (87.1%)		36 (87.8%)	52 (85.2%)	
Elevated	19 (17.6%)	16 (11.9%)		6 (10.2%)	13 (12.9%)		5 (12.2%)	9 (14.8%)	
**Depth of invasion (%)**			0.713			0.049			0.023
pT1	25 (23.1%)	28 (20.9%)		18 (30.5%)	19 (18.8%)		12 (29.3%)	6 (9.8%)	
pT2	13 (12.0%)	16 (11.9%)		4 (6.8%)	10 (9.9%)		7 (17.1%)	7 (11.5%)	
pT3	10 (9.3%)	13 (9.7%)		13 (22.0%)	12 (11.9%)		3 (7.3%)	17 (27.9%)	
pT4a	51 (47.2%)	58 (43.3%)		15 (25.4%)	47 (46.5%)		15 (36.6%)	26 (42.6%)	
pT4b	9 (8.3%)	19 (14.2%)		9 (15.3%)	13 (12.9%)		4 (9.8%)	5 (8.2%)	
**Lymph node metastasis (%)**			0.320			0.076			0.054
pN0	56 (51.9%)	55 (41.0%)		31 (52.5%)	40 (39.6%)		20 (48.8%)	15 (24.6%)	
pN1	20 (18.5%)	22 (16.4%)		7 (11.9%)	21 (20.8%)		2 (4.9%)	12 (19.7%)	
pN2	9 (8.3%)	17 (12.7%)		8 (13.6%)	10 (9.9%)		10 (24.4%)	15 (24.6%)	
pN3a	11 (10.2%)	22 (16.4%)		11 (18.6%)	15 (14.9%)		6 (14.6%)	10 (16.4%)	
pN3b	12 (11.1%)	18 (13.4%)		2 (3.4%)	15 (14.9%)		3 (7.3%)	9 (14.8%)	
**Distant metastasis (%)**			0.789			0.618			0.343
pM0	105 (97.2%)	131 (97.8%)		58 (98.3%)	98 (97.0%)		37 (90.2%)	58 (95.1%)	
pM1	3 (2.8%)	3 (2.2%)		1 (1.7%)	3 (3.0%)		4 (9.8%)	3 (4.9%)	
**Stage (%)**			0.189			0.268			0.005
I	34 (31.5%)	39 (29.1%)		18 (30.5%)	23 (22.8%)		18 (43.9%)	10 (16.4%)	
II	28 (25.9%)	22 (16.4%)		19 (32.2%)	24 (23.8%)		2 (4.9%)	12 (19.7%)	
III	43 (39.8%)	70 (52.2%)		21 (35.6%)	51 (50.5%)		17 (41.5%)	36 (59.0%)	
IV	3 (2.8%)	3 (2.2%)		1 (1.7%)	3 (3.0%)		4 (9.8%)	3 (4.9%)	
**Chemotherapy (%)**	58 (53.7%)	66 (49.3%)	0.491	30 (50.8%)	47 (46.5%)	0.598	14 (34.1%)	32 (52.5%)	0.068

MIS, myeloid immune score.

Supplementary Table 4. Univariate association of IS, clinicopathological characteristics with disease-free and overall survival in the training cohort.

Variables	Disease-free survival		Overall survival	
	HR (95%CI)	*p*	HR (95%CI)	*p*
LIS (high vs. low)	0.315 (0.210-0.471)	<0.0001	0.362 (0.223-0.587)	<0.0001
MIS (high vs. low)	3.396 (2.162-5.336)	<0.0001	4.399 (2.438-7.935)	<0.0001
Age (years) (≥60 vs. <60)	0.937 (0.631-1.391)	0.748	0.757 (0.462-1.240)	0.269
Sex (male vs. female)	1.043 (0.694-1.566)	0.841	1.334 (0.798-2.229)	0.271
Tumor size (>4 cm vs. ≤4 cm)	1.831 (1.235-2.715)	0.003	2.153 (1.324-3.503)	0.002
Tumor location	1.053 (0.838-1.324)	0.656	0.944 (0.719-1.240)	0.680
Differentiation	1.379 (1.011-1.881)	0.042	1.504 (1.022-2.214)	0.039
Lauren type	1.005 (0.684-1.477)	0.980	0.980 (0.613-1.567)	0.933
CEA (elevated vs. normal)	2.033 (1.176-3.515)	0.011	2.212 (1.162-4.213)	0.016
CA19-9 (elevated vs. normal)	1.477 (0.878-2.485)	0.142	1.001 (1.000-1.003)	0.081
Stage (IV vs. III vs. II vs. I)	2.123 (1.645-2.739)	<0.0001	2.954 (2.095-4.144)	<0.0001
Chemotherapy (yes vs. no)	0.686 (0.466-1.010)	0.056	0.489 (0.302-0.792)	0.004

IS, immune score; LIS, lymphoid immune score; MIS, myeloid immune score.

Supplementary Table 5. Univariate association of IS, clinicopathological characteristics with disease-free and overall survival in the internal validation cohort 1.

Variables	Disease-free survival		Overall survival	
	HR (95%CI)	*p*	HR (95%CI)	*p*
LIS (high vs. low)	0.290 (0.183-0.461)	<0.0001	0.303 (0.175-0.525)	<0.0001
MIS (high vs. low)	1.971 (1.181-3.291)	0.009	2.484 (1.305-4.726)	0.006
Age (years) (≥60 vs. <60)	1.053 (0.669-1.655)	0.824	0.890 (0.517-1.532)	0.674
Sex (male vs. female)	1.366 (0.812-2.297)	0.240	1.739 (0.896-3.374)	0.102
Tumor size (>4 cm vs. ≤4 cm)	1.867 (1.173-2.971)	0.008	1.819 (1.046-3.163)	0.034
Tumor location	0.950 (0.730-1.236)	0.702	0.963 (0.703-1.319)	0.814
Differentiation	0.966 (0.708-1.317)	0.826	1.035 (0.711-1.507)	0.856
Lauren type	0.913 (0.581-1.434)	0.692	1.197 (0.692-2.069)	0.521
CEA (elevated vs. normal)	1.365 (0.718-2.593)	0.343	1.461 (0.687-3.105)	0.324
CA19-9 (elevated vs. normal)	1.003 (0.998-1.009)	0.220	1.185 (0.535-2.624)	0.676
Stage (IV vs. III vs. II vs. I)	2.065 (1.520-2.804)	<0.0001	2.826 (1.894-4.216)	<0.0001
Chemotherapy (yes vs. no)	0.876 (0.558-1.376)	0.567	1.169 (0.684-1.997)	0.568

IS, immune score; LIS, lymphoid immune score; MIS, myeloid immune score.

Supplementary Table 6. Univariate association of IS, clinicopathological characteristics with disease-free and overall survival in the external validation cohort 1.

Variables	Disease-free survival		Overall survival	
	HR (95%CI)	*p*	HR (95%CI)	*p*
LIS (high vs. low)	0.231 (0.121-0.442)	<0.0001	0.183 (0.090-0.368)	<0.0001
MIS (high vs. low)	3.831 (1.751-8.384)	0.001	6.014 (2.307-15.674)	<0.001
Age (years) (≥60 vs. <60)	0.752 (0.394-1.435)	0.388	0.736 (0.368-1.472)	0.377
Sex (male vs. female)	1.742 (0.799-3.794)	0.163	2.081 (0.860-5.040)	0.104
Tumor size (>4 cm vs. ≤4 cm)	2.748 (1.442-5.237)	0.002	3.049 (1.518-6.128)	0.002
Tumor location	0.760 (0.538-1.073)	0.119	0.748 (0.517-1.081)	0.123
Differentiation	1.473 (0.646-3.359)	0.357	1.358 (0.592-3.114)	0.470
Lauren type	1.234 (0.634-2.402)	0.536	1.169 (0.578-2.363)	0.664
CEA (elevated vs. normal)	1.091 (0.482-2.473)	0.834	1.250 (0.544-2.875)	0.599
CA19-9 (elevated vs. normal)	4.564 (2.287-9.108)	<0.0001	4.086 (1.922-8.687)	<0.0001
Stage (IV vs. III vs. II vs. I)	2.499 (1.616-3.865)	<0.0001	2.318 (1.485-3.617)	<0.0001
Chemotherapy (yes vs. no)	1.902 (1.009-3.584)	0.047	2.004 (1.012-3.969)	0.046

IS, immune score; LIS, lymphoid immune score; MIS, myeloid immune score.

Supplementary Table 7. Multivariate cox regression analyses for disease-free survival and overall survival in patients with gastric cancer.

Variables	Disease-free survival		Overall survival	
	**HR (95%CI)**	***p***	**HR (95%CI)**	***p***
**Training cohort**				
LIS (high vs. low)	0.395 (0.261-0.599)	<0.0001	0.479 (0.290-0.791)	0.004
MIS (high vs. low)	3.193 (2.011-5.069)	<0.0001	4.113 (2.261-7.479)	<0.0001
Tumor size (>4 cm vs. ≤4 cm)	1.090 (0.708-1.677)	0.696	1.140 (0.675-1.923)	0.624
Differentiation (poor vs. moderate vs. well)	0.968 (0.683-1.373)	0.857	0.865 (0.558-1.340)	0.516
CEA (elevated vs. normal)	0.962 (0.537-1.723)	0.895	0.884 (0.440-1.778)	0.730
Stage (IV vs. III vs. II vs. I)	1.990 (1.480-2.676)	<0.0001	2.793 (1.913-4.076)	<0.0001
Chemotherapy (yes vs. no)	—	—	0.436 (0.2688-0.710)	0.001
**Internal validation cohort 1**				
LIS (high vs. low)	0.344 (0.214-0.554)	<0.0001	0.375 (0.215-0.654)	0.001
MIS (high vs. low)	1.933 (1.149-3.252)	0.013	2.220 (1.162-4.243)	0.016
Tumor size (>4 cm vs. ≤4 cm)	1.346 (0.836-2.165)	0.221	1.260 (0.719-2.210)	0.419
Stage (IV vs. III vs. II vs. I)	1.740 (1.275-2.374)	<0.0001	2.516 (1.651-3.835)	<0.0001
**External validation cohort 1**				
LIS (high vs. low)	0.431 (0.205-0.902)	0.026	0.440 (0.203-0.953)	0.037
MIS (high vs. low)	3.887 (1.502-10.058)	0.005	5.372 (1.774-16.264)	0.003
Tumor size (>4 cm vs. ≤4 cm)	1.491 (0.758-2.934)	0.247	1.464 (0.692-3.096)	0.319
CA19-9 (elevated vs. normal)	3.962 (1.816-8.642)	0.001	3.617 (1.560-8.385)	0.003
Stage (IV vs. III vs. II vs. I)	2.200 (1.356-3.569)	0.001	1.973 (1.203-3.238)	0.007
Chemotherapy (yes vs. no)	1.342 (0.703-2.562)	0.372	1.690 (0.819-3.485)	0.156

LIS, lymphoid immune score; MIS, myeloid immune score.

Supplementary Table 8. The optimal cut-off value for RS was determined using Youden's index in the training cohort.

	Cutoff	AUC (95%CI)	Sensitivity (95%CI)	Specificity (95%CI)	Accuracy	PPV	NPV
LRS	-0.1293	0.773 (0.714-0.833)	0.771 (0.693-0.838)	0.657 (0.556-0.748)	0.719	0.755 (0.676-0.823)	0.677 (0.575-0.767)
MRS	-0.2604	0.750 (0.689-0.810)	0.724 (0.640-0.798)	0.648 (0.550-0.738)	0.682	0.719 (0.635-0.792)	0.654 (0.556-0.744)

RS, radiomics score; LRS, lymphoid radiomics score; MRS, myeloid radiomics score; AUC, area under the receiver operating characteristic curve; PPV, positive predictive value; NPV, negative predictive value; RS, radiomics score.

Supplementary Table 9. Clinical characteristics of patients according to the LRS in the training and internal validation cohorts.

Variables	Training cohort, n = 242			Internal validation cohort 1, n = 160			Internal validation cohort 2, n = 512		
	low LRS	high LRS	*P*	low LRS	high LRS	*P*	low LRS	high LRS	*P*
**Median age (range)**	58 (49-64)	55 (47-64)	0.627	60 (49-65)	56 (50-65)	0.797	56 (47-64)	57 (49-63)	0.541
**Male (%)**	70 (70.7%)	89 (62.2%)	0.172	54 (77.1%)	60 (66.7%)	0.146	147 (71.0%)	204 (66.9%)	0.323
**Tumor size (%)**			<0.001			0.033			0.038
≤4cm	36 (36.4%)	85 (59.4%)		27 (38.6%)	50 (55.6%)		127 (61.4%)	214 (70.2%)	
>4cm	63 (63.6%)	58 (40.6%)		43 (61.4%)	40 (44.4%)		80 (38.6%)	91 (29.8%)	
**Tumor location (%)**			0.055			0.296			0.001
Cardia	25 (25.3%)	21 (14.7%)		17 (24.3%)	16 (17.8%)		24 (11.6%)	42 (13.8%)	
Body	13 (13.1%)	33 (23.1%)		14 (20.0%)	22 (24.4%)		40 (19.3%)	50 (16.4%)	
Antrum	53 (53.5%)	82 (57.3%)		34 (48.6%)	50 (55.6%)		114 (55.1%)	199 (65.2%)	
Whole	8 (8.1%)	7 (4.9%)		5 (7.1%)	2 (2.2%)		29 (14.0%)	14 (4.6%)	
**Differentiation status (%)**			0.439			0.180			0.213
Well	8 (8.1%)	19 (13.3%)		5 (7.1%)	14 (15.6%)		30 (14.5%)	56 (18.4%)	
Moderate	23 (23.2%)	33 (23.1%)		17 (24.3%)	25 (27.8%)		41 (19.8%)	72 (23.6%)	
Poor and undifferentiated	68 (68.7%)	91 (63.6%)		48 (68.6%)	51 (56.7%)		136 (65.7%)	177 (58.0%)	
**Lauren type (%)**			0.082			0.078			0.219
Intestinal type	40 (40.4%)	74 (51.7%)		26 (37.1%)	46 (51.1%)		87 (42.0%)	145 (47.5%)	
Diffuse or mixed type	59 (59.6%)	69 (48.3%)		44 (62.9%)	44 (48.9%)		120 (58.0%)	160 (52.5%)	
**CEA (%)**			0.263			0.718			0.049
Normal	88 (88.9%)	133 (93.0%)		62 (88.6%)	78 (86.7%)		178 (86.0%)	279 (91.5%)	
Elevated	11 (11.1%)	10 (7.0%)		8 (11.4%)	12 (13.3%)		29 (14.0%)	26 (8.5%)	
**CA19-9 (%)**			0.319			0.069			0.414
Normal	82 (82.8%)	125 (87.4%)		58 (82.9%)	83 (92.2%)		168 (81.2%)	256 (83.9%)	
Elevated	17 (17.2%)	18 (12.6%)		12 (17.1%)	7 (7.8%)		39 (18.8%)	49 (16.1%)	
**Depth of invasion (%)**			0.003			0.022			<0.001
pT1	11 (11.1%)	42 (29.4%)		10 (14.3%)	27 (30.0%)		43 (20.8%)	98 (32.1%)	
pT2	11 (11.1%)	18 (12.6%)		5 (7.1%)	9 (10.0%)		24 (11.6%)	52 (17.0%)	
pT3	10 (10.1%)	13 (9.1%)		8 (11.4%)	17 (18.9%)		4 (1.9%)	10 (3.3%)	
pT4a	49 (49.5%)	60 (42.0%)		36 (51.4%)	26 (28.9%)		64 (30.9%)	100 (32.8%)	
pT4b	18 (18.2%)	10 (7.0%)		11(15.7%)	11 (12.2%)		72 (34.8%)	45 (14.8%)	
**Lymph node metastasis (%)**			0.002			0.002			0.108
pN0	31 (31.3%)	80 (55.9%)		19 (27.1%)	52 (57.8%)		87 (42.0%)	153 (50.2%)	
pN1	20 (20.2%)	22 (15.4%)		16 (22.9%)	12 (13.3%)		51 (24.6%)	62 (20.3%)	
pN2	15 (15.2%)	11 (7.7%)		8 (11.4%)	10 (11.1%)		21 (10.1%)	41 (13.4%)	
pN3a	20 (20.2%)	13 (9.1%)		15 (21.4%)	11 (12.2%)		36 (17.4%)	34 (11.1%)	
pN3b	13 (13.1%)	17 (11.9%)		12 (17.1%)	5 (5.6%)		12 (5.8%)	15 (4.9%)	
**Distant metastasis (%)**			0.702			0.202			-
pM0	97 (98.0%)	139 (97.2%)		67 (95.7%)	89 (98.9%)		207 (100%)	305 (100%)	
pM1	2 (2.0%)	4 (2.8%)		3 (4.3%)	1 (1.1%)		0 (0.0%)	0 (0.0%)	
**Stage (%)**			0.001			0.002			0.001
I	19 (19.2%)	54 (37.8%)		11 (15.7%)	30 (33.3%)		35 (16.9%)	94 (30.8%)	
II	17 (17.2%)	33 (23.1%)		14 (20.0%)	29 (32.2%)		52 (25.1%)	71 (23.3%)	
III	61 (61.6%)	52 (36.4%)		42 (60.0%)	30 (33.3%)		120 (58.0%)	140 (45.9%)	
IV	2 (2.0%)	4 (2.8%)		3 (4.3%)	1 (1.1%)		0 (0.0%)	0 (0.0%)	
**Chemotherapy (%)**	53 (53.5%)	71 (49.7%)	0.552	39 (55.7%)	38 (42.2%)	0.090	93 (44.9%)	137 (44.9%)	0.998

LRS, lymphoid radiomics score.

Supplementary Table 10. Clinical characteristics of patients according to the MRS in the training and internal validation cohorts.

Variables	Training cohort, n = 242			Internal validation cohort 1, n = 160			Internal validation cohort 2, n = 512		
	low MRS	high MRS	*P*	low MRS	high MRS	*P*	low MRS	high MRS	*P*
**Median age (range)**	55 (48-64)	57 (47-64)	0.771	58 (50-65)	57 (48-66)	0.889	57 (49-64)	56 (48-63)	0.845
**Male (%)**	76 (71.7%)	83 (61.0%)	0.083	41 (70.7%)	73 (71.6%)	0.906	151 (74.0%)	200 (64.9%)	0.030
**Tumor size (%)**			1.000			0.764			0.828
≤4cm	53 (50.0%)	68 (50.0%)		27 (46.6%)	50 (49.0%)		137 (67.2%)	204 (66.2%)	
>4cm	53 (50.0%)	68 (50.0%)		31 (53.4%)	52 (51.0%)		67 (32.8%)	104 (33.8%)	
**Tumor location (%)**			0.206			0.911			0.018
Cardia	24 (22.6%)	22 (16.2%)		13 (22.4%)	20 (19.6%)		32 (15.7%)	34 (11.0%)	
Body	15 (14.2%)	31 (22.8%)		14 (24.1%)	22 (21.6%)		29 (14.2%)	61 (19.8%)	
Antrum	62 (58.5%)	73 (53.7%)		29 (50.0%)	55 (53.9%)		133 (65.2%)	180 (58.4%)	
Whole	5 (4.7%)	10 (7.4%)		2 (3.4%)	5 (4.9%)		10 (4.9%)	33 (10.7%)	
**Differentiation status (%)**			0.372			0.112			0.647
Well	12 (11.3%)	15 (11.0%)		11 (19.0%)	8 (7.8%)		32 (15.7%)	54 (17.5%)	
Moderate	20 (18.9%)	36 (26.5%)		14 (24.1%)	28 (27.5%)		49 (24.0%)	64 (20.8%)	
Poor and undifferentiated	74 (69.8%)	85 (62.5%)		33 (56.9%)	66 (64.7%)		123 (60.3%)	190 (61.7%)	
**Lauren type (%)**			0.039			0.197			0.937
Intestinal type	42 (39.6%)	72 (52.9%)		30 (51.7%)	42 (41.2%)		92 (45.1%)	140 (45.5%)	
Diffuse or mixed type	64 (60.4%)	64 (47.1%)		28 (48.3%)	60 (58.8%)		112 (54.9%)	168 (54.5%)	
**CEA (%)**			0.712			0.263			0.543
Normal	96 (90.6%)	125 (91.9%)		53 (91.4%)	87 (85.3%)		180 (88.2%)	277 (89.9%)	
Elevated	10 (9.4%)	11 (8.1%)		5 (8.6%)	15 (14.7%)		24 (11.8%)	31 (10.1%)	
**CA19-9 (%)**			0.037			0.572			0.622
Normal	85 (80.2%)	122 (89.7%)		50 (86.2%)	91 (89.2%)		171 (83.8%)	253 (82.1%)	
Elevated	21 (19.8%)	14 (10.3%)		8 (13.8%)	11 (10.8%)		33 (16.2%)	55 (17.9%)	
**Depth of invasion (%)**			0.344			0.677			0.251
pT1	24 (22.6%)	29 (21.3%)		16 (27.6%)	21 (20.6%)		64 (31.4%)	77 (25.0%)	
pT2	13 (12.3%)	16 (11.8%)		3 (5.2%)	11 (10.8%)		32 (15.7%)	44 (14.3%)	
pT3	8 (7.5%)	15 (11.0%)		9 (15.5%)	16 (15.7%)		4 (2.0%)	10 (3.2%)	
pT4a	53 (50.0%)	56 (41.2%)		23 (39.7%)	39 (38.2%)		66 (32.4%)	98 (31.8%)	
pT4b	8 (7.5%)	20 (14.7%)		7 (12.1%)	15 (14.7%)		38 (18.6%)	79 (25.6%)	
**Lymph node metastasis (%)**			0.636			0.384			0.119
pN0	51 (48.1%)	60 (44.1%)		26 (44.8%)	45 (44.1%)		95 (46.6%)	145 (47.1%)	
pN1	17 (16.0%)	25 (18.4%)		11 (19.0%)	17 (16.7%)		40 (19.6%)	73 (23.7%)	
pN2	10 (9.4%)	16 (11.8%)		9 (15.5%)	9 (8.8%)		34 (16.7%)	28 (9.1%)	
pN3a	12 (11.3%)	21 (15.4%)		9 (15.5%)	17 (16.7%)		25 (12.3%)	45 (14.6%)	
pN3b	16 (15.1%)	14 (10.3%)		3 (5.2%)	14 (13.7%)		10 (4.9%)	17 (5.5%)	
**Distant metastasis (%)**			0.601			0.635			-
pM0	104 (98.1%)	132 (97.1%)		57 (98.3%)	99 (97.1%)		204 (100%)	308 (100%)	
pM1	2 (1.9%)	4 (2.9%)		1 (1.7%)	3 (2.9%)		0(0.0%)	0(0.0%)	
**Stage (%)**			0.824			0.812			0.590
I	33 (31.1%)	40 (29.4%)		14 (24.1%)	27 (26.5%)		55 (27.0%)	74 (24.0%)	
II	24 (22.6%)	26 (19.1%)		18 (31.0%)	25 (24.5%)		51 (25.0%)	72 (23.4%)	
III	47 (44.3%)	66 (48.5%)		25 (43.1%)	47 (46.1%)		98 (48.0%)	162 (52.6%)	
IV	2 (1.9%)	4 (2.9%)		1 (1.7%)	3 (2.9%)		0 (0.0%)	0 (0.0%)	
**Chemotherapy (%)**	54 (50.9%)	70 (51.5)	0.935	26 (44.8%)	51 (50.0%)	0.529	92 (45.1%)	138 (44.8%)	0.948

MRS, myeloid radiomics score.

Supplementary Table 11. Clinical characteristics of patients according to the LRS in the external validation cohorts and prospective validation cohort.

Variables	External validation cohort 1, n =160			External validation cohort 2, n = 1123			Prospective validation cohort, n = 158		
	low LRS	high LRS	*P*	low LRS	high LRS	*P*	low LRS	high LRS	*P*
**Median age (range)**	53 (42-60)	61 (53-66)	0.005	57 (50-65)	58 (49-65)	0.967	60 (48-66)	56 (50-64)	0.785
**Male (%)**	32 (86.5%)	41 (63.1%)	0.012	351 (71.1%)	422 (67.1%)	0.155	47 (65.3%)	57 (66.3%)	0.895
**Tumor size (%)**			0.613			<0.001			<0.001
≤4cm	18 (48.6%)	35 (53.8%)		139 (28.1%)	301 (47.9%)		36 (50.0%)	71 (82.6%)	
>4cm	19 (51.4%)	30 (46.2%)		355 (71.9%)	328 (52.1%)		36 (50.0%)	15 (17.4%)	
**Tumor location (%)**			0.457			<0.001			0.681
Cardia	14 (37.8%)	19 (29.2%)		174 (35.2%)	200 (31.8%)		15 (20.8%)	18 (20.9%)	
Body	8 (21.6%)	14 (21.5%)		109 (22.1%)	127 (20.2%)		16 (22.2%)	14 (16.3%)	
Antrum	13 (35.1%)	31 (47.7%)		173 (35.0%)	286 (45.5%)		40 (55.6%)	51 (59.3%)	
Whole	2 (5.4%)	1 (1.5%)		38 (7.7%)	16 (2.5%)		1 (1.4%)	3 (3.5%)	
**Differentiation status (%)**			0.220			0.439			0.220
Well	0 (0.0%)	1 (1.5%)		6 (1.2%)	14 (2.2%)		6 (8.3%)	6 (7.0%)	
Moderate	4 (10.8%)	15 (23.1%)		78 (15.8%)	96 (15.3%)		9 (12.5%)	20 (23.3%)	
Poor and undifferentiated	33 (89.2%)	49 (75.4%)		410 (83.0%)	519 (82.5%)		57 (79.2%)	60 (69.8%)	
**Lauren type (%)**			0.236			0.122			0.813
Intestinal type	11 (29.7%)	27 (41.5%)		155 (31.4%)	225 (35.8%)		23 (31.9%)	29 (33.7%)	
Diffuse or mixed type	26 (70.3%)	38 (58.5%)		339 (68.6%)	404 (64.2%)		49 (68.1%)	57 (66.3%)	
**CEA (%)**			0.409			0.003			0.529
Normal	32 (86.5%)	52 (80.0%)		381 (77.1%)	529 (84.1%)		68 (94.4%)	83 (96.5%)	
Elevated	5 (13.5%)	13 (20.0%)		113 (22.9%)	100 (15.9%)		4 (5.6%)	3 (3.5%)	
**CA19-9 (%)**			0.581			<0.001			0.362
Normal	31 (83.8%)	57 (87.7%)		372 (75.3%)	536 (85.2%)		64 (88.9%)	80 (93.0%)	
Elevated	6 (16.2%)	8 (12.3%)		122 (24.7%)	93 (14.8%)		8 (11.1%)	6 (7.0%)	
**Depth of invasion (%)**			0.623			<0.001			<0.001
pT1	4 (10.8%)	14 (21.5%)		32 (6.5%)	116 (18.4%)		8 (11.1%)	41 (47.7%)	
pT2	5 (13.5%)	9 (13.8%)		36 (7.3%)	94 (14.9%)		9 (12.5%)	16 (18.6%)	
pT3	7 (18.9%)	13 (20.0%)		92 (18.6%)	147 (23.4%)		25 (34.7%)	16 (18.6%)	
pT4a	18 (48.6%)	23 (35.4%)		272 (55.1%)	244 (38.8%)		22 (30.6%)	10 (11.6%)	
pT4b	3 (8.1%)	6 (9.2%)		62 (12.6%)	28 (4.5%)		8 (11.1%)	3 (3.5%)	
**Lymph node metastasis (%)**			0.384			<0.001			0.002
pN0	10 (27.0%)	25 (38.5%)		112 (22.7%)	257 (40.9%)		22 (30.6%)	50 (58.1%)	
pN1	6 (16.2%)	8 (12.3%)		73 (14.8%)	104 (16.5%)		10 (13.9%)	12 (14.0%)	
pN2	8 (21.6%)	17 (26.2%)		94 (19.0%)	105 (16.7%)		13 (18.1%)	13 (15.1%)	
pN3a	9 (24.3%)	7 (10.8%)		129 (26.1%)	117 (18.6%)		15 (20.8%)	7 (8.1%)	
pN3b	4 (10.8%)	8 (12.3%)		86 (17.4%)	46 (7.3%)		12 (16.7%)	4 (4.7%)	
**Distant metastasis (%)**			0.234			0.001			0.667
pM0	33 (89.2%)	62 (95.4%)		437 (88.5%)	592 (94.1%)		71 (98.6%)	84 (97.7%)	
pM1	4 (10.8%)	3 (4.6%)		57 (11.5%)	37 (5.9%)		1 (1.4%)	2 (2.3%)	
**Stage (%)**			0.448			<0.001			<0.001
I	8 (21.6%)	20 (30.8%)		49 (9.9%)	155 (24.6%)		12 (16.7%)	45 (52.3%)	
II	4 (10.8%)	10 (15.4%)		84 (17.0%)	193 (30.7%)		20 (27.8%)	20 (23.3%)	
III	21 (56.8%)	32 (49.2%)		304 (61.5%)	244 (38.8%)		39 (54.2%)	19 (22.1%)	
IV	4 (10.8%)	3 (4.6%)		57 (11.5%)	37 (5.9%)		1 (1.4%)	2 (2.3%)	
**Chemotherapy (%)**	21 (56.8%)	25 (38.5%)	0.074	228 (46.2%)	309 (49.1%)	0.322	49 (68.1%)	39 (45.3%)	0.004

LRS, lymphoid radiomics score.

Supplementary Table 12. Clinical characteristics of patients according to the MRS in the external validation cohorts and prospective validation cohort.

Variables	External validation cohort 1, n =160			External validation cohort 2, n = 1123			Prospective validation cohort, n = 158		
	low MRS	high MRS	*P*	low MRS	high MRS	*P*	low MRS	high MRS	*P*
**Median age (range)**	60 (51-66)	55 (45-64)	0.263	57 (48-64)	57 (49-65)	0.527	58 (50-66)	56 (48-65)	0.546
**Male (%)**	28 (62.2%)	45 (78.9%)	0.063	329 (69.1%)	444 (68.6%)	0.860	52 (68.4%)	52 (63.4%)	0.507
**Tumor size (%)**			0.065			<0.001			0.026
≤4cm	28 (62.2%)	25 (43.9%)		230 (48.3%)	210 (32.5%)		58 (76.3%)	49 (59.8%)	
>4cm	17 (37.8%)	32 (56.1%)		246 (51.7%)	437 (67.5%)		18 (23.7%)	33 (40.2%)	
**Tumor location (%)**			0.159			<0.001			0.436
Cardia	12 (26.7%)	21 (36.8%)		145 (30.5%)	229 (35.4%)		20 (26.3%)	13 (15.9%)	
Body	7 (15.6%)	15 (26.3%)		83 (17.4%)	153 (23.6%)		14 (18.4%)	16 (19.5%)	
Antrum	25 (55.6%)	19 (33.3%)		237 (49.8%)	222 (34.3%)		40 (52.6%)	51 (62.2%)	
Whole	1 (2.2%)	2 (3.5%)		11 (2.3%)	43 (6.6%)		2 (2.6%)	2 (2.4%)	
**Differentiation status (%)**			0.492			0.112			0.337
Well	1 (2.2%)	0 (0.0%)		13 (2.7%)	7 (1.1%)		8 (10.5%)	4 (4.9%)	
Moderate	9 (20.0%)	10 (17.5%)		71 (14.9%)	103 (15.9%)		15 (19.7%)	14 (17.1%)	
Poor and undifferentiated	35 (77.8%)	47 (82.5%)		392 (82.4%)	537 (83.0%)		53 (69.7%)	64 (78.0%)	
**Lauren type (%)**			0.610			0.254			0.501
Intestinal type	18 (40.0%)	20 (35.1%)		170 (35.7%)	210 (32.5%)		27 (35.5%)	25 (30.5%)	
Diffuse or mixed type	27 (60.0%)	37 (64.9%)		306 (64.3%)	437 (67.5%)		49 (64.5%)	57 (69.5%)	
**CEA (%)**			0.580			0.041			0.776
Normal	36 (80.0%)	48 (84.2%)		399 (83.8%)	511 (79.0%)		73 (96.1%)	78 (95.1%)	
Elevated	9 (20.0%)	9 (15.8%)		167 (16.2%)	136 (21.0%)		3 (3.9%)	4 (4.9%)	
**CA19-9 (%)**			0.066			<0.001			0.331
Normal	42 (93.3%)	46 (80.7%)		410 (86.1%)	498 (77.0%)		71 (93.4%)	73 (89.0%)	
Elevated	3 (6.7%)	11 (19.3%)		66 (13.9%)	149 (23.0%)		5 (6.6%)	9 (11.0%)	
**Depth of invasion (%)**			0.755			<0.001			0.029
pT1	9 (20.0%)	9 (15.8%)		87 (18.3%)	61 (9.4%)		29 (38.2%)	20 (24.4%)	
pT2	8 (17.8%)	6 (10.5%)		68 (14.3%)	62 (9.6%)		16 (21.1%)	9 (11.0%)	
pT3	8 (17.8%)	12 (21.1%)		105 (22.1%)	134 (20.7%)		18 (23.7%)	23 (28.0%)	
pT4a	17 (37.8%)	24 (42.1%)		187 (39.3%)	329 (50.9%)		10 (13.2%)	22 (26.8%)	
pT4b	3 (6.7%)	6 (10.5%)		29 (6.1%)	61 (9.4%)		3 (3.9%)	8 (9.8%)	
**Lymph node metastasis (%)**			0.512			0.001			0.002
pN0	19 (42.2%)	16 (28.1%)		188 (39.5%)	181 (28.0%)		46 (60.5%)	26 (31.7%)	
pN1	5 (11.1%)	9 (15.8%)		75 (15.8%)	102 (15.8%)		10 (13.2%)	12 (14.6%)	
pN2	10 (22.2%)	15 (26.3%)		77 (16.2%)	122 (18.9%)		11 (14.5%)	15 (18.3%)	
pN3a	5 (11.1%)	11 (19.3%)		90 (18.9%)	156 (24.1%)		5 (6.6%)	17 (20.7%)	
pN3b	6 (13.3%)	6 (10.5%)		46 (9.7%)	86 (13.3%)		4 (5.3%)	12 (14.6%)	
**Distant metastasis (%)**			0.100			0.087			0.605
pM0	44 (97.8%)	51 (89.5%)		444 (93.3%)	585 (90.4%)		75 (98.7%)	80 (97.6%)	
pM1	1 (2.2%)	6 (10.5%)		32 (6.7%)	62 (9.6%)		1 (1.3%)	2 (2.4%)	
**Stage (%)**			0.182			<0.001			0.001
I	16 (35.6%)	12 (21.1%)		114 (23.9%)	90 (13.9%)		37 (48.7%)	20 (24.4%)	
II	5 (11.1%)	9 (15.8%)		136 (28.6%)	141 (21.8%)		22 (28.9%)	18 (22.0%)	
III	23 (51.1%)	30 (52.6%)		194 (40.8%)	354 (54.7%)		16 (21.1%)	42 (51.2%)	
IV	1 (2.2%)	6 (10.5%)		32 (6.7%)	62 (9.6%)		1 (1.3%)	2 (2.4%)	
**Chemotherapy (%)**	15 (33.3%)	31 (54.4%)	0.034	226 (47.5%)	311 (48.1%)	0.845	34 (44.7%)	54 (65.9%)	0.008

MRS, myeloid radiomics score.

Supplementary Table 13. Clinical characteristics of patients according to the combined score in the training cohort and internal validation cohort 1.

Variables	Training cohort, n =242					Internal validation cohort 1, n = 160				
	Combined score (LRS/MRS)					Combined score (LRS/MRS)				
	1 (−/−)	2 (+/−)	3 (−/+)	4 (+/+)	*P*	1 (−/−)	2 (+/−)	3 (−/+)	4 (+/+)	*P*
**Median age (range)**	60 (49-66)	55 (47-61)	58 (48-62)	56 (47-65)	0.860	63 (53-65)	56 (50-63)	59 (45-65)	56 (50-66)	0.799
**Male**	27 (73.0%)	49 (71.0%)	43 (69.4%)	40 (54.1%)	0.088	11 (64.7%)	30 (73.2%)	43 (81.1%)	30 (61.2%)	0.147
**Tumor size**					0.003					0.033
≤4cm	15 (40.5%)	38 (55.1%)	21 (33.9%)	47 (63.5%)		3 (17.6%)	24 (58.5%)	24 (45.3%)	26 (53.1%)	
>4cm	22 (59.5%)	31 (44.9%)	41 (66.1%)	27 (36.5%)		14 (82.4%)	17 (41.5%)	29 (54.7%)	23 (46.9%)	
**Tumor location**					0.130					0.859
Cardia	12 (32.4%)	12 (17.4%)	13 (21.0%)	9 (12.2%)		5 (29.4%)	8 (19.5%)	12 (22.6%)	8 (16.3%)	
Body	2 (5.4%)	13 (18.8%)	11 (17.7%)	20 (27.0%)		3 (17.6%)	11 (26.8%)	11 (20.8%)	11 (22.4%)	
Antrum	21 (56.8%)	41 (59.4%)	32 (51.6%)	41 (55.4%)		8 (47.1%)	21 (51.2%)	26 (49.1%)	29 (59.2%)	
Whole	2 (5.4%)	3 (4.3%)	6 (9.7%)	4 (5.4%)		1 (5.9%)	1 (2.4%)	4 (7.5%)	1 (2.0%)	
**Differentiation status**					0.323					0.076
Well	3 (8.1%)	9 (13.0%)	5 (8.1%)	10 (13.5%)		3 (17.6%)	8 (19.5%)	2 (3.8%)	6 (12.2%)	
Moderate	4 (10.8%)	16 (23.2%)	19 (30.6%)	17 (23.0%)		6 (35.3%)	8 (19.5%)	11 (20.8%)	17 (34.7%)	
Poor and undifferentiated	30 (81.1%)	44 (63.8%)	38 (61.3%)	47 (63.5%)		8 (47.1%)	25 (61.0%)	40 (75.5%)	26 (53.1%)	
**Lauren type**					0.019					0.241
Intestinal type	9 (24.3%)	33 (47.8%)	31 (50.0%)	41 (55.4%)		8 (47.1%)	22 (53.7%)	18 (34.0%)	24 (49.0%)	
Diffuse or mixed type	28 (75.7%)	36 (52.2%)	31 (50.0%)	33 (44.6%)		9 (52.9%)	19 (46.3%)	35 (66.0%)	25 (51.0%)	
**CEA**					0.627					0.453
Normal	33 (89.2%)	63 (91.3%)	55 (88.7%)	70 (94.6%)		15 (88.2%)	38 (92.7%)	47 (88.7%)	40 (81.6%)	
Elevated	4 (10.8%)	6 (8.7%)	7 (11.3%)	4 (5.4%)		2 (11.8%)	3 (7.3%)	6 (11.3%)	9 (18.4%)	
**CA19-9**					0.030					0.088
Normal	26 (70.3%)	59 (85.5%)	56 (90.3%)	66 (89.2%)		12 (70.6%)	38 (92.7%)	46 (86.8%)	45 (91.8%)	
Elevated	11 (29.7%)	10 (14.5%)	6 (9.7%)	8 (10.8%)		5 (29.4%)	3 (7.3%)	7 (13.2%)	4 (8.2%)	
**Depth of invasion**					0.008					0.068
pT1	4 (10.8%)	20 (29.0%)	7 (11.3%)	22 (29.7%)		1 (5.9%)	15 (36.6%)	9 (17.0%)	12 (24.5%)	
pT2	6 (16.2%)	7 (10.1%)	5 (8.1%)	11 (14.9%)		2 (11.8%)	1 (2.4%)	3 (5.7%)	8 (16.3%)	
pT3	1 (2.7%)	7 (10.1%)	9 (14.5%)	6 (8.1%)		2 (11.8%)	7 (17.1%)	6 (11.3%)	10 (20.4%)	
pT4a	19 (51.4%)	34 (49.3%)	30 (48.4%)	26 (35.1%)		10 (58.8%)	13 (31.7%)	26 (49.1%)	13 (26.5%)	
pT4b	7 (18.9%)	1 (1.4%)	11 (17.7%)	9 (12.2%)		2 (11.8%)	5 (12.2%)	9 (17.0%)	6 (12.2%)	
**Lymph node metastasis**					0.004					0.009
pN0	7 (18.9%)	44 (63.8%)	24 (38.7%)	36 (48.6%)		4 (23.5%)	22 (53.7%)	15 (28.3%)	30 (61.2%)	
pN1	10 (27.0%)	7 (10.1%)	10 (16.1%)	15 (20.3%)		3 (17.6%)	8 (19.5%)	13 (24.5%)	4 (8.2%)	
pN2	6 (16.2%)	4 (5.8%)	9 (14.5%)	7 (9.5%)		4 (23.5%)	5 (12.2%)	4 (7.5%)	5 (10.2%)	
pN3a	6 (16.2%)	6 (8.7%)	14 (22.6%)	7 (9.5%)		5 (29.4%)	4 (9.8%)	10 (18.9%)	7 (14.3%)	
pN3b	8 (21.6%)	8 (11.6%)	5 (8.1%)	9 (12.2%)		1 (5.9%)	2 (4.9%)	11 (20.8%)	3 (6.1%)	
**Distant metastasis**					0.765					0.523
pM0	37 (100%)	67 (97.1%)	60 (96.8%)	72 (97.3%)		16 (94.1%)	41 (100%)	51 (96.2%)	48 (98.0%)	
pM1	0 (0.0%)	2 (2.9%)	2 (3.2%)	2 (2.7%)		1 (5.9%)	0 (0.0%)	2 (3.8%)	1 (2.0%)	
**Stage**					0.001					0.055
I	8 (21.6%)	25 (36.2%)	11 (17.7%)	29 (39.2%)		1 (5.9%)	13 (31.7%)	10 (18.9%)	17 (34.7%)	
II	2 (5.4%)	22 (31.9%)	15 (24.2%)	11 (14.9%)		4 (23.5%)	14 (34.1%)	10 (18.9%)	15 (30.6%)	
III	27 (73.0%)	20 (29.0%)	34 (54.8%)	32 (43.2%)		11 (64.7%)	14 (34.1%)	31 (58.5%)	16 (32.7%)	
IV	0 (0.0%)	2 (2.9%)	2 (3.2%)	2 (2.7%)		1 (5.9%)	0 (0.0%)	2 (3.8%)	1 (2.0%)	
**Chemotherapy**	19 (51.4%)	36 (52.2%)	35 (56.5%)	35 (47.3%)	0.739	12 (70.6%)	14 (34.1%)	27 (50.9%)	24 (49.0%)	0.078

LRS, lymphoid radiomics score; MRS, myeloid radiomics score. 1, L-LRS and L-MRS; 2, H-LRS and L-MRS; 3, L-LRS and H-MRS; 4, H-LRS and H-MRS.

Supplementary Table 14. Clinical characteristics of patients according to the combined score in the internal validation cohort 2 and prospective validation cohort.

Variables	Internal validation cohort 2, n = 512					Prospective validation cohort, n = 158				
	Combined score (LRS/MRS)					Combined score (LRS/MRS)				
	1 (−/−)	2 (+/−)	3 (−/+)	4 (+/+)	*P*	1 (−/−)	2 (+/−)	3 (−/+)	4 (+/+)	*P*
**Median age (range)**	57 (48-64)	57 (49-64)	55 (47-65)	56 (48-62)	0.829	61 (50-67)	55 (50-64)	55 (46-65)	57 (50-63)	0.593
**Male**	46 (82.1%)	105 (70.9%)	101 (66.9%)	99 (63.1%)	0.055	20 (76.9%)	32 (64.0%)	27 (58.7%)	25 (69.4%)	0.432
**Tumor size**					0.212					<0.001
≤4cm	33 (58.9%)	104 (70.3%)	94 (62.3%)	110 (70.1%)		15 (57.7%)	43 (86.0%)	21 (45.7%)	28 (77.8%)	
>4cm	23 (41.1%)	44 (29.7%)	57 (37.7%)	47 (29.9%)		11 (42.3%)	7 (14.0%)	25 (54.3%)	8 (22.2%)	
**Tumor location**					0.006					0.781
Cardia	7 (12.5%)	25 (16.9%)	17 (11.3%)	17 (10.8%)		8 (30.8%)	12 (24.0%)	7 (15.2%)	6 (16.7%)	
Body	8 (14.3%)	21 (14.2%)	32 (21.2%)	29 (18.5%)		5 (19.2%)	9 (18.0%)	11 (23.9%)	5 (13.9%)	
Antrum	35 (62.5%)	98 (66.2%)	79 (52.3%)	101 (64.3%)		13 (50.0%)	27 (54.0%)	27 (58.7%)	24 (66.7%)	
Whole	6 (10.7%)	4 (2.7%)	23 (15.2%)	10 (6.4%)		0 (0.0%)	2 (4.0%)	1 (2.2%)	1 (2.8%)	
**Differentiation status**					0.639					0.108
Well	7 (12.5%)	25 (16.9%)	23 (15.2%)	31 (19.7%)		3 (11.5%)	5 (10.0%)	3 (6.5%)	1 (2.8%)	
Moderate	11 (19.6%)	38 (25.7%)	30 (19.9%)	34 (21.7%)		6 (23.1%)	9 (18.0%)	3 (6.5%)	11 (30.6%)	
Poor and undifferentiated	38 (67.9%)	85 (57.4%)	98 (64.9%)	92 (58.6%)		17 (65.4%)	36 (72.0%)	40 (87.0%)	24 (66.7%)	
**Lauren type**					0.538					0.533
Intestinal type	21 (37.5%)	71 (48.0%)	66 (43.7%)	74 (47.1%)		11 (42.3%)	16 (32.0%)	12 (26.1%)	13 (36.1%)	
Diffuse or mixed type	35 (62.5%)	77 (52.0%)	85 (56.3%)	83 (52.9%)		15 (57.7%)	34 (68.0%)	34 (73.9%)	23 (63.9%)	
**CEA**					0.092					0.861
Normal	45 (80.4%)	135 (91.2%)	133 (88.1%)	144 (91.7%)		25 (96.2%)	48 (96.0%)	43 (93.5%)	35 (97.2%)	
Elevated	11 (19.6%)	13 (8.8%)	18 (11.9%)	13 (8.3%)		1 (3.8%)	2 (4.0%)	3 (6.5%)	1 (2.8%)	
**CA19-9**					0.855					0.669
Normal	46 (82.1%)	125 (84.5%)	122 (80.8%)	131 (83.4%)		24 (92.3%)	47 (94.0%)	40 (87.0%)	33 (91.7%)	
Elevated	10 (17.9%)	23 (15.5%)	29 (19.2%)	26 (16.6%)		2 (7.7%)	3 (6.0%)	6 (13.0%)	3 (8.3%)	
**Depth of invasion**					<0.001					<0.001
pT1	17 (30.4%)	47 (31.8%)	26 (17.2%)	51 (32.5%)		3 (11.5%)	26 (52.0%)	5 (10.9%)	15 (41.7%)	
pT2	8 (14.3%)	24 (16.2%)	16 (10.6%)	28 (17.8%)		6 (23.1%)	10 (20.0%)	3 (6.5%)	6 (16.7%)	
pT3	1 (1.8%)	3 (2.0%)	3 (2.0%)	7 (4.5%)		7 (26.9%)	11 (22.0%)	18 (39.1%)	5 (13.9%)	
pT4a	17 (30.4%)	49 (33.1%)	47 (31.1%)	51 (32.5%)		7 (26.9%)	3 (6.0%)	15 (32.6%)	7 (19.4%)	
pT4b	13 (23.2%)	25 (16.9%)	59 (39.1%)	20 (12.7%)		3 (11.5%)	0 (0.0%)	5 (10.9%)	3 (8.3%)	
**Lymph node metastasis**					0.250					0.001
pN0	22 (39.3%)	73 (49.3%)	65 (43.0%)	80 (51.0%)		13 (50.0%)	33 (66.0%)	9 (19.6%)	17 (47.2%)	
pN1	14 (25.0%)	26 (17.6%)	37 (24.5%)	36 (22.9%)		5 (19.2%)	5 (10.0%)	5 (10.9%)	7 (19.4%)	
pN2	7 (12.5%)	27 (18.2%)	14 (9.3%)	14 (8.9%)		3 (11.5%)	8 (16.0%)	10 (21.7%)	5 (13.9%)	
pN3a	10 (17.9%)	15 (10.1%)	26 (17.2%)	19 (12.1%)		3 (11.5%)	2 (4.0%)	12 (26.1%)	5 (13.9%)	
pN3b	3 (5.4%)	7 (4.7%)	9 (6.0%)	8 (5.1%)		2 (7.7%)	2 (4.0%)	10 (21.7%)	2 (5.6%)	
**Distant metastasis**					-					0.879
pM0	56 (100%)	148 (100%)	151 (100%)	157 (100%)		26 (100%)	49 (98.0%)	45 (97.8%)	35 (97.2%)	
pM1	0 (0.0%)	0 (0.0%)	0 (0.0%)	0 (0.0%)		0 (0.0%)	1 (2.0%)	1 (2.2%)	1 (2.8%)	
**Stage**					0.006					<0.001
I	14 (25.0%)	41 (27.7%)	21 (13.9%)	53 (33.8%)		6 (23.1%)	31 (62.0%)	6 (13.0%)	14 (38.9%)	
II	15 (26.8%)	36 (24.3%)	37 (24.5%)	35 (22.3%)		12 (46.2%)	10 (20.0%)	8 (17.4%)	10 (27.8%)	
III	27 (48.2%)	71 (48.0%)	93 (61.6%)	69 (43.9%)		8 (30.8%)	8 (16.0%)	31 (67.4%)	11 (30.6%)	
IV	0 (0.0%)	0 (0.0%)	0 (0.0%)	0 (0.0%)		0 (0.0%)	1 (2.0%)	1 (2.2%)	1 (2.8%)	
**Chemotherapy**	26 (46.4%)	66 (44.6%)	67 (44.4%)	71 (45.2%)	0.994	15 (57.7%)	19 (38.0%)	34 (73.9%)	20 (55.6%)	0.006

LRS, lymphoid radiomics score; MRS, myeloid radiomics score. 1, L-LRS and L-MRS; 2, H-LRS and L-MRS; 3, L-LRS and H-MRS; 4, H-LRS and H-MRS.

Supplementary Table 15. Clinical characteristics of patients according to the combined score in the external validation cohort 1 and external validation cohort 2.

Variables	External validation cohort 1, n = 102					External validation cohort 2, n = 1123				
	Combined score (LRS/MRS)					Combined score (LRS/MRS)				
	1 (−/−)	2 (+/−)	3 (−/+)	4 (+/+)	*P*	1 (−/−)	2 (+/−)	3 (−/+)	4 (+/+)	*P*
**Median age (range)**	58 (44-61)	61 (51-67)	51 (42-60)	60 (55-66)	0.039	57 (48-64)	58 (49-65)	57 (50-65)	57 (48-65)	0.874
**Male**	7 (100%)	22 (57.9%)	25 (83.3%)	19 (70.4%)	0.040	94 (74.6%)	235 (67.1%)	257 (69.8%)	187 (67.0%)	0.389
**Tumor size**					0.544					<0.001
≤4cm	4 (57.1%)	23 (60.5%)	14 (46.7%)	12 (44.4%)		50 (39.7%)	180 (51.4%)	89 (24.2%)	121 (43.4%)	
>4cm	3 (42.9%)	15 (39.5%)	16 (53.3%)	15 (55.6%)		76 (60.3%)	170 (48.6%)	279 (75.8%)	158 (56.6%)	
**Tumor location**					0.344					<0.001
Cardia	1 (14.3%)	11 (28.9%)	13 (43.3%)	8 (29.6%)		39 (31.0%)	106 (30.3%)	135 (36.7%)	94 (33.7%)	
Body	1 (14.3%)	6 (15.8%)	7 (23.3%)	8 (29.6%)		23 (18.3%)	60 (17.1%)	86 (23.4%)	67 (24.0%)	
Antrum	5 (71.4%)	20 (52.6%)	8 (26.7%)	11 (40.7%)		60 (47.6%)	177 (50.6%)	113 (30.7%)	109 (39.1%)	
Whole	0 (0.0%)	1 (2.6%)	2 (6.7%)	0 (0.0%)		4 (3.2%)	7 (2.0%)	34 (9.2%)	9 (3.2%)	
**Differentiation status**					0.561					0.392
Well	0 (0.0%)	1 (2.6%)	0 (0.0%)	0 (0.0%)		2 (1.6%)	11 (3.1%)	4 (1.1%)	3 (1.1%)	
Moderate	0 (0.0%)	9 (23.7%)	4 (13.3%)	6 (22.2%)		17 (13.5%)	54 (15.4%)	61 (16.6%)	42 (15.1%)	
Poor and undifferentiated	7 (100%)	28 (73.7%)	26 (86.7%)	21 (77.8%)		107 (84.9%)	285 (81.4%)	303 (82.3%)	234 (83.9%)	
**Lauren type**					0.167					0.217
Intestinal type	0 (0.0%)	17 (44.7%)	11 (36.7%)	10 (37.0%)		37 (29.4%)	133 (38.0%)	118 (32.1%)	92 (33.0%)	
Diffuse or mixed type	7 (100%)	21 (55.3%)	19 (63.3%)	17 (63.0%)		89 (70.6%)	217 (62.0%)	250 (67.9%)	187 (67.0%)	
**CEA**					0.454					0.007
Normal	7 (100%)	29 (76.3%)	25 (83.3%)	23 (85.2%)		104 (82.5%)	295 (84.3%)	277 (75.3%)	234 (83.9%)	
Elevated	0 (0.0%)	9 (23.7%)	5 (16.7%)	4 (14.8%)		22 (17.5%)	55 (15.7%)	91 (24.7%)	45 (16.1%)	
**CA19-9**					0.244					<0.001
Normal	6 (85.7%)	36 (94.7%)	25 (83.3%)	21 (77.8%)		104 (82.5%)	306 (87.4%)	268 (72.8%)	230 (82.4%)	
Elevated	1 (14.3%)	2 (5.3%)	5 (16.7%)	6 (22.2%)		22 (17.5%)	44 (12.6%)	100 (27.2%)	49 (17.6%)	
**Depth of invasion**					0.934					<0.001
pT1	1 (14.3%)	8 (21.1%)	3 (10.0%)	6 (22.2%)		14 (11.1%)	73 (20.9%)	18 (4.9%)	43 (15.4%)	
pT2	1 (14.3%)	6 (15.8%)	4 (13.3%)	3 (11.1%)		11 (8.7%)	57 (16.3%)	25 (6.8%)	37 (13.3%)	
pT3	1 (14.3%)	7 (18.4%)	6 (20.0%)	6 (22.2%)		24 (19.0%)	81 (23.1%)	68 (18.5%)	66 (23.7%)	
pT4a	3 (42.9%)	15 (39.5%)	15 (50.0%)	8 (29.6%)		60 (47.6%)	127 (36.3%)	212 (57.6%)	117 (41.9%)	
pT4b	1 (14.3%)	2 (5.3%)	2 (6.7%)	4 (14.8%)		17 (13.5%)	12 (3.4%)	45 (12.2%)	16 (5.7%)	
**Lymph node metastasis**					0.583					<0.001
pN0	3 (42.9%)	16 (42.1%)	7 (23.3%)	9 (33.3%)		33 (26.2%)	155 (44.3%)	79 (21.5%)	102 (36.6%)	
pN1	0 (0.0%)	5 (13.2%)	6 (20.0%)	3 (11.1%)		15 (11.9%)	60 (17.1%)	58 (15.8%)	44 (15.8%)	
pN2	2 (28.6%)	8 (21.1%)	6 (20.0%)	9 (33.3%)		28 (22.2%)	49 (14.0%)	66 (17.9%)	56 (20.1%)	
pN3a	2 (28.6%)	3 (7.9%)	7 (23.3%)	4 (14.8%)		30 (23.8%)	60 (17.1%)	99 (26.9%)	57 (20.4%)	
pN3b	0 (0.0%)	6 (15.8%)	4 (13.3%)	2 (7.4%)		20 (15.9%)	26 (7.4%)	66 (17.9%)	20 (7.2%)	
**Distant metastasis**					0.543					0.004
pM0	6 (85.7%)	37 (97.4%)	27 (90.0%)	25 (92.6%)		115 (91.3%)	329 (94.0%)	322 (87.5%)	263 (94.3%)	
pM1	1 (14.3%)	1 (2.6%)	3 (10.0%)	2 (7.4%)		11 (8.7%)	21 (6.0%)	46 (12.5%)	16 (5.7%)	
**Stage**					0.735					<0.001
I	2 (28.6%)	13 (34.2%)	6 (20.0%)	7 (25.9%)		16 (12.7%)	98 (28.0%)	33 (9.0%)	57 (20.4%)	
II	1 (14.3%)	4 (10.5%)	3 (10.0%)	6 (22.2%)		27 (21.4%)	109 (31.1%)	57 (15.5%)	84 (30.1%)	
III	3 (42.9%)	20 (52.6%)	18 (60.0%)	12 (44.4%)		72 (57.1%)	122 (34.9%)	232 (63.0%)	122 (43.7%)	
IV	1 (14.3%)	1 (2.6%)	3 (10.0%)	2 (7.4%)		11 (8.7%)	21 (6.0%)	46 (12.5%)	16 (5.7%)	
**Chemotherapy**	3 (42.9%)	13 (34.2%)	18 (60.0%)	12 (44.4%)	0.210	56 (44.4%)	170 (48.6%)	172 (46.7%)	139 (49.8%)	0.735

LRS, lymphoid radiomics score; MRS, myeloid radiomics score. 1, L-LRS and L-MRS; 2, H-LRS and L-MRS; 3, L-LRS and H-MRS; 4, H-LRS and H-MRS.

Supplementary Table 16. The predictive value of radiomics signature is compared according to cut-off values determined by different methods.

Variables	Cut-off value from Youden's index	The median	The upper quartile	The lower quartile
**Cut-off value**				
LRS	−0.1293	0.1463	-0.6966	0.8603
MRS	−0.2604	-0.0659	-0.7254	0.6153
**Predictive value for DFS in patients with radical surgery, n=2,297, HR (P value)**				
Patients (High vs Low)	1317 vs 980	1115 vs 1182	1630 vs 667	669 vs 1628
LRS (dichotomy)	0.463 (<0.00001)	0.472 (<0.00001)	0.475 (<0.00001)	0.511 (<0.00001)
Patients (High vs Low)	1329 vs 968	1191 vs 1106	1647 vs 650	690 vs 1607
MRS (dichotomy)	1.857 (<0.00001)	1.767 (<0.00001)	1.769 (<0.00001)	1.517 (<0.00001)
**Predictive value for DFS in patients with radical surgery, n=2,297, C-index**				
LRS (dichotomy)	0.599	0.595	0.585	0.568
MRS (dichotomy)	0.576	0.573	0.557	0.547
**Predictive value for OS in patients with radical surgery, n=2,297, HR (P value)**				
LRS (dichotomy)	0.451 (<0.00001)	0.452 (<0.00001)	0.472 (<0.00001)	0.518 (<0.00001)
MRS (dichotomy)	1.939 (<0.00001)	1.853 (<0.00001)	1.772 (<0.00001)	1.566 (<0.00001)
**Predictive value for OS in patients with radical surgery, n=2,297, C-index**				
LRS (dichotomy)	0.602	0.599	0.585	0.567
MRS (dichotomy)	0.581	0.578	0.557	0.548
**Predictive value of LRS for immunotherapy, n=261, Low (%) vs High (%)**				
Patients (High vs Low)	160 vs 101	124 vs 137	66 vs 195	217 vs 44
CR (%)	3.0% vs 13.8%	5.1% vs 14.5%	1.5% vs 12.3%	7.8% vs 18.2%
PR (%)	10.9% vs 25.0%	14.6% vs 25.0%	10.6% vs 22.6%	16.6% vs 34.1%
SD (%)	27.7% vs 18.8%	26.3% vs 17.7%	31.8% vs 19.0%	23.0% vs 18.2%
PD (%)	58.4% vs 42.5%	54.0% vs 42.7%	56.1% vs 46.2%	52.5% vs 29.5%
**Predictive value of MRS for immunotherapy, n=261, Low (%) vs High (%)**				
Patients (High vs Low)	179 vs 82	161 vs 100	206 vs 55	66 vs 195
CR (%)	17.1% vs 6.1%	15.0% vs 6.2%	14.5% vs 8.3%	12.3% vs 1.5%
PR (%)	29.3% vs 15.1%	25.0% vs 16.1%	34.5% vs 15.5%	21.5% vs 13.6%
SD (%)	22.0% vs 22.3%	22.0% vs 22.4%	25.5% vs 21.4%	21.5% vs 24.2%
PD (%)	31.7% vs 56.4%	38.0% vs 55.3%	25.5% vs 54.9%	44.6% vs 60.6%
**AUC of immunotherapy response**				
LRS (dichotomy)	0.643 (0.572-0.714)	0.620 (0.545-0.694)	0.604 (0.533-0.676)	0.595 (0.515-0.674)
MRS (dichotomy)	0.631 (0.554-0.708)	0.601 (0.524-0.678)	0.602 (0.523-0.681)	0.586 (0.513-0.658)
**Predictive value of LRS/MRS for immunotherapy, n=261, 1(%), 2(%) 3(%), or 4(%)**				
Patients (1 vs 2 vs 3 vs 4)	22 vs 60 vs 79 vs 100	42 vs 58 vs 95 vs 66	11 vs 44 vs 55 vs 151	161 vs 34 vs 56 vs 10
CR (%)	9.1 vs 20.0 vs 1.3 vs 10.0	4.8 vs 22.4 vs 5.3 vs 7.6	9.1 vs 15.9 vs 0 vs 11.3	9.9 vs 23.5 vs 1.8 vs 0
PR (%)	18.2 vs 33.3 vs 8.9 vs 20.0	23.8 vs 25.9 vs 10.5 vs 24.2	18.2 vs 38.6 vs 9.1 vs 17.9	19.3 vs 32.4 vs 8.9 vs 40.0
SD (%)	36.4 vs 16.7 vs 25.3 vs 20.0	31.0 vs 15.5 vs 24.2 vs 19.7	45.5 vs 20.5 vs 29.1 vs 18.5	22.4 vs 17.6 vs 25.0 vs 20.0
PD (%)	36.4 vs 30.0 vs 64.6 vs 50.0	40.5 vs 36.2 vs 60.0 vs 48.5	27.3 vs 25.0 vs 61.8 vs 52.3	48.4 vs 26.5 vs 64.3% vs 40.0

LRS, lymphoid radiomics score; MRS, myeloid radiomics score. 1, L-LRS and L-MRS; 2, H-LRS and L-MRS; 3, L-LRS and H-MRS; 4, H-LRS and H-MRS.

CR: complete response, PR: partial response, SD: stable disease, PD: progressive disease.

Supplementary Table 17. Univariate association of RS, clinicopathological characteristics with disease-free and overall survival in the training cohort.

Variables	Disease-free survival		Overall survival	
	HR (95%CI)	*p*	HR (95%CI)	*p*
LRS (high vs. low)	0.429 (0.291-0.633)	<0.0001	0.345 (0.212-0.561)	<0.0001
MRS (high vs. low)	2.022 (1.333-3.069)	0.001	2.533 (1.482-4.331)	0.001
Age (years) (≥60 vs. <60)	0.937 (0.631-1.391)	0.748	0.757 (0.462-1.240)	0.269
Sex (male vs. female)	1.043 (0.694-1.566)	0.841	1.334 (0.798-2.229)	0.271
Tumor size (>4 cm vs. ≤4 cm)	1.831 (1.235-2.715)	0.003	2.153 (1.324-3.503)	0.002
Tumor location	1.053 (0.838-1.324)	0.656	0.944 (0.719-1.240)	0.680
Differentiation	1.379 (1.011-1.881)	0.042	1.504 (1.022-2.214)	0.039
Lauren type	1.005 (0.684-1.477)	0.980	0.980 (0.613-1.567)	0.933
CEA (elevated vs. normal)	2.033 (1.176-3.515)	0.011	2.212 (1.162-4.213)	0.016
CA19-9 (elevated vs. normal)	1.477 (0.878-2.485)	0.142	1.001 (1.000-1.003)	0.081
Stage (IV vs. III vs. II vs. I)	2.123 (1.645-2.739)	<0.0001	2.954 (2.095-4.144)	<0.0001
Chemotherapy (yes vs. no)	0.686 (0.466-1.010)	0.056	0.489 (0.302-0.792)	0.004

RS, radiomics score; LRS, lymphoid radiomics score; MRS, myeloid radiomics score.

Supplementary Table 18. Univariate association of RS, clinicopathological characteristics with disease-free and overall survival in the internal validation cohort 1.

Variables	Disease-free survival		Overall survival	
	HR (95%CI)	*p*	HR (95%CI)	*p*
LRS (high vs. low)	0.330 (0.207-0.528)	<0.0001	0.322 (0.184-0.565)	<0.0001
MRS (high vs. low)	2.219 (1.305-3.772)	0.003	3.034 (1.525-6.034)	0.002
Age (years) (≥60 vs. <60)	1.053 (0.669-1.655)	0.824	0.890 (0.517-1.532)	0.674
Sex (male vs. female)	1.366 (0.812-2.297)	0.240	1.739 (0.896-3.374)	0.102
Tumor size (>4 cm vs. ≤4 cm)	1.867 (1.173-2.971)	0.008	1.819 (1.046-3.163)	0.034
Tumor location	0.950 (0.730-1.236)	0.702	0.963 (0.703-1.319)	0.814
Differentiation	0.966 (0.708-1.317)	0.826	1.035 (0.711-1.507)	0.856
Lauren type	0.913 (0.581-1.434)	0.692	1.197 (0.692-2.069)	0.521
CEA (elevated vs. normal)	1.365 (0.718-2.593)	0.343	1.461 (0.687-3.105)	0.324
CA19-9 (elevated vs. normal)	1.003 (0.998-1.009)	0.220	1.185 (0.535-2.624)	0.676
Stage (IV vs. III vs. II vs. I)	2.065 (1.520-2.804)	<0.0001	2.826 (1.894-4.216)	<0.0001
Chemotherapy (yes vs. no)	0.876 (0.558-1.376)	0.567	1.169 (0.684-1.997)	0.568

RS, radiomics score; LRS, lymphoid radiomics score; MRS, myeloid radiomics score.

Supplementary Table 19. Univariate association of RS, clinicopathological characteristics with disease-free and overall survival in the internal validation cohort 2.

Variables	Disease-free survival		Overall survival	
	HR (95%CI)	*p*	HR (95%CI)	*p*
LRS (high vs. low)	0.476 (0.378-0.600)	<0.0001	0.466 (0.359-0.603)	<0.0001
MRS (high vs. low)	1.640 (1.281-2.099)	<0.0001	1.652 (1.252-2.181)	<0.0001
Age (years) (≥60 vs. <60)	1.139 (0.901 -1.440)	0.276	1.364 (1.053-1.767)	0.019
Sex (male vs. female)	0.954 (0.746-1.219)	0.705	0.978 (0.743-1.286)	0.872
Tumor size (>4 cm vs. ≤4 cm)	1.191 (0.937-1.514)	0.152	1.257 (0.964-1.640)	0.091
Tumor location	1.040 (0.893-1.211)	0.617	1.039 (0.879-1.229)	0.654
Differentiation	1.164 (0.997-1.359)	0.055	1.292 (1.078-1.548)	0.006
Lauren type	1.187 (0.940-1.499)	0.149	1.085 (0.837-1.406)	0.537
CEA (elevated vs. normal)	2.556 (1.859-3.515)	<0.0001	2.701 (1.900-3.841)	<0.0001
CA19-9 (elevated vs. normal)	2.742 (2.091-3.595)	<0.0001	2.643 (1.954-3.574)	<0.0001
Stage (III vs. II vs. I)	2.416 (2.037-2.865)	<0.0001	2.641 (2.157-3.242)	<0.0001
Chemotherapy (yes vs. no)	1.108 (0.880 -1.395)	0.384	1.106 (0.854-1.432)	0.444

RS, radiomics score; LRS, lymphoid radiomics score; MRS, myeloid radiomics score.

Supplementary Table 20. Univariate association of RS, clinicopathological characteristics with disease-free and overall survival in the external validation cohort 1.

Variables	Disease-free survival		Overall survival	
	HR (95%CI)	*p*	HR (95%CI)	*p*
LRS (high vs. low)	0.310 (0.164-0.588)	<0.0001	0.274 (0.136-0.552)	<0.001
MRS (high vs. low)	3.772 (1.825-7.796)	<0.001	4.679 (2.089-10.480)	<0.001
Age (years) (≥60 vs. <60)	0.752 (0.394-1.435)	0.388	0.736 (0.368-1.472)	0.377
Sex (male vs. female)	1.742 (0.799-3.794)	0.163	2.081 (0.860-5.040)	0.104
Tumor size (>4 cm vs. ≤4 cm)	2.748 (1.442-5.237)	0.002	3.049 (1.518-6.128)	0.002
Tumor location	0.760 (0.538-1.073)	0.119	0.748 (0.517-1.081)	0.123
Differentiation	1.473 (0.646-3.359)	0.357	1.358 (0.592-3.114)	0.470
Lauren type	1.234 (0.634-2.402)	0.536	1.169 (0.578-2.363)	0.664
CEA (elevated vs. normal)	1.091 (0.482-2.473)	0.834	1.250 (0.544-2.875)	0.599
CA19-9 (elevated vs. normal)	4.564 (2.287-9.108)	<0.0001	4.086 (1.922-8.687)	<0.0001
Stage (IV vs. III vs. II vs. I)	2.499 (1.616-3.865)	<0.0001	2.318 (1.485-3.617)	<0.0001
Chemotherapy (yes vs. no)	1.902 (1.009-3.584)	0.047	2.004 (1.012-3.969)	0.046

RS, radiomics score; LRS, lymphoid radiomics score; MRS, myeloid radiomics score.

Supplementary Table 21. Univariate association of RS, clinicopathological characteristics with disease-free and overall survival in the external validation cohort 2.

Variables	Disease-free survival		Overall survival	
	HR (95%CI)	*p*	HR (95%CI)	*p*
LRS (high vs. low)	0.522 (0.441-0.619)	<0.0001	0.509 (0.428-0.604)	<0.0001
MRS (high vs. low)	1.662 (1.392-1.984)	<0.0001	1.738 (1.451-2.082)	<0.0001
Age (years) (≥60 vs. <60)	1.294 (1.092-1.533)	0.003	1.305 (1.099-1.551)	0.002
Sex (male vs. female)	1.023 (0.852-1.228)	0.811	1.028 (0.854-1.238)	0.772
Tumor size (>4 cm vs. ≤4 cm)	1.906 (1.583-2.294)	<0.0001	1.979 (1.637-2.392)	<0.0001
Tumor location	0.935 (0.853-1.024)	0.149	0.933 (0.850-1.024)	0.143
Differentiation	1.240 (1.009-1.524)	0.041	1.228 (0.998-1.511)	0.052
Lauren type	1.287 (1.070-1.549)	0.007	1.289 (1.069-1.555)	0.008
CEA (elevated vs. normal)	1.573 (1.290-1.917)	<0.0001	1.582 (1.294-1.933)	<0.0001
CA19-9 (elevated vs. normal)	1.873 (1.544-2.270)	<0.0001	1.908 (1.570-2.318)	<0.0001
Stage (IV vs. III vs. II vs. I)	2.244 (2.001-2.516)	<0.0001	2.253 (2.006-2.531)	<0.0001
Chemotherapy (yes vs. no)	0.990 (0.836 -1.174)	0.911	1.020 (0.858-1.212)	0.823

RS, radiomics score; LRS, lymphoid radiomics score; MRS, myeloid radiomics score.

Supplementary Table 22. Univariate association of RS, clinicopathological characteristics with disease-free and overall survival in the prospective validation cohort

Variables	Disease-free survival		Overall survival	
	HR (95%CI)	*p*	HR (95%CI)	*p*
LRS (high vs. low)	0.218 (0.110-0.435)	<0.0001	0.180 (0.077-0.418)	<0.0001
MRS (high vs. low)	3.591 (1.796-7.178)	<0.001	4.249 (1.828-9.872)	0.001
Age (years) (≥60 vs. <60)	1.742 (0.954-3.181)	0.071	2.658 (1.281-5.515)	0.009
Sex (male vs. female)	0.971 (0.59-1.819)	0.927	1.015 (0.489-2.105)	0.969
Tumor size (>4 cm vs. ≤4 cm)	3.785 (2.051-6.984)	<0.0001	4.066 (1.986-8.323)	<0.001
Tumor location	1.007 (0.708-1.431)	0.971	0.886 (0.593-1.323)	0.553
Differentiation	1.368 (0.784-2.390)	0.270	1.000 (0.569-1.758)	0.999
Lauren type	1.358 (0.697-2.646)	0.368	0.976 (0.470-2.023)	0.947
CEA (elevated vs. normal)	2.451 (0.874-6.870)	0.088	2.331 (0.710-7.655)	0.163
CA19-9 (elevated vs. normal)	2.590 (1.150-5.831)	0.022	2.602 (1.070-6.327)	0.035
Stage (IV vs. III vs. II vs. I)	3.232 (2.121-4.924)	<0.0001	2.813 (1.778-4.451)	<0.0001
Chemotherapy	2.822 (1.420-5.611)	0.003	1.988 (0.940-4.206)	0.072

RS, radiomics score; LRS, lymphoid radiomics score; MRS, myeloid radiomics score.

Supplementary Table 23. Clinical characteristics of patients according to the LRS, MRS, and combined score in the radiogenomics cohort.

Variables	LRS, n =42			MRS, n = 42			Combined score (LRS/MRS), n = 42				
	low LRS	high LRS	*P*	low MRS	high MRS	*P*	1 (−/−)	2 (+/−)	3 (−/+)	4 (+/+)	*P*
**Median age (range)**	68 (63-74)	67 (58-70)	0.344	68 (63-72)	67 (58-69)	0.168	70 (67-/)	68 (59-71)	68 (61-74)	59 (57-67)	0.202
**Male (%)**	14 (93.3%)	22 (81.5%)	0.293	16 (84.2%)	20 (87.0%)	0.800	3 (100%)	13 (81.3%)	11 (91.7%)	9 (81.8%)	0.742
**Stage (%)**			0.840			0.278					0.565
I	0 (0.0%)	1 (3.7%)		1 (5.3%)	0 (0.0%)		0 (0.0%)	1 (6.3%)	0 (0.0%)	0 (0.0%)	
II	2 (13.3%)	5 (18.5%)		5 (26.3%)	2 (8.7%)		0 (0.0%)	5 (31.3%)	2 (16.7%)	0 (0.0%)	
III	12 (80.0%)	19 (70.4%)		12 (63.2%)	19 (82.6%)		3 (100%)	9 (56.3%)	9 (75.0%)	10 (90.9%)	
IV	1 (6.7%)	2 (7.4%)		1 (5.3%)	2 (8.7%)		0 (0.0%)	1 (6.3%)	1 (8.3%)	1 (9.1%)	

Supplementary Table 24. Comparing the prediction accuracy of the integrated nomogram with RS and TNM stage in the training and validation cohorts.

Variable	Disease-free survival		Overall survival	
	C-Index (95% CI)	*P*	C-Index (95% CI)	*P*
**Training cohort**		<0.001		<0.001
Nomogram	0.735 (0.686-0.784)		0.816 (0.774-.858)	
LRS	0.637 (0.581-0.693)		0.661 (0.598-0.724)	
MRS	0.627 (0.571-0.683)		0.645 (0.580-0.710)	
Stage	0.662 (0.613-0.711)		0.723 (0.679-0.767)	
**Internal validation cohort 1**		<0.001		<0.001
Nomogram	0.726 (0.672-0.780)		0.766 (0.706-0.826)	
LRS	0.633 (0.564-0.702)		0.621 (0.547-0.695)	
MRS	0.577 (0.410-0.644)		0.615 (0.541-0.689)	
Stage	0.656 (0.600-0.712)		0.699 (0.642-0.756)	
**Internal validation cohort 2**		<0.001		<0.001
Nomogram	0.748 (0.722-0.774)		0.754 (0.724-0.784)	
LRS	0.627 (0.592-0.662)		0.639 (0.600-0.678)	
MRS	0.570 (0.534-0.606)		0.581 (0.541-0.621)	
Stage	0.686 (0.661-0.711)		0.687 (0.658-0.716)	
**External validation cohort 1**		<0.001		<0.001
Nomogram	0.767 (0.705-0.829)		0.779 (0.716-0.842)	
LRS	0.698 (0.627-0.769)		0.699 (0.622-0.776)	
MRS	0.653 (0.574-0.732)		0.662 (0.576-0.748)	
Stage	0.692 (0.620-0.764)		0.684 (0.608-0.760)	
**External validation cohort 2**		<0.001		<0.001
Nomogram	0.716 (0.695-0.737)		0.715 (0.693-0.737)	
LRS	0.620 (0.596-0.644)		0.619 (0.594-0.644)	
MRS	0.577 (0.552-0.602)		0.580 (0.555-0.605)	
Stage	0.676 (0.656-0.696)		0.675 (0.654-0.696)	
**Prospective validation cohort**		<0.001		<0.001
Nomogram	0.839 (0.779-0.899)		0.841 (0.782-0.900)	
LRS	0.780 (0.707-0.853)		0.803 (0.730-0.876)	
MRS	0.693 (0.621-0.765)		0.677 (0.600-0.754)	
Stage	0.731 (0.658-0.804)		0.709 (0.628-0.790)	

RS, radiomics score; LRS, lymphoid radiomics score; MRS, myeloid radiomics score.

Supplementary Table 25. Clinical characteristics of patients according to the LRS, MRS, and combined score in the immunotherapy cohort 1 (SMU).

Variables	LRS, n =198			MRS, n = 198			Combined score (LRS/MRS), n = 198				
	low LRS	high LRS	*P*	low MRS	high MRS	*P*	1 (−/−)	2 (+/−)	3 (−/+)	4 (+/+)	*P*
**Median age (range)**	54 (45-64)	56 (48-66)	0.301	57 (49-65)	55 (46-65)	0.393	58 (52-63)	56 (49-67)	54 (44-65)	56 (48-66)	0.496
**Male (%)**	40 (58.8%)	75 (57.7%)	0.879	36 (62.1%)	79 (56.4%)	0.467	7 (53.8%)	29 (64.4%)	33 (60.0%)	46 (54.1%)	0.693
**Stage (%)**			0.023			0.079					0.071
II	2 (2.9%)	15 (11.5%)		8 (13.8%)	9 (6.4%)		1 (7.7%)	7 (15.6%)	1 (1.8%)	8 (9.4%)	
III	12 (17.6%)	35 (26.9%)		17 (29.3%)	30 (21.4%)		2 (15.4%)	15 (33.3%)	10 (18.2%)	20 (23.5%)	
IV	54 (79.4%)	80 (61.5%)		33 (56.9%)	101 (72.1%)		10 (76.9%)	23 (51.1%)	44 (80.0%)	57 (67.1%)	
**Response (%)**			0.001			<0.0001					<0.0001
CR	3 (4.4%)	21 (16.2%)		13 (22.4%)	11 (7.9%)		2 (15.3%)	11 (24.5%)	1 (1.8%)	10 (11.7%)	
PR	8 (11.8%)	32 (24.6%)		17 (29.3%)	23 (16.5%)		3 (23.1%)	14 (31.1%)	5 (9.1%)	18 (21.2%)	
SD	14 (20.6%)	19 (14.6%)		9 (15.5%)	24 (17.1%)		3 (23.1%)	6 (13.3%)	11 (20.0%)	13 (15.3%)	
PD	43 (63.2%)	58 (44.6%)		19 (32.8%)	82 (58.5%)		5 (38.5%)	14 (31.1%)	38 (69.1%)	44 (51.8%)	
**Treatment line (%)**			0.538			0.014					0.071
First line	42 (61.8%)	72 (55.4%)		39 (67.2%)	75 (53.6%)		10 (76.9%)	29 (64.4%)	32 (58.2%)	43 (50.6%)	
Second line	14 (20.6%)	34 (26.2%)		15 (25.9%)	33 (23.6%)		2 (15.4%)	13 (28.9%)	12 (21.8%)	21 (24.7%)	
Third line	12 (17.6%)	24 (18.5%)		4 (6.9%)	32 (22.9%)		1 (7.7%)	3 (6.7%)	11 (20.0%)	21 (24.7%)	

LRS, lymphoid radiomics score; MRS, myeloid radiomics score. 1, L-LRS and L-MRS; 2, H-LRS and L-MRS; 3, L-LRS and H-MRS; 4, H-LRS and H-MRS.

Supplementary Table 26. Clinical characteristics of patients according to the LRS, MRS, and combined score in the immunotherapy cohort 2 (GPHCM).

Variables	LRS, n =63			MRS, n = 63			Combined score (LRS/MRS), n = 63				
	low LRS	high LRS	*P*	low MRS	high MRS	*P*	1 (−/−)	2 (+/−)	3 (−/+)	4 (+/+)	*P*
**Median age (range)**	58 (48-65)	62 (48-66)	0.343	64 (50-68)	58 (48-64)	0.384	68 (60-70)	59 (47-66)	55 (48-60)	62 (50-71)	0.078
**Male (%)**	20 (60.6%)	14 (46.7%)	0.268	15 (62.5%)	19 (48.7%)	0.287	8 (88.9%)	7 (46.7%)	12 (50.0%)	7 (46.7%)	0.157
**Stage (%)**			0.050			0.514					0.002
III	5 (15.2%)	11 (36.7%)		5 (20.8%)	11 (28.2%)		3 (33.3%)	2 (13.3%)	2 (8.3%)	9 (60.0%)	
IV	28 (84.8%)	19 (63.3%)		19 (79.2%)	28 (71.8%)		6 (66.7%)	13 (86.7%)	22 (91.7%)	6 (40.0%)	
**Response (%)**			0.180			0.107					0.193
CR	0 (0.0%)	1 (3.3%)		1 (4.2%)	0 (0.0%)		0 (0.0%)	1 (6.7%)	0 (0.0%)	0 (0.0%)	
PR	3 (9.1%)	8 (26.7%)		7 (29.2%)	4 (10.3%)		1 (11.1%)	6 (40.0%)	2 (8.3%)	2 (13.3%)	
SD	14 (42.4%)	11 (36.7%)		9 (37.4%)	16 (41.0%)		5 (55.6%)	4 (26.7%)	9 (37.5%)	7 (46.7%)	
PD	16 (48.5%)	10 (33.3%)		7 (29.2%)	19 (48.7%)		3 (33.3%)	4 (26.7%)	13 (54.2%)	6 (40.0%)	
**Treatment line (%)**			0.032			0.048					0.071
First line	22 (66.7%)	11 (36.7%)		10 (41.7%)	23 (59.0%)		5 (55.6%)	5 (33.3%)	17 (70.8%)	6 (40.0%)	
Second line	10 (30.3%)	14 (46.7%)		9 (37.5%)	15 (38.5%)		3 (33.3%)	6 (40.0%)	7 (29.2%)	8 (53.3%)	
Third line	1 (3.0%)	5 (16.7%)		5 (20.8%)	1 (2.6%)		1 (11.1%)	4 (26.7%)	0 (0.0%)	1 (6.7%)	

LRS, lymphoid radiomics score; MRS, myeloid radiomics score. 1, L-LRS and L-MRS; 2, H-LRS and L-MRS; 3, L-LRS and H-MRS; 4, H-LRS and H-MRS.

Supplementary Table 27. Immunotherapy response of patients according to the LRS, MRS, and combined score in different treatment types.

Variables	LRS, n =261			MRS, n = 261			Combined score (LRS/MRS), n = 261				
	low LRS	high LRS	*P*	low MRS	high MRS	*P*	1 (−/−)	2 (+/−)	3 (−/+)	4 (+/+)	*P*
**Neoadjuvant therapy**			0.250			0.061					0.169
CR	2 (18.2%)	10 (28.6%)		7 (43.8%)	5 (16.7%)		1 (33.3%)	6 (46.2%)	1 (12.5%)	4 (18.2%)	
PR	3 (27.3%)	16 (45.7%)		7 (43.8%)	12 (40.0%)		1 (33.3%)	6 (46.2%)	2 (25.0%)	10 (45.5%)	
SD	2 (18.2%)	5 (14.3%)		2 (12.5%)	5 (16.7%)		1 (33.3%)	1 (7.7%)	1 (12.5%)	4 (18.2%)	
PD	4 (36.4%)	4 (11.4%)		0	8 (26.7%)		0	0	4 (50.0%)	4 (18.2%)	
**Adjuvant therapy**			0.052			0.952					0.494
CR	0	8 (30.8%)		4 (28.6%)	4 (20.0%)		0	4 (36.4%)	0	4 (26.7%)	
PR	0	5 (19.2%)		2 (14.3%)	3 (15.0%)		0	2 (18.2%)	0	3 (20.0%)	
SD	2 (25.0%)	6 (23.1%)		3 (21.4%)	5 (25.0%)		1 (33.3%)	2 (18.2%)	1 (20.0%)	4 (26.7%)	
PD	6 (75.0%)	7 (26.9%)		5 (35.7%)	8 (40.0%)		2 (66.7%)	3 (27.3%)	4 (80.0%)	4 (26.7%)	
**For advanced disease**			0.115			0.001					0.010
CR	1 (1.2)	4 (4.0%)		3 (5.8%)	2 (1.6%)		1 (6.3)	2 (5.6%)	0	2 (3.2%)	
PR	8 (9.8%)	19 (19.2%)		15 (28.8%)	12 (9.3%)		3 (18.8%)	12 (33.3%)	5 (7.6%)	7 (11.1%)	
SD	24 (29.3%)	19 (19.2%)		13 (25.0%)	30 (23.3%)		6 (37.5%)	7 (19.4%)	18 (27.3%)	12 (19.0%)	
PD	49 (59.8%)	57 (57.6%)		21 (40.4%)	85 (65.9%)		6 (37.5%)	15 (41.7%)	43 (65.2%)	42 (66.7%)	

CR: complete response, PR: partial response, SD: stable disease, PD: progressive disease.

Supplementary Table 28. Antibody sources and staining conditions.

Markers	Main target	Antibody source	Species	Dilution	DAB dyeing time	Antigen Retrieval	Cellular localization
CD3	Pan T lymphocyte	NeoMarker, clone SP7	Rabbit monoclonal	1:300	1.0 min	Citrate buffer (pH 6.0) microwave 20min	Membranous
CD8	Cytotoxic T lymphocyte	NeoMarker, clone SP16	Rabbit monoclonal	1:200	1.5 min	Citrate buffer (pH 6.0) microwave 20min	Membranous
CD66b	Myelocyte	BD Pharmingen	Mouse monoclonal	1:200	1.0 min	Citrate buffer (pH 6.0) microwave 20min	Membranous

min: minute; sec: second. DAB: diaminobenzidine.

Supplementary Table 29. Imaging Biomarker Standardization Initiative (IBSI) reporting structure of the study.

**Patients**	
Region of interest	CT positive lesion in stomach
Patient Preparation	Patients were required to drink enough water before CT examination to ensure sufficient distention of gastric cavity in CT images
Computed tomography (CT) developing agent	iodinated contrast material
**Acquisition and Reconstruction**	
Protocol	The acquisition parameters are as follows: 120 kV; 150-190 mAs; 0.5- or 0.4-second rotation time; detector collimation: 8×2.5 mm or 64×0.625 mm; field of view, 350×350 mm; matrix, 512×512. After routine non-enhanced CT, arterial and portal venous-phase contrast-enhanced CT were performed after delays of 28 s and 60 s following intravenous administration of 90 - 100 ml of iodinated contrast material (Ultravist 370, Bayer Schering Pharma, Berlin, Germany) at a rate of 3.0 or 3.5 ml/s with a pump injector (Ulrich CT Plus 150, Ulrich Medical, Ulm, Germany). Contrast-enhanced CT was reconstructed with a reconstruction thickness of 2.5 mm. Portal venous phase CT images (thickness: 2.5 mm) were retrieved from the picture archiving and communication system (PACS) (Carestream, Canada) for image feature extraction because of well differentiation of the tumor tissue from the adjacent tissue.
Scanner type	multidetector row CT systems
**Delineation**	
Software	ITK-SNAP software (version 3.6; www.itksnap.org).
ROI definition	Standard 2D ROI tools
Number of experts	2 + 1 (2 experienced radiologists participated in independent delineations, followed by 1 senior radiologist cross-validation if necessary)
Reference image	CT
**Radiomics feature extraction**	
Software	Matlab R2016a (The MathWorks Inc.)
Package	radiomics analysis package (https://github.com/mvallieres/radiomics/)
Method	Reads the DICOM content of a single directory; Equal-probability quantization on the region of interest (ROI); computes Lloyd-Max quantization on the region of interest (ROI) of an input volume; computes uniform quantization on the region of interest (ROI) of an input volume; applies the intensity normalization scheme; Computation of the smallest box containing region of interest (ROI), if

	necessary (ROIbox); Wavelet band-pass filtering (WBPF); Isotropic resampling; Quantization of intensity dynamic range.
Discretization	Bin width and LoG filters
Bin width	25 for CT
Kernels of the filter	Gaussian spatial band-pass filter (∇2G)
Biomarker set	intensity features, shape features, gray Level Co-occurrence Matrix-based (GLCM) features, gray Level Run Length Matrix-based (GLRLM) features, gray Level Size Zone Matrix-based (GLSZM) features and neighborhood Gray Tone Difference Matrix–based (NGTDM) features.
Exclusion criteria	ICC smaller than 0.75

Supplementary References

1. Jiang Y, Zhang Q, Hu Y, Li T, Yu J, Zhao L, et al. ImmunoScore Signature: A Prognostic and Predictive Tool in Gastric Cancer. Ann Surg. 2018;267(3):504-13. Epub 2016/12/22. doi: 10.1097/SLA.0000000000002116. PubMed PMID: 28002059.
2. Jiang Y, Xie J, Han Z, Liu W, Xi S, Huang L, et al. Immunomarker Support Vector Machine Classifier for Prediction of Gastric Cancer Survival and Adjuvant Chemotherapeutic Benefit. Clin Cancer Res. 2018;24(22):5574-84. Epub 2018/07/26. doi: 10.1158/1078-0432.CCR-18-0848. PubMed PMID: 30042208.
3. Jiang Y, Liu W, Li T, Hu Y, Chen S, Xi S, et al. Prognostic and Predictive Value of p21-activated Kinase 6 Associated Support Vector Machine Classifier in Gastric Cancer Treated by 5-fluorouracil/Oxaliplatin Chemotherapy. EBioMedicine. 2017;22:78-88. Epub 2017/07/09. doi: 10.1016/j.ebiom.2017.06.028. PubMed PMID: 28687498; PubMed Central PMCID: PMCPMC5552213.
4. Jiang Y, Yuan Q, Lv W, Xi S, Huang W, Sun Z, et al. Radiomic signature of (18)F fluorodeoxyglucose PET/CT for prediction of gastric cancer survival and chemotherapeutic benefits. Theranostics. 2018;8(21):5915-28. Epub 2019/01/08. doi: 10.7150/thno.28018. PubMed PMID: 30613271; PubMed Central PMCID: PMCPMC6299427.
5. Yushkevich PA, Piven J, Hazlett HC, Smith RG, Ho S, Gee JC, et al. User-guided 3D active contour segmentation of anatomical structures: significantly improved efficiency and reliability. Neuroimage. 2006;31(3):1116-28. Epub 2006/03/21. doi: 10.1016/j.neuroimage.2006.01.015. PubMed PMID: 16545965.
